# The bead process for beta ensembles

**DOI:** 10.1007/s00440-021-01034-8

**Published:** 2021-03-13

**Authors:** Joseph Najnudel, Bálint Virág

**Affiliations:** 1 School of Mathematics, University of Bristol, Fry Building, Woodland Road, Bristol, BS8 1UG UK; 2 Department of Mathematics, University of Toronto, 40 St George St., Toronto, ON M5S 2E4 Canada

**Keywords:** 60B20, 60J55, 60F05, 60J05

## Abstract

The bead process introduced by Boutillier is a countable interlacing of the ${\text {Sine}}_2$ point processes. We construct the bead process for general ${\text {Sine}}_{\beta }$ processes as an infinite dimensional Markov chain whose transition mechanism is explicitly described. We show that this process is the microscopic scaling limit in the bulk of the Hermite $\beta$ corner process introduced by Gorin and Shkolnikov, generalizing the process of the minors of the Gaussian Unitary and Orthogonal Ensembles. In order to prove our results, we use bounds on the variance of the point counting of the circular and the Gaussian beta ensembles, proven in a companion paper (Najnudel and Virág in Some estimates on the point counting of the Circular and the Gaussian Beta Ensemble, 2019).

## Introduction

In Boutillier [6], a remarkable family of point processes on ${\mathbb {R}} \times {\mathbb {Z}}$, called *bead processes*, and indexed by a parameter $\gamma \in (-1,1)$, has been defined. They enjoy the following properties:

Interlacing: The points of two consecutive lines interlace with each other.
Invariance: The distribution of the point process is invariant and ergodic under the natural action of ${\mathbb {R}}\times {\mathbb {Z}}$ by translation.
Parameters: The expected number of points in any interval is proportional to its length. Given that (0, 0) is in the process, the expected value of the first positive point on line 1 is proportional to $\arccos \gamma$.
Gibbs property: The distribution of any point *X*, given the other points, is uniform on the interval which is allowed by the interlacing property.

It is an open problem whether these properties determine the point process uniquely. Such uniqueness results exist for tilings, see Sheffield [17].

Existence was shown by Boutillier, who considers a determinantal process with an explicit kernel. Its restriction to a line is the standard sine-kernel process. Thus the above description proposes to be the purest probabilistic definition of the Gaudin–Mehta sine kernel process limit of the bulk eigenvalues of the Gaussian Unitary Ensemble (GUE).

Boutillier’s result relies on taking limits of tilings on the torus. Since then, works starting with Johansson and Nordenstam [11] showed that the consecutive minor eigenvalues of the Gaussian Unitary Ensemble also converge to the bead process, where the tilt depends on the global location within the Wigner semicircle. These results have been refined and generalized in Adler et al. [1]. However, the corresponding questions remained open for other matrix ensembles, as the Gaussian Orthogonal Ensemble (GOE), and the Gaussian Symplectic Ensemble (GSE):

- Is there a limit of the eigenvalue minor process?
- Is there a simple characterization as for $\beta =2$?
- Can one derive formulas related to the distribution of beads?

One of the main goals of this paper is to answer these questions positively. The limiting process is defined as an infinite-dimensional Markov chain, the transition from one line to the next being explicitly described. This transition can be viewed as a generalization of the limit, when the dimension *n* goes to infinity, of the random reflection walk on the unitary group *U*(*n*). This walk is the unitary analogue of the random transposition walk studied, for example, in Diaconis and Shahshahani [7], Berestycki and Durrett [3] and Bormashenko [5].

The natural generalization of the transpositions to the setting of the orthogonal group corresponds to the reflections. The orthogonal matrix corresponding to the reflection across the plane with normal unit vector *v* is $I-2vv^*$. To further generalize to the unitary group, we proceed as follows: given a fixed unit complex number $\eta$ and a unit vector *v*, we define the *complex reflection* across *v* with angle $\arg (\eta )$ as the isometry whose matrix is given by $I+(\eta -1)vv^*$. The random reflection walk $(Y_k)_{k \ge 1}$ on the unitary group *U*(*n*) is then defined by $Y_k=X_1\dots X_k$, where $(X_j)_{j \ge 1}$ are independent reflections for which *v* is chosen according to uniform measure on the complex unit sphere, and $\eta$ is fixed.

Note that since the multiplicative increments of the walk are invariant under conjugation by any group element, it follows that ${\bar{Y}}_k$, the conjugacy class of $Y_k$, also follows a random walk. This, of course, is given by the eigenvalues of $Y_k$; the transition mechanism can be computed as follows. Assuming that the eigenvalues $u_j$ of ${\bar{Y}}_k$ are distinct, the eigenvalues of $Y_{k+1}$ are the solutions of

1 $$\begin{aligned} \sum _{j=0}^{n-1} i\frac{u_j+z}{u_j-z}\rho _j = i\frac{1+\eta }{1-\eta } \end{aligned}$$

where for $|z|=1$ the summands and the right-hand side are both real. The only randomness is contained in the values $\rho _j$, which have a Dirichlet joint distribution with all parameters equal to 1. To summarize, in order to get the evolution of $({\bar{Y}}_k)_{k \ge 1}$, we pick $(\rho _j)_{1 \le j \le n}$ from Dirichlet distribution, form the rational function given by the left-hand side of (1), and look at a particular level set to get the new eigenvalues.

This equation can be lifted to the real line. Let $(\lambda _j)_{j \in {\mathbb {Z}}}$ be the $(2 \pi n)$-periodic set of $\lambda \in {\mathbb {R}}$ such that $e^{i\lambda /n} \in \{u_1, \dots , u_n\}$, and extend the sequence $(\rho _j)_{1 \le j \le n}$ periodically with period *n* to all integer indices. With $z=e^{ix/n}$, the left-hand side of (1) can be written as

$$\begin{aligned} \lim _{\ell \rightarrow \infty } \sum _{j=-\ell }^{\ell } \frac{2n\rho _j}{\lambda _j-x}. \end{aligned}$$

and the level set of this at $i(1+\eta )/(1-\eta )$ gives the lifting of the eigenvalues at the next step. Recall that $\eta$ is a complex number of modulus 1, related to the angles of the complex reflections involved in the definition of the walk $(Y_k)_{k \ge 1}$. Notice now that essentially the only role of *n* in the above process is given by the joint distribution of the $\rho$-s. These are *n*-periodic and Dirichlet; clearly, as $n\rightarrow \infty$ they converge, after suitable renormalization, to independent exponential variables, giving naturally an infinite-dimensional Markov chain.

In the present article, we prove the existence of this Markov chain and deduce a new construction of the bead process. By replacing the exponential variables by gamma variables with general parameter, we construct a natural generalization of the bead process, indexed by a parameter $\beta > 0$. For $\beta = 2$, this process is the bead process itself, and then it is the limit of the eigenvalues of the GUE minors when the dimension goes to infinity. For $\beta = 1$, we show that we get the limit of the eigenvalues of the GOE minors, for $\beta = 4$, we get the limit of the eigenvalues of the GSE minors, and we generalize this result to all $\beta > 0$, by considering the Hermite $\beta$ corners, defined by Gorin and Shkolnikov [10], which can be informally viewed as the “eigenvalues of G$\beta$E minors”.

The sequel of the present paper is organized as follows.

In Sect. 2, we give the statement of the most important results of the article, and we refer to later propositions and theorems for the proofs. Our main results involve technicalities which are also explained in the sequel of the article.

In Sect. 3, we detail the above discussion on the random reflection walk, and we deduce a property of invariance for the law of the spectrum of a Haar-distributed unitary matrix, for the transition given by the Eq. (1). We generalize this property to circular beta ensembles for any $\beta > 0$.

In Sect. 4, we generalize the notion of Stieltjes transform to a class of infinite point measures on the real line for which the series given by the usual definition is not absolutely convergent.

In Sect. 5, we construct a family of Markov chains on a space of point measures, for which the transition mechanism is obtained by taking a level set of the Stieltjes transform defined in Sect. 4.

In Sect. 6, we show how the lifting of the unit circle on the real line defined above connects the results of Sect. 3 to those of Sect. 5.

In Sect. 7, we use some bound on the variance of the number of points of the circular beta ensembles in an arc, in order to take the limit of the results in Sect. 6, when the period of the point measure goes to infinity. We show a property of invariance enjoyed by the determinantal sine-kernel process and its generalizations for all $\beta > 0$, for the Markov chain defined in Sect. 5. From this Markov chain, we deduce the construction of a stationary point process on ${\mathbb {R}} \times {\mathbb {Z}}$, for which the points of a given line follow the distribution of the ${\text {Sine}}_{\beta }$ process introduced in Valkó and Virág [19].

In Sect. 8, we show, under some technical conditions, a property of continuity of the Markov chain with respect to the initial point measure and the weights.

From this result, and from a bound, proven in a companion paper [15] on the variance of the number of points of the Gaussian beta ensemble in intervals, we deduce in Sect. 9 that the generalized bead process constructed in Sect. 7 appears as a limit for the eigenvalues of the minors of Gaussian Ensembles for $\beta \in \{1,2,4\}$. The case $\beta = 2$ corresponds to the GUE, for which the convergence to the bead process defined by Boutillier [6] is already known from Adler et al. [1]. Combining our result with [1] then implies that our Markov chain has necessarily the same distribution as the bead process given in [6]. The case $\beta = 1$ gives the convergence of the renormalized eigenvalues of the GOE minors, and the case $\beta = 4$ gives the convergence of the renormalized eigenvalues of the GSE minors. For other values of $\beta$, we get a similar result of convergence for the renormalized points of the Hermite $\beta$ corner defined in [10].

## Statement of the main results

Our main result generalizes the bead process to any $\beta > 0$. We need the following definitions.

- Let ${\mathcal {L}}$ be the family of all the discrete subsets *L* of ${\mathbb {R}}$, unbounded from above and from below, and such that for $x \rightarrow \infty$, ${\text {Card}} (L \cap [0,x]) = O(x)$, and for fixed $a,b \in {\mathbb {R}}$, $x \rightarrow \infty$,
  $$\begin{aligned} {\text {Card}} (L \cap [0,x+a ]) - {\text {Card}} (L \cap [-x+b,0]) = O(x/\log ^2 x). \end{aligned}$$
  We endow the space ${\mathcal {L}}$ with the $\sigma$-algebra generated by the maps $L \mapsto {\text {Card}} (L \cap B)$ for all Borel sets $B \subset {\mathbb {R}}$. We will use $(\lambda _j)_{j \in {\mathbb {Z}}}$ as the unique increasing labeling of *L* so that $\lambda _{-1}<0\le \lambda _0$.
- Let $\Gamma$ be the family of doubly infinite sequences $(\gamma _j)_{j \in {\mathbb {Z}}}$ satisfying the following assumptions: for *k* going to infinity,
  $$\begin{aligned} \sum _{j=0}^k \gamma _j = ck + O(k/\log ^2 k) \; \; \mathrm { and } \; \; \sum _{j=0}^k \gamma _{-j} = ck + O(k/\log ^2 k), \end{aligned}$$
  where $c > 0$ is a constant. We endow $\Gamma$ with the $\sigma$-algebra generated by the coordinate maps $\gamma _j$, $j \in {\mathbb {Z}}$.

### Theorem 1

1. There exists a map ${\mathcal {D}}{:}\,{\mathcal {L}} \times \Gamma \times {\mathbb {R}}\rightarrow {\mathcal {L}}$, defined by
  $$\begin{aligned} {\mathcal {D}} ( L, (\gamma _j)_{j \in {\mathbb {Z}}}, h) = \left\{ z \in {\mathbb {R}}, \underset{c \rightarrow \infty }{\lim } \sum _{j \in {\mathbb {Z}}, \lambda _j \in L \cap [-c,c]} \frac{\gamma _{j}}{\lambda _j - z} = h \right\} . \end{aligned}$$
2. For any probability measure $\Pi$ on $\Gamma \times {\mathbb {R}}$ and any initial condition $X_0$ in ${\mathcal {L}}$, we can define a Markov chain by as follows: let $G_k$ be independent samples from $\Pi$, and set
  $$\begin{aligned} X_{k+1}={\mathcal {D}}(X_k,G_k), \qquad k\ge 0. \end{aligned}$$
3. Assume that under $\Pi$ the $((\beta /4)\gamma _j)_{j \in {\mathbb {Z}}}$ are independent Gamma random variables of shape parameter $\beta /2$, and *h* is a deterministic real number. Let $X_0$ be distributed as the $\hbox {Sine}_\beta$-process.
  Then $X_k$ is a stationary Markov chain. The $\beta$-**bead process** on ${\mathbb {R}} \times {\mathbb {N}}_0$ with level *h* is defined as the set
  $$\begin{aligned} \bigcup _{k \ge 0} (X_k \times \{k\} ). \end{aligned}$$
  The bead process on ${\mathbb {R}} \times {\mathbb {Z}}$ is the unique ${\mathbb {Z}}$-shift-invariant extension of the process on ${\mathbb {R}} \times {\mathbb {N}}_0$.

This theorem, which defines the $\beta$-bead process, is a consequence of results proven later in the article. The fact that the map ${\mathcal {D}}$ is well-defined is obtained in Sect. 5, as a consequence of a discussion on the existence and the regularity in *z* of the limit

$$\begin{aligned} \underset{c \rightarrow \infty }{\lim } \sum _{j \in {\mathbb {Z}}, \lambda _j \in L \cap [-c,c]} \frac{\gamma _{j}}{\lambda _j - z} \end{aligned}$$

which is made in Sect. 4, and which explains the technicalities involved in the definition of ${\mathcal {L}}$ and $\Gamma$. The invariance property of the distribution of the ${\text {Sine}}_{\beta }$ process for the Markov chain is proven in Theorem 13. An informal definition of the $\beta$-bead process can be given as follows: a $\beta$-bead process is a countable family of ${\text {Sine}}_{\beta }$ point processes, such that each of them is obtained from the previous one by putting independent ${\text {Gamma}}(\beta /2)$ distributed weights on the points, and by taking a given level set of the Stieltjes transform of the corresponding point measure. Notice that the point measure here is infinite and that the series defining the Stietjes transform is not absolutely convergent, which explains some of the technicalities involved in Theorem 1. Notice that the properties of the Stieltjes transform imply that two consecutive ${\text {Sine}}_{\beta }$ point processes involved in a $\beta$-bead process interlace with each other.

We prove Theorem 13 by finite approximation. There is general beta version of the eigenvalue evolution of the complex reflection random walk on unitary matrices. It corresponds exactly to taking $\gamma _i$ to be *n*-periodic with Dirichlet$(\beta /2,\ldots , \beta /2)$ distribution in Theorem 1. The periodic lifting of the circular beta ensemble points to the real line is a stationary distribution for the corresponding Markov chain, see Theorem 12. In Theorem 13, we show that as $n\rightarrow \infty$, this sequence of Markov chains converges, and we identify the $\beta$-bead process as its limit.

The bead process introduced by Boutillier is a determinantal process, with an explicit kernel. In the present article, we do not study the question of the correlations of the $\beta$-bead process: we expect that there are no simple general formulas, since the problem of finding explicit formulas for the correlations of the ${\text {Sine}}_{\beta }$ process is already unsolved for general $\beta > 0$. It may be possible to find expressions involving Pfaffians for the correlations of the $\beta$-bead process for $\beta = 1$ and $\beta = 4$.

Another natural question related to Theorem 1 is the following: is the ${\text {Sine}}_{\beta }$ distribution the unique invariant measure for the Markov chain associated to i.i.d. independent ${\text {Gamma}}(\beta /2)$ weights and independent level *h*? Strictly speaking, the answer is negative since we can multiply the points of the ${\text {Sine}}_{\beta }$ by a non-zero constant and still get an invariant distribution for the Markov chain. If we restrict the discussion to point processes on ${\mathbb {R}}$ for which the number of points in [0, *x*] and the number of points in $[-x,0]$ are equivalent to $x/2 \pi$ when *x* goes to infinity, we do not know if the invariant measure is unique. Symmetrically, one can also ask about the existence of other measures $\Pi$ on $\Gamma \times {\mathbb {R}}$ for which the ${\text {Sine}}_{\beta }$ distribution is invariant for the Markov chain.

The main property of the $\beta$-bead process is that it is the scaling limit of the Hermite $\beta$ corner process introduced by Gorin and Shkolnikov [10], see also [8, Proposition 4.3.2]. From Definition 1.1 of [10], we have, after taking $t = 2/\beta$:

### Definition 2

Let $n \ge 1$ be an integer. A Hermite $\beta$ corner process with *n* levels is a random set of reals $(\lambda ^{(k)}_j)_{1 \le j \le k \le n}$ subject to the interlacing conditions $\lambda ^{(k)}_j \le \lambda ^{(k-1)}_j \le \lambda ^{(k)}_{j+1}$ and such that the density of its probability distribution is given by

$$\begin{aligned}&\prod _{i< j} (\lambda _j^{(n)} - \lambda _i^{(n)}) \prod _{j = 1}^n e^{- \beta \lambda _j^{(n)}/4} \prod _{k = 1}^{n-1} \prod _{1 \le i < j \le k} (\lambda _j^{(k)} - \lambda _i^{(k)})^{2 - \beta } \\&\quad \times \prod _{a=1}^k\prod _{b=1}^{k+1} |\lambda _a^{(k)} - \lambda _b^{(k+1)}|^{(\beta /2)-1}. \end{aligned}$$

From [8, Proposition 4.3.2] (see Proposition 24) and the discussion above, we deduce that the successive levels $(\lambda ^{(k)})_{1 \le k \le n}$ of a Hermite $\beta$ corner process can be constructed as an inhomogeneous Markov chain whose transitions are explicitly written in term of level sets of Stieltjes transforms. Similar Markov chains and representations of eigenvalues of successive minors of random matrices in terms of zeros of meromorphic functions can be found in the literature: for more detail, we refer to articles by Gelfand and Naimark [9], Baryshnikov [2], Neretin [14], Okounkov and Reshetikhin [16].

This constructions implies that one can define a Hermite $\beta$ corner process $(\lambda ^{(k)}_j)_{1 \le j \le k}$ with infinitely many levels, in such a way that $(\lambda ^{(k)}_j)_{1 \le j \le k \le n}$ is a Hermite $\beta$ corner process with *n* levels for all $n \ge 1$. Moreover, for each $n \ge 1$ the *n*th level $\lambda ^{(n)}$ follows the Gaussian $\beta$ Ensemble, i.e. its joint density, with respect to the Lebesgue measure, is proportional to

$$\begin{aligned} e^{-\beta \sum _{k=1}^n \lambda ^{(n)}_k/4} \prod _{j < k} |\lambda ^{(n)}_j - \lambda ^{(n)}_k|^{\beta }. \end{aligned}$$

This, up to a change in the normalization, is shown in [10]. As we explain in more detail at the beginning of Sect. 9, the eigenvalues of the successive minors of a $n \times n$ matrix following the Gaussian Orthogonal Ensemble ($\beta = 1$), the Gaussian Unitary Ensemble ($\beta = 2$), or the Gaussian Symplectic Ensemble ($\beta = 4$), with a suitable normalization, have the same law as the successive levels of a Hermite $\beta$ corner process with *n* levels. If we take the minors of an infinite matrix, we get a Hermite $\beta$ corner with infinitely many levels. For this reason, for general $\beta > 0$, the Hermite $\beta$ corner process can be thought as the “eigenvalues of G$\beta$E minors”. In the present article, we prove that the $\beta$-bead process is the microscopic scaling limit of the Hermite $\beta$ corner process. From now, if $(\Xi _n)_{n \ge 1}$, $\Xi$ are locally finite measures on a measurable subspace of ${\mathbb {R}}^p$ for some $p \ge 1$ (e.g. ${\mathbb {R}}$ or ${\mathbb {R}} \times {\mathbb {N}}_0$), we will say that $\Xi _n$ converges to $\Xi$ locally weakly if and only if for all continuous functions from ${\mathbb {R}}^p$ to ${\mathbb {R}}$, compactly supported,

$$\begin{aligned} \int _{{\mathbb {R}}^p} \Phi \, d \Xi _n \underset{n \rightarrow \infty }{\longrightarrow } \int _{{\mathbb {R}}^p} \Phi \, d \Xi . \end{aligned}$$

The precise statement of our result is then the following:

### Theorem 3

Let us fix $E \in (-2,2)$. Let $(\lambda ^{(k)}_j)_{1 \le j \le k}$ be a Hermite $\beta$ corner process with infinitely many levels. For $n \ge 1$, we consider the point process on ${\mathbb {R}} \times {\mathbb {Z}}$ defined as the set

$$\begin{aligned} X_n := \left\{ ( \lambda ^{(n+k)}_j - E \sqrt{n}) \sqrt{ n(4-E^2)} , k), \; k \in {\mathbb {Z}} \cap [-n+1, \infty ) \right\} . \end{aligned}$$

Then, the sum of Dirac measures at the points of $X_n$ converges in law to the sum of Dirac measures at the points of a $\beta$-bead process on ${\mathbb {R}} \times {\mathbb {Z}}$, for the topology of locally weak convergence of measures on ${\mathbb {R}} \times {\mathbb {Z}}$, with a level *h* given by

$$\begin{aligned} h = - \frac{E}{\sqrt{4-E^2}}. \end{aligned}$$

If we restrict the point processes to ${\mathbb {R}} \times {\mathbb {N}}_0$, i.e. we take only points corresponding to $k \ge 0$, this statement corresponds to the first part of Theorem 26, the level *k* of the point process $X_n$ corresponding to the point process $\Xi _n^{(k)}$. Once the result is proven for point processes on ${\mathbb {R}} \times {\mathbb {N}}_0$, a suitable shift of *n* gives convergence of point processes on ${\mathbb {R}} \times ({\mathbb {Z}} \cap [-m,\infty ) )$ for any fixed $m \in {\mathbb {Z}}$, and then convergence of point processes on ${\mathbb {R}} \times {\mathbb {Z}}$ since the test functions in the locally weak convergence are compactly supported. The second part of Theorem 26 gives the following property of compatibility:

### Theorem 4

For fixed $h \in {\mathbb {R}}$, the $\beta$-bead process on ${\mathbb {R}} \times {\mathbb {Z}}$ for $\beta = 2$ and level *h* has the same law as the bead process in the sense of [6], with parameter

$$\begin{aligned} \gamma = - \frac{h}{\sqrt{1+h^2}}. \end{aligned}$$

## Random reflection chains on the unitary group

We start with a brief review of how multiplication by complex reflections changes eigenvalues. Let $U \in U(n)$ be a unitary matrix with distinct eigenvalues $u_1, \dots , u_n$, and let *v* be a unit vector. Let $a_1, \dots , a_n$ be the coefficients of *v* in a basis of unit eigenvectors of *U*, and let $\rho _j = |a_j|^2$ for $1 \le j \le n$: the law of $(\rho _1, \dots , \rho _n)$ does not depend on the choice of the eigenvector basis and the sum of these numbers is equal to 1.

If $\eta \not = 1$ is a complex number of modulus 1, the complex reflection with angle $\arg \eta$ and vector *v* corresponds to the unitary matrix $I+(\eta -1)vv^*$. If we multiply *U* by this reflection, we get a new matrix whose eigenvalues *u* satisfy

$$\begin{aligned} 0=\det \left( U(I+(\eta -1)vv^*)-u\right) , \end{aligned}$$

which can be rewritten as

$$\begin{aligned} 0=\det (U-u)\det \left( I+(\eta -1)Uvv^*(U-u)^{^{-1}}\right) \end{aligned}$$

when *u* is not an eigenvalue of *U*. Now, the second argument is *I* plus a rank-1 matrix, so its determinant equals 1 plus the trace of the rank-1 matrix. Thus the equation above reduces to

$$\begin{aligned} 0=1+ (\eta -1)\mathrm{tr}(Uvv^*(U-u)^{-1}) = 1 + (\eta -1)v^*((U-u)^{-1}U)v. \end{aligned}$$

Expanding *U* in the basis of its eigenvectors and eigenvalues $u_j$, we get

$$\begin{aligned} 1=(1 - \eta ) \sum _{j=1}^n \rho _j \frac{u_j}{u_j-u} \end{aligned}$$

or, after a transformation,

2 $$\begin{aligned} \sum _{j=1}^n i \rho _j \frac{u_j+u}{u_j-u}=i\frac{1+\eta }{1-\eta }. \end{aligned}$$

As *u* moves counterclockwise on the unit circle, and on each arc between two consecutive poles, the left-hand side of (2) is continuous and strictly increasing from $-\infty$ to $\infty$. Hence, the matrix $U(I+(\eta -1)vv^*)$ has exactly one eigenvalue in each arc between eigenvalues of *U*: in other words, the eigenvalues of $U(I+(\eta -1)vv^*)$ strictly interlace between those of *U*, and are given by the solutions *u* of the Eq. (2).

Consider the product of the unit sphere in ${\mathbb {C}}^n$ and ${\mathbb {R}}$, and a distribution $\pi$ on this space which is invariant under permutations of the *n* coordinates of the sphere, and by multiplication of each of these coordinates by complex numbers of modulus one. For such a distribution, we can associate a Markov chain on unitary matrices as follows. Given $U_0,\ldots , U_k$, we pick a sample $((a_1, \dots , a_n),h)$ from $\pi$ independently from the past. Then, $U_{k+1}$ is defined as the product of $U_{k}$ by the reflection with parameter $\eta$ so that $h=i\frac{\eta +1}{\eta -1}$, and vector $v=\sum a_j \varphi _j$, where $(\varphi _j)_{1 \le j \le n}$ are unit eigenvectors of $U_k$ (from the assumption made on $\pi$, the law of *v* does not depend on the choice of the phases of the eigenvectors $(\varphi _j)_{1 \le j \le n}$).

From the discussion above, it is straightforward that if $V_k$ is the spectrum of $U_k$, then $(V_k)_{k \ge 0}$ forms a Markov process as well; its distribution depends on the coefficients $a_j$ only through $\rho _j = |a_j|^2$. The transition is given as follows: given $V_j$, $(\rho _j)_{1 \le j \le n}$ and *h*, $V_{j+1}$ is formed by the *n* solutions of (2).

When *a* is uniform on the unit complex sphere of ${\mathbb {C}}^n$, and *h* is independent of *a*, then $(\rho _j)_{1 \le j \le n}$ has Dirichlet$(1,\ldots ,1)$ distribution, and the corresponding reflection is independent of $U_k$. Thus the Markov chain reduces to a random walk: $U_j=U_0R_1\ldots R_k$, where the reflections $(R_k)_{k \ge 1}$ are independent.

It is immediate that the Haar measure on *U*(*n*) is invariant for this random walk. One deduces that if $(\rho _j)_{1 \le j \le n}$ follows a Dirichlet distribution with all parameters equal to 1, if *h* (and then $\eta$) is independent of $(\rho _j)_{1 \le j \le n}$, if the points of $V_0$ follow the distribution of the eigenvalues of the CUE in dimension *n*, and if $(V_k)_{k \ge 0}$ is the Markov chain described above, then the law of $V_k$ does not depends of *k*: the CUE distribution is invariant for this Markov chain.

This invariance property can be generalized to other distributions $\pi$.

Indeed, as in Simon [18], one can associate to the point measure $\sigma := \sum _{j=1}^n \rho _j \delta _{u_j}$ a so-called *Schur function* $f_{\sigma }$, which is rational, and which can be defined by the equation:

3 $$\begin{aligned} \int _{{\mathbb {U}}} i \frac{v+u}{v-u} d \sigma (v) =i\frac{1+u f_{\sigma }(u)}{1-u f_{\sigma } (u)}. \end{aligned}$$

Moreover, as explained in [18], by Geronimus theorem, we also have

$$\begin{aligned} f_{\sigma }(u) = R_{\alpha _0} \circ M_u \circ R_{\alpha _1} \circ M_u \circ R_{\alpha _2} \circ \cdots \circ R_{\alpha _{n-2}} \circ M_u (\alpha _{n-1}), \end{aligned}$$

where $M_u$ denotes the multiplication by *u*, the $(\alpha _j)_{0 \le j \le n-1}$ are the Verblunsky coefficients associated to the orthogonal polynomials with respect to the measure $\sigma$, and for all $\alpha \in {\mathbb {D}}$, $R_{\alpha }$ is the Möbius transformation given by

$$\begin{aligned} R_{\alpha } (z) = \frac{\alpha + z}{1 + {\overline{\alpha }} z}. \end{aligned}$$

By (3), we see that (2) is satisfied if and only if $u f_{\sigma }(u) = \eta$, or equivalently,

4 $$\begin{aligned} M_{\eta ^{-1}} \circ M_{u} \circ R_{\alpha _0} \circ M_u \circ R_{\alpha _1} \circ \cdots \circ M_u (\alpha _{n-1}) = 1. \end{aligned}$$

Now, $M_{\eta ^{-1}}$ and $M_{u}$ commute and for $\alpha \in {\mathbb {D}}$, $M_{\eta ^{-1}} \circ R_{\alpha } = R_{\alpha \eta ^{-1}} \circ M_{\eta ^{-1}}$. One deduces that (4) is equivalent to

$$\begin{aligned} M_u \circ R_{\alpha _0 \eta ^{-1}} \circ M_u \circ R_{\alpha _1 \eta ^{-1}} \circ \cdots \circ M_u (\alpha _{n-1} \eta ^{-1}) = 1, \end{aligned}$$

i.e. $u f_{\tau } (u) = 1$, where $\tau$ is the finitely supported probability measure whose Verblunsky coefficients are $(\alpha _0 \eta ^{-1}, \dots , \alpha _{n-1}\eta ^{-1})$. Now, by the general construction of the Schur functions, the equation $u f_{\tau } (u) = 1$ is satisfied if and only if *u* is a point of the support of $\tau$: in other words, this support is the set of solutions of (2). We deduce that if the distribution $\pi$ and the law of $\{u_1, \dots , u_n\}$ are chosen in such a way that $(\alpha _0 \eta ^{-1}, \dots , \alpha _{n-1} \eta ^{-1})$ has the same law as $(\alpha _0, \dots , \alpha _{n-1})$, then the law of $\{u_1, \dots , u_n\}$ is invariant for the Markov chain described above. The precise statement is the following:

### Proposition 5

Let $\pi$ be a probability distribution on the product of the unit sphere of ${\mathbb {C}}^n$ and ${\mathbb {R}}$, under which the first component $(a_1, \dots , a_n)$ is independent of the second $h = i (1 + \eta )/(1- \eta )$. We suppose that the law of $(a_1, \dots , a_n)$ is invariant by permutation of the coordinates, and by their pointwise multiplication by complex numbers of modulus 1. Let ${\mathbb {P}}$ be a probability measure of the sets of *n* points $\{u_1, \dots , u_n\}$, such that under the product measure ${\mathbb {P}} \otimes \pi$, the sequence $(\alpha _0, \dots , \alpha _{n-1})$ of Verblunsky coefficients associated to the measure

$$\begin{aligned} \sigma = \sum _{1 \le j \le n} \rho _j \delta _{u_j} = \sum _{1 \le j \le n} |a_j|^2 \delta _{u_j}. \end{aligned}$$

has a law which is invariant by multiplication by complex numbers of modulus 1. Then, the measure ${\mathbb {P}}$ is invariant for the Markov chain associated to $\pi$: more precisely, under ${\mathbb {P}} \otimes \pi$, the law of the set of solutions of (2) is equal to ${\mathbb {P}}$.

It is not obvious to find explicitly some measures ${\mathbb {P}}$ and $\pi$ under which the law of the Verblunsky coefficients is invariant by rotation. An important example is obtained by considering the so-called *circular beta ensembles*. These ensembles are constructed as follows: for some parameter $\beta > 0$, one defines a probability measure ${\mathbb {P}}_{n,\beta }$ on the sets of *n* points on the unit circle, such that the corresponding *n*-point correlation function $r_{n,\beta }$ is given, for $z_1, \dots z_n \in {\mathbb {U}}$, by

$$\begin{aligned} r_{n, \beta } (z_1, \dots , z_n) = C_{n, \beta } \, \prod _{1 \le j < k \le n} |z_j - z_k|^{\beta }, \end{aligned}$$

where $C_{n, \beta } > 0$ is a normalization constant. Note that, for $\beta = 2$, one obtains the distribution of the spectrum of a random $n \times n$ unitary matrix following the Haar measure. Now, let $\pi _{n, \beta }$ be any distribution on the product of the unit sphere of ${\mathbb {C}}^n$ and ${\mathbb {R}}$, such that with the notation above, *h* is independent of $(\rho _0, \dots , \rho _{n-1})$, which has a Dirichlet distribution with all parameters equal to $\beta /2$. Then, under ${\mathbb {P}}_{n, \beta } \otimes \pi _{n, \beta }$, the distribution of the Verblunsky coefficients $(\alpha _0, \alpha _1, \dots , \alpha _{n-1})$ has been computed in Killip and Nenciu [12]. One obtains the following:

- The coefficients $\alpha _0, \alpha _1, \dots \alpha _{n-1}$ are independent random variables.
- The coefficient $\alpha _{n-1}$ is uniform on the unit circle.
- For $j \in \{0, 1, \dots , n-2\}$, the law of $\alpha _j$ has density $(\beta /2)(n-j-1) (1-|\alpha _j|^2)^{(\beta /2)(n-j-1) - 1}$ with respect to the uniform probability measure on the unit disc: note that $|\alpha _j|^2$ is then a beta variable of parameters 1 and $\beta (n-j-1)/2$.

Therefore, the law of $(\alpha _0, \alpha _1, \dots , \alpha _{n-1})$ is invariant by rotation, and one deduces the following result:

### Proposition 6

The law of the circular beta ensemble is an invariant measure for the Markov chain associated to $\pi _{n, \beta }$. More precisely, under ${\mathbb {P}}_{n, \beta } \otimes \pi _{n, \beta }$, the set of solutions of (2) follows the distribution ${\mathbb {P}}_{n, \beta }$.

In the next sections, we will take a limit when *n* goes to infinity. For this purpose, we need to consider point processes on the real line instead of the unit circle, and to find an equivalent of the Eq. (2) in this setting.

## Stieltjes transform for point measures

Let $\Lambda$ be a $\sigma$-finite point measure on ${\mathbb {R}}$, which can be written as follows:

$$\begin{aligned} \Lambda = \sum _{\lambda \in L} \gamma _{\lambda } \delta _{\lambda }, \end{aligned}$$

where *L* is a discrete subset of the real line, $\gamma _{\lambda } > 0$ for all $\lambda \in L$, and $\delta _{\lambda }$ is the Dirac measure at $\lambda$. The usual definition of the Stieltjes transform applied to $\Lambda$ gives, for $z \in {\mathbb {C}} {\backslash }\{L\}$:

5 $$\begin{aligned} S_{\Lambda } (z) = \sum _{\lambda \in L} \frac{\gamma _{\lambda }}{\lambda - z}. \end{aligned}$$

If the set *L* is finite, then $S_{\Lambda }(z)$ is well-defined as a rational function. If *L* is infinite and if the right-hand side of (5) is absolutely convergent, then this equation is still meaningful. The following result implies that under some technical assumptions, one can define $S_{\Lambda }$ even if (5) does not apply directly:

### Theorem 7

Assume that for all $a, b \in {\mathbb {R}}$, $\Lambda [0,x+a] - \Lambda [-x+b,0] = O(x/\log ^2 x)$ as $x\rightarrow \infty$. Then, for all $z \in {\mathbb {C}} {\backslash }\{L\}$, there exists $S_{\Lambda } (z) \in {\mathbb {C}}$ such that

$$\begin{aligned} \sum _{\lambda \in L \cap [-c, c]} \frac{\gamma _{\lambda }}{\lambda - z} \underset{c \rightarrow \infty }{\longrightarrow } S_{\Lambda }(z). \end{aligned}$$

The function $S_{\Lambda }$ defined in this way is meromorphic, with simple poles at the elements of *L*, and the residue at $\lambda \in L$ is equal to $- \gamma _{\lambda }$. The derivative of $S_{\Lambda }$ is given by

6 $$\begin{aligned} S'_{\Lambda }(z) = \sum _{\lambda \in L} \frac{\gamma _{\lambda }}{(\lambda - z)^2}, \end{aligned}$$

where the convergence of the series is uniform on compact sets of ${\mathbb {C}} {\backslash }\{L\}$. For all pairs $\{\lambda _1, \lambda _2\}$ of consecutive points in *L*, with $\lambda _1 < \lambda _2$, the function $S_{\Lambda }$ is a strictly increasing bijection from $(\lambda _1, \lambda _2)$ to ${\mathbb {R}}$. Moreover, we have the following translation invariance: if $y\in {\mathbb {R}}$ and $\Lambda$ satisfies the conditions above, then so does its translation $\Lambda +y$, and one has

$$\begin{aligned} S_{\Lambda + y}(z + y) = S_{\Lambda } (z) \end{aligned}$$

for all $$z \in {\mathbb {C}} {\backslash }\{L\}$$.

### Remark 8

The bound $x/\log ^2 x$ is not optimal (any increasing function which is negligible with respect to *x* and integrable against $dx/x^2$ at infinity would work). However, it will be sufficient for our purpose.

### Proof

Let $c_0 > 1$, and $z \in {\mathbb {C}}$ such that $|z| \le c_0/2$. For $c > c_0$, we have:

$$\begin{aligned} \sum _{\lambda \in L \cap ([-c,-c_0] \cup [c_0,c] )} \frac{\gamma _{\lambda }}{\lambda - z }&= \sum _{\lambda \in L \cap [c_0,c]} \gamma _{\lambda } \int _{\lambda }^{\infty } \frac{d \mu }{(\mu -z)^2} \\&\quad -\, \sum _{\lambda \in L \cap [-c,-c_0]} \gamma _{\lambda } \int _{-\infty }^{\lambda } \frac{d \mu }{(\mu -z)^2} \\&= \!\int _{c_0}^{\infty } \frac{ \Lambda ([c_0, c {\wedge } \mu ])}{(\mu {-}z)^2} \, d\mu {-} \int _{-\infty }^{-c_0} \frac{ \Lambda ([(-c) \vee \mu , {-} c_0])}{(\mu {-}z)^2} \, d\mu \\&= \int _{c_0}^{\infty } \left( \frac{ \Lambda ([c_0, c \wedge \mu ])}{(\mu -z)^2} - \frac{ \Lambda ([-(c \wedge \mu ), - c_0])}{(\mu +z)^2} \right) \, d \mu \\&= \int _{c_0}^{\infty } \frac{ \Lambda ([c_0, c \wedge \mu ]) - \Lambda ([-(c \wedge \mu ), - c_0])}{\mu ^2} \, d \mu \\&\quad +\, \int _{c_0}^{\infty } \left( \frac{ (2 z \mu - z^2) (\Lambda ([c_0, c \wedge \mu ]) )}{\mu ^2 (\mu -z)^2} \right. \\&\quad \left. +\, \frac{(2 z \mu + z^2) (\Lambda ([-(c \wedge \mu ), -c_0]) )}{\mu ^2 (\mu +z)^2} \right) \, d\mu . \end{aligned}$$

Let *F* be an increasing function from ${\mathbb {R}}_+$ to ${\mathbb {R}}_+^* := (0,\infty )$, such that *F*(*x*) is equivalent to $x/\log ^2 x$ when *x* goes to infinity. By assumption, there exists $C > 0$ such that for all $x \ge 0$, $|\Lambda ([0, x]) - \Lambda ([-x, 0])| \le C F(x)$, and then, for all $\mu \ge c_0$,

$$\begin{aligned}&|\Lambda ([c_0, c \wedge \mu ]) - \Lambda ([-(c \wedge \mu ), - c_0])|\\&\quad \le C F(c \wedge \mu ) + \Lambda ([-c_0, c_0]) \le \left( C + \frac{ \Lambda ([-c_0, c_0]) }{F(0)} \right) \, F(\mu ). \end{aligned}$$

Since $\mu \mapsto F(\mu )/\mu ^2$ is integrable at infinity, one obtains, by dominated convergence,

$$\begin{aligned}&\int _{c_0}^{\infty } \frac{ \Lambda ([c_0, c \wedge \mu ]) - \Lambda ([-(c \wedge \mu ), - c_0])}{\mu ^2} \, d \mu \\&\quad \underset{c \rightarrow \infty }{\longrightarrow } \int _{c_0}^{\infty } \frac{ \Lambda ([c_0, \mu ]) - \Lambda ([-\mu , - c_0])}{\mu ^2} \, d \mu , \end{aligned}$$

where the limiting integral is absolutely convergent. Similarly, there exist $C', C'' > 0$ such that for all $x \ge 0$, $|\Lambda ([0,x+1]) - \Lambda ([-x,0])| \le C' F(x)$ and $|\Lambda ([0,x]) - \Lambda ([-x-1,0])| \le C'' F(x)$, which implies that

$$\begin{aligned} \Lambda ((x,x+1]) + \Lambda ([-x-1,-x))&\le |\Lambda ([0,x+1]) - \Lambda ([-x,0])| \\&\quad +\, |\Lambda ([0,x]) - \Lambda ([-x-1,0])| \\&\le (C'+C'') F(x). \end{aligned}$$

Hence, for all integers $n \ge 1$,

$$\begin{aligned} \Lambda ([-n,n])&= \Lambda (\{0\}) + \sum _{k=0}^{n-1} (\Lambda ((k,k+1]) + \Lambda ([-k-1,-k)) \\&\le \Lambda (\{0\}) + (C' + C'') \, \sum _{k=0}^{n-1} F(k) \le K n F(n-1) \end{aligned}$$

where $K > 0$ is a constant, and then for all $x \ge 0$, $\Lambda ([-x,x]) \le K (1+x) F(x)$, which implies that for $\mu \ge c_0$, $\Lambda ([-(c \wedge \mu ), -c_0]) \le K (1+ \mu ) F(\mu )$ and $\Lambda ([c_0, c \wedge \mu ]) \le K(1+ \mu ) F(\mu )$. Moreover, since $|z| \le c_0/2 \le \mu /2$, one has $|\mu - z| \ge \mu /2$, $|\mu + z| \ge \mu /2$ and

7 $$\begin{aligned} \left| \frac{ (2 z \mu - z^2)}{\mu ^2 (\mu -z)^2} \right| + \left| \frac{ (2 z \mu + z^2)}{\mu ^2 (\mu +z)^2} \right| \le 2 \, \frac{ 2.5 |z| \mu }{ \mu ^2 (\mu /2)^2} = 20 |z| / \mu ^3 \le 10 \, c_0/\mu ^3 \end{aligned}$$

Since $\mu \mapsto (1+ \mu ) F(\mu ) / \mu ^3$ is integrable at infinity, one can again apply dominated convergence and obtain that

$$\begin{aligned} \int _{c_0}^{\infty } \left( \frac{ (2 z \mu - z^2) (\Lambda ([c_0, c \wedge \mu ]) )}{\mu ^2 (\mu -z)^2} + \frac{(2 z \mu + z^2) (\Lambda ([-(c \wedge \mu ), -c_0]) )}{\mu ^2 (\mu +z)^2} \right) \, d\mu \end{aligned}$$

tends to

$$\begin{aligned} \int _{c_0}^{\infty } \left( \frac{ (2 z \mu - z^2) (\Lambda ([c_0, \mu ]) )}{\mu ^2 (\mu -z)^2} + \frac{(2 z \mu + z^2) (\Lambda ([-\mu , -c_0]) )}{\mu ^2 (\mu +z)^2} \right) \, d\mu \end{aligned}$$

when *c* goes to infinity. Therefore,

$$\begin{aligned}&\sum _{\lambda \in L \cap ([-c,-c_0] \cup [c_0,c] )} \frac{\gamma _{\lambda }}{\lambda - z } \underset{c \rightarrow \infty }{\longrightarrow } \int _{c_0}^{\infty } \frac{ \Lambda ([c_0, \mu ]) - \Lambda ([-\mu , - c_0])}{\mu ^2} \, d \mu \\&\quad +\, \int _{c_0}^{\infty } \left( \frac{ (2 z \mu - z^2) (\Lambda ([c_0, \mu ]) )}{\mu ^2 (\mu -z)^2} + \frac{(2 z \mu + z^2) (\Lambda ([-\mu , -c_0]) )}{\mu ^2 (\mu +z)^2} \right) \, d\mu , \end{aligned}$$

which proves the existence of the limit defining $S_{\Lambda } (z)$: explicitly, for $z \in {\mathbb {C}} {\backslash }\{L\}$ and for any $c_0 > 2|z| \vee 1$,

8 $$\begin{aligned} S_{\Lambda }(z)&= \sum _{\lambda \in L \cap (-c_0,c_0)} \frac{\gamma _{\lambda }}{\lambda - z } + \int _{c_0}^{\infty } \frac{ \Lambda ([c_0, \mu ]) - \Lambda ([-\mu , - c_0])}{\mu ^2} \, d \mu \nonumber \\&\quad +\, \int _{c_0}^{\infty } \left( \frac{ (2 z \mu - z^2) (\Lambda ([c_0, \mu ]) )}{\mu ^2 (\mu -z)^2} + \frac{(2 z \mu + z^2) (\Lambda ([-\mu , -c_0]) )}{\mu ^2 (\mu +z)^2} \right) \, d\mu . \end{aligned}$$

For fixed $c_0 > 0$, the first term of (8) is a rational function of *z*, the second term of (8) does not depend on *z*, and by dominated convergence, the third term can be differentiated in the integral if we restrict *z* to the set $\{|z| < c_0/2\}$. Hence, the restriction of $S_{\Lambda }$ to the set $\{|z| < c_0/2\}$ is meromorphic, with simple poles at points $\lambda \in L \cap (-c_0/2, c_0/2)$. Since $c_0$ can be taken arbitrarily large, $S_{\Lambda }$ is in fact meromorphic on ${\mathbb {C}}$, with poles $\lambda \in L$, the pole $\lambda$ having residue $- \gamma _{\lambda }$. The derivative $S'_{\Lambda }(z)$ is given, for any $c_0 > 2 |z| \vee 1$, by:

$$\begin{aligned} S'_{\Lambda } (z)&= \sum _{\lambda \in L \cap (-c_0,c_0)} \frac{\gamma _{\lambda }}{(\lambda - z)^2 } + 2 \, \int _{c_0}^{\infty } \left( \frac{ (\Lambda ([c_0, \mu ]) )}{ (\mu -z)^3} + \frac{ (\Lambda ([-\mu , -c_0]) )}{ (\mu +z)^3} \right) \, d\mu . \\&= \sum _{\lambda \in L \cap (-c_0,c_0)} \frac{\gamma _{\lambda }}{(\lambda - z)^2 } + \int _{c_0}^{\infty } \, \left( \sum _{\lambda \in L \cap [c_0, \mu ]} \gamma _{\lambda } \right) \, \frac{2 \, d \mu }{(\mu -z)^3} \\&\quad + \int _{c_0}^{\infty } \, \left( \sum _{\lambda \in L \cap [-\mu , -c_0]} \gamma _{\lambda } \right) \, \frac{2 \, d \mu }{(\mu +z)^3} \\&= \sum _{\lambda \in L \cap (-c_0,c_0)} \frac{\gamma _{\lambda }}{(\lambda - z)^2 } + \sum _{\lambda \in L \cap [c_0, \infty )} \gamma _{\lambda } \int _{\lambda }^{\infty } \, \frac{2 \, d \mu }{(\mu -z)^3} \\&\quad + \sum _{\lambda \in L \cap (-\infty , c_0]} \gamma _{\lambda } \int _{-\lambda }^{\infty } \, \frac{2 \, d \mu }{(\mu +z)^3}, \end{aligned}$$

which implies (6). Note that the implicit use of Fubini theorem in this computation is correct since all the sums and integral involved are absolutely convergent.

Now, let ${\mathcal {K}}$ be a compact set of ${\mathbb {C}} {\backslash }L$, let $d > 0$ be the distance between ${\mathcal {K}}$ and *L*, and let $A > 0$ be the maximal modulus of the elements of ${\mathcal {K}}$. For all $z \in {\mathcal {K}}$ and $\lambda \in L$, one has, for $|\lambda | \le 2A+1$,

$$\begin{aligned} \left| \frac{\gamma _{\lambda } }{(\lambda - z)^2} \right| \le \frac{\gamma _{\lambda }}{d^2} \le \frac{1+ (2A+1)^2}{d^2} \cdot \frac{\gamma _{\lambda }}{1 + \lambda ^2} \end{aligned}$$

and for $$|\lambda | \ge 2A+1$$,

$$\begin{aligned} \left| \frac{\gamma _{\lambda } }{(\lambda - z)^2} \right| \le \frac{\gamma _{\lambda } }{ (|\lambda | - A)^2} \le \frac{4 \gamma _{\lambda } }{ \lambda ^2} \le \frac{8 \gamma _{\lambda } }{1 + \lambda ^2}. \end{aligned}$$

Hence, in order to prove the uniform convergence of (6) on compact sets, it is sufficient to check that

$$\begin{aligned} \sum _{\lambda \in L} \frac{\gamma _{\lambda } }{1 + \lambda ^2} < \infty , \end{aligned}$$

but this convergence is directly implied by the absolute convergence of the right-hand side of (6) for any single value of $z \in {\mathbb {C}} {\backslash }L$ (say, $z = i$), which has been proven before.

The formula (6) applied to $z \in {\mathbb {R}}$ implies immediately that for all pairs $\{\lambda _1, \lambda _2\}$ of consecutive points in *L*, with $\lambda _1 < \lambda _2$, the function $S_{\Lambda }$ is strictly increasing on the interval $(\lambda _1, \lambda _2)$. Moreover, one has for $\lambda \in \{\lambda _1, \lambda _2\}$ and $z \rightarrow \lambda$, $S_{\lambda }(z) \sim \gamma _{\lambda }/(\lambda - z)$, which implies that $S_{\Lambda }(z) \rightarrow -\infty$ for $z \rightarrow \lambda _1$ and $z >\lambda _1$, and $S_{\Lambda }(z) \rightarrow +\infty$ for $z \rightarrow \lambda _2$ and $z <\lambda _2$. We deduce that $S_{\Lambda }$ is a bijection from $(\lambda _1, \lambda _2)$ to ${\mathbb {R}}$.

It only remains to show the invariance by translation. If we fix $y \in {\mathbb {R}}$, then for all $a, b \in {\mathbb {R}}$, and for $x \ge 0$ large enough,

$$\begin{aligned}&(\Lambda +y)([0,x+a]) - (\Lambda + y)([-x+b,0]) \\&\quad = \Lambda ([-y, x+a-y])- \Lambda ([-x + b-y, -y]) \\&\quad = \Lambda ([0, x+a-y])- \Lambda ([-x + b-y, 0]) + O(\Lambda ([-|y|,|y|])) \\&\quad = O(x/\log ^2 x) + O(1) = O(x/\log ^2 x), \end{aligned}$$

and the assumptions of Theorem 7 are satisfied. One has

$$\begin{aligned} \Lambda + y = \sum _{\lambda \in L} \gamma _{\lambda } \delta _{\lambda + y}, \end{aligned}$$

and then for all $z \in {\mathbb {C}} {\backslash }L$,

$$\begin{aligned} S_{\Lambda + y} (z+y) = \lim _{c \rightarrow \infty } \, \sum _{\lambda \in (L+y) \cap [-c,c]} \, \frac{ \gamma _{\lambda -y} }{z + y- \lambda } = \lim _{c \rightarrow \infty } \, \sum _{\lambda \in L \cap [-c-y,c-y]} \, \frac{ \gamma _{\lambda } }{z - \lambda }, \end{aligned}$$

which is equal to $S_{\Lambda }(z)$, provided that we check that

$$\begin{aligned} \sum _{\lambda \in L \cap [-c-y,c-y]} \, \frac{ \gamma _{\lambda } }{z - \lambda } - \sum _{\lambda \in L \cap [-c,c]} \, \frac{ \gamma _{\lambda } }{z - \lambda } \underset{c \rightarrow \infty }{\longrightarrow } 0, \end{aligned}$$

which is implied by

9 $$\begin{aligned} \sum _{\lambda \in L \cap [-c-|y|,-c + |y|] } \frac{ \gamma _{\lambda } }{|z - \lambda | } + \sum _{\lambda \in L \cap [c-|y|,c + |y|] } \frac{ \gamma _{\lambda } }{|z - \lambda | } \underset{c \rightarrow \infty }{\longrightarrow } 0. \end{aligned}$$

Now, for $c > |y|+|z| + 1$, the left-hand side of (9) is smaller than or equal to

$$\begin{aligned}&\frac{\Lambda ([-c-|y|, -c + |y|]) + \Lambda ([c-|y|, c+ |y|])}{c -|z| {-} |y|} \\&\quad \le \frac{|\Lambda ([0,c+|y|]) {-} \Lambda ([-c+|y|+1, 0])| {+} |\Lambda ([0,c-|y|-1]) - \Lambda ([-c-|y|,0])|}{ c-|y|-|z|}{=} O(1/\log ^2 c), \end{aligned}$$

for *c* tending to infinity. $\square$

The assumption of Theorem 7 depends on the fact that the measure $\Lambda$ is not too far from being symmetric with respect to a given point on the real line. The next proposition expresses this assumption in terms of the support *L* of $\Lambda$ and the weights $(\gamma _{\lambda })_{\lambda \in L}$. The following result gives a sufficient condition for Theorem 7:

### Proposition 9

Consider the measure

$$\begin{aligned} \Lambda = \sum _{j\in {\mathbb {Z}}} {\gamma _j} \delta _{\lambda _j} \end{aligned}$$

where $(\lambda _j)_{j \in {\mathbb {Z}}}$ is strictly increasing and neither bounded from above nor from below, and $\gamma _j > 0$. Let *L* be the set $\{\lambda _j, j \in {\mathbb {Z}}\}$. Assume that for some $c > 0$,

$$\begin{aligned} \sum _{j=0}^k \gamma _j = ck + O(k/\log ^2 k) \; \; \mathrm { and } \; \; \sum _{j=0}^k \gamma _{-j} = ck + O(k/\log ^2 k), \end{aligned}$$

when $k\rightarrow \infty$. If for $x\rightarrow \infty$ one has ${\text {Card}} (L \cap [0,x] ) = O(x)$ and for all $a, b \in {\mathbb {R}}$, ${\text {Card}} (L \cap [0,x+a] ) - {\text {Card}} (L \cap [-x+b,0] ) = O(x/\log ^2 x)$, then the assumptions of Theorem 7 are satisfied.

### Proof

For $y \in {\mathbb {R}}$, let *N*(*y*) (resp. $N(y-)$) be the largest index *j* such that $\lambda _j \le y$ (resp. $\lambda _j < y$). One has, for $a, b \in {\mathbb {R}}$ and for *x* large enough,

$$\begin{aligned} \Lambda ([0,x+a]) = \sum _{j=N(0-) +1}^{N(x+a)} \gamma _j \; \; \mathrm { and } \; \; \Lambda ([-x+b,0]) = \sum _{j=N((-x+b)-)}^{N(0)} \gamma _j, \end{aligned}$$

which implies that for $x \rightarrow \infty$ and then $N(x+a) \rightarrow \infty$, $N((-x+b)-) \rightarrow - \infty$:

$$\begin{aligned}&\Lambda ([0,x+a]) - \Lambda ([-x+b,0]) = c ( N(x+a) - |N((-x+b)-)|) \\&\qquad \quad + O\left( \frac{N(x+a)}{ \log ^2 (N(x+a))} + \frac{|N((-x+b)-)|}{ \log ^2 |N((-x+b)-)|} \right) . \end{aligned}$$

Now, we have the following estimates:

$$\begin{aligned}&N(x+a) = {\text {Card}} (L \cap [0,x+a] ) + O(1) = O(x+a)\\&\quad +\,O(1) = O(x), \frac{N(x+a)}{ \log ^2 (N(x+a))} = O(x/\log ^2 x), \\&N(x+a) - |N((-x+b)-)| = {\text {Card}} (L \cap [0,x+a] ) - {\text {Card}} (L \cap [-x+b,0] ) \\&\quad +\,O(1) = O(x/\log ^2 x), |N((-x+b)-)| \le N(x+a) + |N(x+a) \\&\quad -\, |N((-x+b)-)| | \le O(x) + O(x/\log ^2 x) = O(x) \end{aligned}$$

and

$$\begin{aligned} \frac{|N((-x+b)-)|}{ \log ^2 |N((-x+b)-)|}= O(x/\log ^2 x). \end{aligned}$$

Putting all together gives:

$$\begin{aligned} \Lambda ([0,x+a]) - \Lambda ([-x+b,0]) = O(x/\log ^2 x) \end{aligned}$$

and then the assumptions of Theorem 7 are satisfied. $\square$

As written in the statement of Theorem 7, the function $S_{\Lambda }$ induces a bijection between each interval $(\lambda _1,\lambda _2)$, $\lambda _1$ and $\lambda _2$ being two consecutive points of *L*, and the real line. It is then natural to study the inverse of this bijection, which should map each element of ${\mathbb {R}}$ to a set of points interlacing with *L*. The precise statement we obtain is the following:

### Proposition 10

Let $\Lambda$ be a measure, whose support *L* is neither bounded from above nor from below, and satisfying the assumptions of Theorem 7. Then, for all $h \in {\mathbb {R}}$, the set $S^{-1}_{\Lambda } (h)$ of $z \in {\mathbb {C}} \backslash \{L\}$ such that $S_{\Lambda }(z) = h$ is included in ${\mathbb {R}}$, and interlaces with *L*, i.e. it contains exactly one point in each open interval between two consecutive points of *L*. Moreover, if $\Lambda$ satisfies the assumptions of Proposition 9, then it is also the case for the set $L' := S^{-1}_{\Lambda } (h)$, i.e. for *x* going to infinity, one has ${\text {Card}} (L' \cap [0,x] ) = O(x)$ and for all $a, b \in {\mathbb {R}}$, ${\text {Card}} (L' \cap [0,x+a] ) - {\text {Card}} (L' \cap [-x+b,0] ) = O(x/\log ^2 x)$.

### Proof

The interlacing property of points of $S^{-1}_{\Lambda }(h) \cap {\mathbb {R}}$ comes from the discussion above, so the first part of the proposition is proven if we check that $S_{\Lambda }(z) \notin {\mathbb {R}}$ if $z \notin {\mathbb {R}}$. Now, for all $z \in {\mathbb {C}} {\backslash } L$,

$$\begin{aligned} \mathfrak {I}\left( S_{\Lambda }(z) \right) = \lim _{c \rightarrow \infty } \sum _{\lambda \in L \cap [-c,c]} \mathfrak {I}\left( \frac{\gamma _{\lambda }}{\lambda -z} \right)&= \lim _{c \rightarrow \infty } \sum _{\lambda \in L \cap [-c,c]} \frac{ - \gamma _{\lambda } \, \mathfrak {I}(\lambda -z)}{ \mathfrak {R}^2(\lambda -z) + \mathfrak {I}^2(\lambda - z) }\\&= \lim _{c \rightarrow \infty } \sum _{\lambda \in L \cap [-c,c]} \frac{ \gamma _{\lambda } \mathfrak {I}(z)}{ \mathfrak {R}^2(\lambda -z) + \mathfrak {I}^2(z) }. \end{aligned}$$

If $z \notin {\mathbb {R}}$, each term of the last sum is nonzero and has the same sign as $\mathfrak {I}(z)$. One deduces that $\mathfrak {I}\left( S_{\Lambda }(z) \right)$ has the same properties, and then $S_{\Lambda }(z) \notin {\mathbb {R}}$.

Now, the interlacing property implies that for any finite interval *I*,

$$\begin{aligned} | {\text {Card}} (L' \cap I) - {\text {Card}} (L \cap I) | \le 2. \end{aligned}$$

If $\Lambda$ satisfies the assumptions of Proposition 9, then for $a, b \in {\mathbb {R}}$ and for *x* going to infinity,

$$\begin{aligned} {\text {Card}} (L' \cap [0,x] ) = {\text {Card}} (L \cap [0,x] ) + O(1) = O(x) + O(1) = O(x) \end{aligned}$$

and

$$\begin{aligned}&{\text {Card}} (L' \cap [0,x+a] ) - {\text {Card}} (L' \cap [-x+b,0] ) = {\text {Card}} (L \cap [0,x+a] ) \\&\quad - {\text {Card}} (L \cap [-x+b,0] ) + O(1) = O(x/\log ^2x). \end{aligned}$$

$$\square $$

Proposition 10 shows that the Stieltjes transform gives a way to construct a discrete subset of ${\mathbb {R}}$ from another, provided that we get a family $(\gamma _j)_{j \in {\mathbb {Z}}}$ of weights and a parameter $h \in {\mathbb {R}}$. In the next section, we use and randomize this procedure in order to define a family of Markov chains satisfying some remarkable properties.

## Stieltjes Markov chains

In order to put some randomness in the construction above, we need to define precisely a measurable space in which the point processes will be contained. The choice considered here is the following:

- We define ${\mathcal {L}}$ as the family of all the discrete subsets *L* of ${\mathbb {R}}$, unbounded from above and from below, and satisfying the assumptions of Proposition 9, i.e. for *x* going to infinity, ${\text {Card}} (L \cap [0,x] ) = O(x)$ and for all $a, b \in {\mathbb {R}}$, ${\text {Card}} (L \cap [0,x+a] ) - {\text {Card}} (L \cap [-x+b,0] ) = O(x/\log ^2 x)$.
- We define, on ${\mathcal {L}}$, the $\sigma$-algebra ${\mathcal {A}}$ generated by the maps $L \mapsto {\text {Card}} (L \cap I)$ for all open, bounded intervals $I \subset {\mathbb {R}}$, which is also the $\sigma$-algebra generated by the maps $L \mapsto {\text {Card}} (L \cap B)$ for all Borel sets $B \subset {\mathbb {R}}$.

A similar choice of measurable space has to be made for the weights $(\gamma _j)_{j \in {\mathbb {Z}}}$:

- We define $\Gamma$ as the family of doubly infinite sequences $(\gamma _j)_{j \in {\mathbb {Z}}}$ satisfying the assumptions of Proposition 9, i.e. for *k* going to infinity,
  $$\begin{aligned} \sum _{j=0}^k \gamma _j = ck + O(k/\log ^2 k) \; \; \mathrm { and } \; \; \sum _{j=0}^k \gamma _{-j} = ck + O(k/\log ^2 k), \end{aligned}$$
  where $c > 0$ is a constant.
- We define, on $\Gamma$, the $\sigma$-algebra ${\mathcal {C}}$ generated by the coordinate maps $\gamma _j$, $j \in {\mathbb {Z}}$.

Let ${\mathcal {D}}$ be the map from ${\mathcal {L}} \times \Gamma \times {\mathbb {R}}$ to ${\mathcal {L}}$, defined by:

$$\begin{aligned} {\mathcal {D}}(L, (\gamma _j)_{j \in {\mathbb {Z}}}, h) = S^{-1}_{ \sum _{j \in {\mathbb {Z}}} \gamma _j \delta _{\lambda _j}} (h), \end{aligned}$$

where $\lambda _j$ is the unique increasing labeling of *L* so that $\lambda _{-1}<0\le \lambda _0$. Proposition 10 shows that this is indeed a map to ${\mathcal {L}}$. It is easy to show that ${\mathcal {D}}$ is measurable. Now for any probability measure $\Pi$ on $\Gamma \times {\mathbb {R}}$, it naturally defines a Markov chain $(X_k)_{k \ge 0}$ on ${\mathcal {L}}$. To get $X_{k+1}$ from $X_k$, just take a fresh sample $G_k$, independent of $X_k$ and its past, distributed according to the measure $\Pi$, and set

$$\begin{aligned} X_{k+1}={\mathcal {D}}(X_k,G_k). \end{aligned}$$

By construction, $(X_k)_{k \ge 0}$ is then a time-homogeneous Markov chain.

Clearly, if the distribution of $X_0$ is invariant under translations of ${\mathbb {R}}$, and the distribution of the $((\gamma _j)_{j \in {\mathbb {Z}}},h)$ in $G_k$ is invariant under translations of the indices *j*, it follows that $X_1$ also has a translation-invariant distribution. Here, the invariance of $((\gamma _j)_{j \in {\mathbb {Z}}},h)$ by translation of the indices *j* is used because translating a set of points in ${\mathbb {R}}$ can shift the labeling of the points ($\lambda _{-1}<0\le \lambda _0$).

There are two important examples of probability measures $\Pi$ for which this construction applies:

- Under $\Pi$, $(\gamma _j)_{j \in {\mathbb {Z}}}$ is a family of i.i.d, square-integrable random variables, and *h* is independent of $(\gamma _j)_{j \in {\mathbb {Z}}}$.
- Under $\Pi$, $(\gamma _j)_{j \in {\mathbb {Z}}}$ is a family of random variables, *n*-periodic for some $n \ge 1$, such that $(\gamma _0, \gamma _1, \dots , \gamma _{n-1}) = (\gamma _1, \dots , \gamma _{n-1}, \gamma _0)$ in law, and *h* is independent of $(\gamma _j)_{j \in {\mathbb {Z}}}$.

The fact that $(\gamma _j)_{j \in {\mathbb {Z}}}$ is almost surely in $\Gamma$ comes from the law of the iterated logarithm in the first example, and directly from the periodicity in the second example.

## Periodic Stieltjes Markov chains

Consider the case when

$$\begin{aligned} \Lambda =\sum _{j\in {\mathbb {Z}}} \gamma _j \delta _{\lambda _j} \end{aligned}$$

is invariant by translation by $2\pi n$, and when there are *n* point masses in every interval of length $2\pi n$ with total weight 2*n*. In this case, $\Lambda$ can be thought as 2*n* times the lifting of the measure

$$\begin{aligned} \sigma =\sum _{j=0}^{n-1} \frac{\gamma _j}{2n} \delta _{e^{i{\lambda _j}/n}} \end{aligned}$$

on the unit circle ${\mathbb {U}}$ under a covering map. Moreover, with $u=e^{iz/n}$ the Stieltjes transform of $\Lambda$ can be expressed in terms of $\sigma$ by

$$\begin{aligned} S_\Lambda (z) = \sum _{j=0}^{n-1} i \frac{\gamma _j}{2n} \frac{e^{i\lambda _j/n} + u}{e^{i\lambda _j/n} - u}. \end{aligned}$$

Indeed, periodicity implies that for $z\notin L$, we have

$$\begin{aligned} S_\Lambda (z)&= \underset{k \rightarrow \infty }{\lim } \sum _{j = - k n}^{ kn-1} \frac{\gamma _j}{\lambda _j - z} = \underset{k \rightarrow \infty }{\lim } \sum _{j=0}^{n-1} \gamma _j \, \left( \sum _{\ell =-k}^{k-1} \frac{1}{2 \pi n\ell + \lambda _j - z} \right) \\&= \sum _{j=0}^{n-1} \gamma _j \left( \underset{k \rightarrow \infty }{\lim } \sum _{\ell =-k}^{k-1} \frac{1}{2 \pi n \ell + \lambda _j - z} \right) = \frac{1}{2n} \, \sum _{j=0}^{n-1} \gamma _j \cot \left( \frac{\lambda _j - z}{2n} \right) . \end{aligned}$$

Therefore, if we set $\rho _j := \gamma _j/2n$ and $u_j = e^{i \lambda _j/n}$, we can check that ${\mathcal {D}} (L, (\gamma _n)_{n \in {\mathbb {Z}}}, h)$ is the set of $z \in {\mathbb {R}}$, such that $e^{iz/n}$ satisfies (2), for $h = i(1+\eta )/(1-\eta )$.

This property shows that the lifting $u \mapsto \{z \in {\mathbb {R}}, e^{iz/n} = u \}$ from ${\mathbb {U}}$ to ${\mathbb {R}}$ defined above transforms the Markov chain defined in Sect. 3 to the Markov chain defined in Sect. 5. In particular, from Propositions 5 and 6, we deduce the following results:

### Theorem 11

Let $\Pi$ be a probability measure on the space $(\Gamma \times {\mathbb {R}}, {\mathcal {C}} \otimes {\mathcal {B}}({\mathbb {R}}))$, under which the following holds, for some integer $n \ge 1$:

- Almost surely under $\Pi$, $(\gamma _n)_{n \in {\mathbb {Z}}}$ is *n*-periodic, and $\sum _{j=0}^{n-1} \gamma _j = 2n$.
- The sequence $(\gamma _j)_{j \in {\mathbb {Z}}}$ is independent of *h*.

Let ${\mathbb {Q}}$ be a probability on $({\mathcal {L}}, {\mathcal {A}})$ under which almost surely, the set *L* is $(2n \pi )$-periodic and contains exactly *n* points in the interval $[0, 2 \pi n)$: in this case, there exists a sequence $( u_1, \dots , u_{n})$ of elements of ${\mathbb {U}}$, with increasing argument in $[0, 2\pi )$, and such that

$$\begin{aligned} L = \{ z \in {\mathbb {R}}, e^{iz/n} \in \{ u_1, \dots , u_{n}\} \}. \end{aligned}$$

Under the probability ${\mathbb {Q}} \otimes \pi$, one can define a random probability measure $\sigma$ on the unit circle by:

$$\begin{aligned} \sigma := \frac{1}{2n} \sum _{j=1}^{n} \gamma _j \delta _{u_j}. \end{aligned}$$

Let us assume that the joint law of the Verblunsky coefficients $(\alpha _0, \dots , \alpha _{n-1})$ of $\sigma$ is invariant by rotation, i.e. for all $u \in {\mathbb {U}}$,

$$\begin{aligned} (\alpha _0 u, \dots \alpha _{n-1} u) = (\alpha _0, \dots , \alpha _{n-1}) \end{aligned}$$

in distribution. Then, the probability measure ${\mathbb {Q}}$ is an invariant measure for the Markov chain associated to $\pi$.

### Theorem 12

Let $\beta > 0$, $n \ge 1$, and let $\Pi _{n,\beta }$ be a probability measure under which the following holds almost surely:

- The sequence $(\gamma _n)_{n \in {\mathbb {Z}}}$ is *n*-periodic.
- The tuple $(\gamma _0/2n, \dots , \gamma _{n-1}/2n)$ follows a Dirichlet distribution with all parameters equal to $\beta /2$.
- The sequence $(\gamma _j)_{j \in {\mathbb {Z}}}$ is independent of *h*.

Let ${\mathbb {Q}}_{n, \beta }$ be the distribution of the set

$$\begin{aligned} \{z \in {\mathbb {R}}, e^{iz/n} \in V\}, \end{aligned}$$

where *V* is a subset of ${\mathbb {U}}$ following ${\mathbb {P}}_{n,\beta }$, i.e. a circular beta ensemble with parameter $\beta$. Then, ${\mathbb {Q}}_{n, \beta }$ is an invariant measure for the Markov chain associated to $\Pi _{n, \beta }$.

In the next section, we will let $n \rightarrow \infty$ and we will obtain a similar result in which the variables $(\gamma _n)_{n \ge 1}$ will be independent and identically distributed.

## An invariant measure for independent gamma random variables

In Theorem 12, we have found an invariant measure on ${\mathcal {L}}$, corresponding to a measure $\Pi _{n,\beta }$ under which the sequence $(\gamma _j)_{j \in {\mathbb {Z}}}$ is periodic, each period forming a renormalized Dirichlet distribution. For $n \ge 1$ and $\beta > 0$ fixed, and under $\Pi _{n,\beta }$, the sequence $(\gamma _j)_{j \in {\mathbb {Z}}}$ can be written in function of a sequence $(g_j)_{j \in {\mathbb {Z}}}$ of i.i.d Gamma variables with parameter $\beta /2$, as follows:

$$\begin{aligned} \gamma _{j} = \frac{2 n g_k}{\sum _{-n/2 < \ell \le n/2} g_{\ell }}, \end{aligned}$$

where $-n/2 < k \le n/2$ and $k \equiv j$ modulo *n*. Here, a Gamma variable with parameter $\theta > 0$ is normalized in such a way that it has density $x \mapsto (\Gamma (\theta ))^{-1} x^{\theta -1 } e^{-x}$ with respect to the Lebesgue measure.

For $\beta$ fixed, if we construct the sequence $(\gamma _j)_{j \in {\mathbb {Z}}}$ for all values of *n*, starting with the same sequence $(g_j)_{j \in {\mathbb {Z}}}$, we obtain, by the law of large numbers, that for all $j \in {\mathbb {Z}}$, $\gamma _j$ tends almost surely to $4 g_j/\beta$ when *n* goes to infinity. Hence, if we want to make $n \rightarrow \infty$ in Theorem 12, we should consider a measure $\Pi _{\beta }$ under which $(\beta \gamma _j/4)_{j \in {\mathbb {Z}}}$ is a sequence of i.i.d. Gamma random variables of parameter $\beta /2$.

On the other hand, for *n* going to infinity, the probability ${\mathbb {Q}}_{n, \beta }$ converges to a limiting measure ${\mathbb {Q}}_{\beta }$, which is the distribution of the so-called $\mathrm {Sine}_{\beta }$ *point process*, constructed in [13, 19].

Therefore, taking the limit $n \rightarrow \infty$ in Theorem 12 suggests the following result, whose proof is given below:

### Theorem 13

Let $\beta > 0$, and let $\Pi _{\beta }$ be a probability measure under which the random variables *h* and $(\gamma _j)_{j \in {\mathbb {Z}}}$ are all independent, $\gamma _j$ being equal to $4/\beta$ times a gamma random variable of parameter $\beta /2$. Then, the law ${\mathbb {Q}}_{\beta }$ of the $\mathrm {Sine}_{\beta }$ point process is carried by the space ${\mathcal {L}}$ and it is an invariant measure for the Markov chain $X_k$ associated to $\Pi _{\beta }$.

Moreover, for the stationary Markov chains $X_{n,k}$ defined of Theorem 12 with same *h*, for every *k*, $(X_{n,0},\ldots , X_{n,k})$ converges in law to $(X_0,\ldots , X_k)$ as $n\rightarrow \infty$, for the topology of locally weak convergence.

### Remark 14

Since the variables $(\gamma _j)_{j \in {\mathbb {Z}}}$ are i.i.d. and square-integrable, we have already checked that the Markov chain associated to $\Pi _{\beta }$ is well-defined, as soon as its initial distribution is fixed and carried by ${\mathcal {L}}$. A consequence of Proposition 16 below is that the probability measure ${\mathbb {Q}}_{\beta }$ is indeed carried by ${\mathcal {L}}$, which means the following: if *L* is the set of points corresponding to a $\mathrm {Sine}_{\beta }$ process, then *L* is unbounded from above and from below, for *x* going to infinity, ${\text {Card}} (L \cap [0,x] ) = O(x)$ and for all $a, b \in {\mathbb {R}}$, ${\text {Card}} (L \cap [0,x+a] ) - {\text {Card}} (L \cap [-x+b,0] ) = O(x/\log ^2 x)$.

In order to show the theorem just above, we will use the following results, proven in [15]:

### Proposition 15

Let *L* be a random set of points in ${\mathbb {R}}$, whose distribution is ${\mathbb {Q}}_{n, \beta }$ or ${\mathbb {Q}}_{\beta }$. Then, there exists $C > 0$, depending on $\beta$ but not on *n*, such that for all $x > 0$,

$$\begin{aligned} {\mathbb {E}} [ \left( {\text {Card}} (L \cap [0,x]) - x/2 \pi \right) ^2] \le C \log (2+x) \end{aligned}$$

and

$$\begin{aligned} {\mathbb {E}} [ \left( {\text {Card}} (L \cap [-x,0]) - x/2 \pi \right) ^2] \le C \log (2+x). \end{aligned}$$

### Proposition 16

For $n \ge 1$ integer, let $L_n$ be a random set of points in ${\mathbb {R}}$, whose distribution is ${\mathbb {Q}}_{n, \beta }$. Let $L_{\infty }$ be a set of points whose distribution is ${\mathbb {Q}}_{\beta }$, and let $\alpha > 1/3$. Then, there exists a tight family $(C_n)_{n \in \{1,2,3, \dots , \infty \}}$ of random variables with values in $(0, \infty )$, such that almost surely, for all $n \in \{1,2,3, \dots , \infty \}$, $x \ge 0$,

$$\begin{aligned} |{\text {Card}} (L_n \cap [0,x]) - x/2 \pi | \le C_n (1+x)^{\alpha }, \end{aligned}$$

and

$$\begin{aligned} |{\text {Card}} (L_n\cap [-x,0]) - x/2 \pi | \le C_n (1+x)^{\alpha }. \end{aligned}$$

### Remark 17

For finite $n \ge 1$, the periodicity of $L_n$ implies that $|{\text {Card}} (L_n \cap [0,x]) - x/2 \pi |$ is almost surely bounded when *x* varies. Hence, the result above becomes trivial for *n* finite if we allow the family $(C_n)_{n \in \{1,2,3, \dots \}}$ not to be tight. Moreover, we expect that it remains true for any $\alpha > 0$, and not only for $\alpha > 1/3$.

### Proof of Theorem 13

Let $\Pi _{\beta }$ be a probability measure which satisfies the assumptions of Theorem 13, and for $n \ge 1$, let $\Pi _{n,\beta }$ be a measure satisfying the assumptions of Theorem 12, for the same value of $\beta$. We also assume that the law of *h* is the same under $\Pi _{n,\beta }$ and under $\Pi _{\beta }$ (note that $\Pi _{n,\beta }$ and $\Pi _{\beta }$ are uniquely determined by this law). By the discussion preceding the statement of Theorem 13, it is possible, by using a unique family $(g_j)_{j \in {\mathbb {Z}}}$ of i.i.d. gamma variables with parameter $\beta /2$, to construct some random sequences $(\gamma _j)_{j \in {\mathbb {Z}}}$ and $(\gamma ^{n}_j)_{j \in {\mathbb {Z}}}$ (for all $n \ge 1$) and an independent real-valued random variable *h*, such that the following holds:

- $((\gamma _j)_{j \in {\mathbb {Z}}}, h)$ follows the law $\Pi _{\beta }$.
- For all $n \ge 1$, $((\gamma ^n_j)_{j \in {\mathbb {Z}}}, h)$ follows the law $\Pi _{n,\beta }$.
- For all $j \in {\mathbb {Z}}$, $\gamma ^n_j$ tends almost surely to $\gamma _j$ when *n* goes to infinity.

Now, for all $n \ge 1$, let $L_n$ be a point process following the distribution ${\mathbb {Q}}_{n, \beta }$, and let *L* be a point process following ${\mathbb {Q}}_{\beta }$. We already know that $L_n \in {\mathcal {L}}$ almost surely. From Proposition 16 under ${\mathbb {Q}}_{\beta }$, we immediately deduce the weaker estimates ${\text {Card}} (L \cap [0,x]) = x/2 \pi + O(x/\log ^2 x)$ and ${\text {Card}} (L \cap [-x,0]) = x/2 \pi + O(x/\log ^2 x)$ for *x* going to infinity, which means that $L \in {\mathcal {L}}$ almost surely: ${\mathbb {Q}}_{\beta }$ is carried by ${\mathcal {L}}$.

Moreover, by [13], the measure ${\mathbb {Q}}_{n,\beta }$ tends to ${\mathbb {Q}}_{\beta }$ when *n* goes to infinity, in the following sense: for all functions *f* from ${\mathbb {R}}$ to ${\mathbb {R}}_+$, $C^{\infty }$ and compactly supported, one has

10 $$\begin{aligned} \sum _{x \in L_n} f(x) \underset{n \rightarrow \infty }{\longrightarrow } \sum _{x \in L} f(x) \end{aligned}$$

in distribution. By the Skorokhod representation theorem (see [4, Theorem 6.7]) one can assume that the convergence (10) holds almost surely, and one can also suppose that $(L_n)_{n \ge 1}$ and *L* are independent of $(\gamma ^n_j)_{n \ge 1, j \in {\mathbb {Z}}}$, $(\gamma _j)_{j \in {\mathbb {Z}}}$ and *h*.

For $n \ge 1$, let $(\lambda ^n_j)_{j \in {\mathbb {Z}}}$ be the strictly increasing sequence containing each point of $L_n$, $\lambda ^n_0$ being the smallest nonnegative point, and let $(\lambda _j)_{j \in {\mathbb {Z}}}$ be the similar sequence associated to *L*. One can check that the convergence (10) and the fact that ${\mathbb {P}} [0 \in L] = 0$ imply that for all $j \in {\mathbb {Z}}$, $\lambda ^n_j$ converges almost surely to $\lambda _j$ when *n* goes to infinity. Indeed, for $j \ge 0$ and $\epsilon \in (0, \lambda _j/10)$, let us consider a test function *f* taking values in [0, 1], equal to 1 on $[\epsilon , \lambda _j]$ and to 0 on ${\mathbb {R}} {\backslash }[0, \lambda _j + \epsilon ]$. For $\epsilon$ small enough, *L* has no point in $[0, \epsilon ]$ and $j+1$ points in $[0,\lambda _j]$, which implies that the sum of *f* at the points of *L* is at least $j+1$. Hence, the sum of *f* at the points of $L_n$ is at least $j+(1/2)$ for *n* large enough, which implies that $L_n$ has at least $j + (1/2)$ points, and then at least $j+1$ points, in the interval $[0, \lambda _j + \epsilon ]$. This implies $\lambda ^n_j \le \lambda _j + \epsilon$. Similarly, if we take *f* in [0, 1], equal to 1 on $[0, \lambda _j - 2 \epsilon ]$ and to 0 on ${\mathbb {R}} {\backslash }[-\epsilon , \lambda _j - \epsilon ]$, the sum of *f* at points on *L* is at most *j* for $\epsilon$ small enough (because *L* has no point in $[-\epsilon ,0]$), which implies that the sum of *f* at points on $L_n$ is at most $j+ (1/2)$ for *n* large enough, and then $L_n$ has at most *j* points in $[0, \lambda _j - 2 \epsilon ]$, i.e. $\lambda ^n_j \ge \lambda _j - 2 \epsilon$. The case $j < 0$ can be treated similarly.

Now, for all $c > 0$, $z \in {\mathbb {C}} {\backslash }\left( L \cup \left( \bigcup _{n \ge 1} L_n \right) \right)$, let us take the following notation:

$$\begin{aligned} S_{n,c}(z):= & {} \sum _{j \in {\mathbb {Z}}} \, \frac{\gamma ^n_j}{\lambda ^n_j - z} \, \mathbf {1}_{|\lambda ^n_j| \le c}, \; \; S_{c}(z) := \sum _{j \in {\mathbb {Z}}} \, \frac{\gamma _j}{\lambda _j - z} \, \mathbf {1}_{|\lambda _j| \le c}, \\ S_N(z):= & {} \lim _{c \rightarrow \infty } S_{N,c} (z), \; \; S(z) := \lim _{c \rightarrow \infty } S_c (z). \end{aligned}$$

Almost surely, all the points of *L* and $L_n$ ($n \ge 1$) are irrational. If this event occurs, then for all $c \in {\mathbb {Q}}^*_+$, there exists almost surely a random finite interval (possibly empty) $I_c$ such that $|\lambda _j| \le c$ if and only if $j \in I_c$, and for all $n \ge 1$ large enough, $|\lambda ^n_j| \le c$ if and only if $j \in I_c$. Hence, for all $c \in {\mathbb {Q}}^*_+$, $z \in {\mathbb {Q}}$, one has almost surely

$$\begin{aligned} S_{n,c}(z) = \sum _{j \in I_c} \frac{\gamma ^n_j}{\lambda ^n_j - z}, \; \; S_{c}(z) := \sum _{j \in I_c} \, \frac{\gamma _j}{\lambda _j - z}, \end{aligned}$$

if *n* is large enough. Since $I_c$ is finite, $\gamma ^n_j$ tends a.s. to $\gamma _j$, and $\lambda ^n_j$ tends a.s. to $\lambda _j$ when *n* goes to infinity, one deduces that almost surely, for all $c \in {\mathbb {Q}}^*_+$, $z \in {\mathbb {Q}}$,

11 $$\begin{aligned} S_{n,c}(z) \underset{n \rightarrow \infty }{\longrightarrow } S_c(z). \end{aligned}$$

On the other hand, by (8), and by the fact that *c* and $-c$ are a.s. not in *L* or in $L_n$, one deduces that almost surely, for all $c \in {\mathbb {Q}}^*_+$, $z \in {\mathbb {Q}}$ such that $c > 2|z| \vee 1$, and for all $n \ge 1$,

$$\begin{aligned}&S_n (z) - S_{n,c} (z) = \int _{c}^{\infty } \frac{ \Lambda _n([c, \mu ]) - \Lambda _n([-\mu , - c])}{\mu ^2} \, d \mu \\&\quad + \int _{c}^{\infty } \left( \frac{ (2 z \mu - z^2) (\Lambda _n([c, \mu ]) )}{\mu ^2 (\mu -z)^2} + \frac{(2 z \mu + z^2) (\Lambda _n([-\mu , -c]) )}{\mu ^2 (\mu +z)^2} \right) \, d\mu \end{aligned}$$

and

$$\begin{aligned}&S (z) - S_{c} (z) = \int _{c}^{\infty } \frac{ \Lambda ([c, \mu ]) - \Lambda ([-\mu , - c])}{\mu ^2} \, d \mu \\&\quad +\, \int _{c}^{\infty } \left( \frac{ (2 z \mu - z^2) (\Lambda ([c, \mu ]) )}{\mu ^2 (\mu -z)^2} + \frac{(2 z \mu + z^2) (\Lambda ([-\mu , -c]) )}{\mu ^2 (\mu +z)^2} \right) \, d\mu , \end{aligned}$$

where $\Lambda _n:= \sum _{j \in {\mathbb {Z}}} \gamma ^n_j \delta _{\lambda ^n_j}$ and $\Lambda := \sum _{j \in {\mathbb {Z}}} \gamma _j \delta _{\lambda _j}$. If for any bounded interval *I*, one defines $\Lambda ^{(0)}_n (I) := \Lambda _n(I) - {\mathbb {E}} [ \Lambda _n(I)]$ and $\Lambda ^{(0)} (I) := \Lambda (I) - {\mathbb {E}} [ \Lambda (I)]$, one has by (7), the triangle inequality, and the fact that ${\mathbb {E}} [ \Lambda _n(I)]$ is proportional to the Lebesgue measure on *I*:

$$\begin{aligned} |S_n(z) - S_{n,c}(z)|&\le \int _c^{\infty } \frac{ |\Lambda ^{(0)}_n ([c, \mu ])| + |\Lambda ^{(0)}_n ([-\mu , -c])|}{\mu ^2} \, d \mu \\&\quad + \int _c^{\infty } \frac{20|z| \, d \mu }{\mu ^3} \, \left[ C_1 \mu + |\Lambda ^{(0)}_n ([c, \mu ])| + |\Lambda ^{(0)}_n ([-\mu , -c])|\right] \\&\le C_2 (1+|z|) \left( \frac{1}{c} + \int _c^{\infty } \frac{ |\Lambda ^{(0)}_n ([c, \mu ])| {+} |\Lambda ^{(0)}_n ([-\mu , -c])|}{\mu ^2} \, d \mu \right) , \end{aligned}$$

where $C_1, C_2 > 0$ are universal constants. Since the distribution of $L_n$ is invariant by translation (recall that its points are the rescaled arguments of the circular beta ensemble on the unit circle), one has

$$\begin{aligned} {\mathbb {E}}[ |\Lambda ^{(0)}_n ([c, \mu ])|] = {\mathbb {E}}[ |\Lambda ^{(0)}_n ([-\mu , -c])|]= {\mathbb {E}}[ |\Lambda ^{(0)}_n ([0, \mu -c])|] \end{aligned}$$

and

$$\begin{aligned} {\mathbb {E}}[|S_n(z) - S_{n,c}(z)|] \le C_3 (1+|z|) \left( \frac{1}{c} + \int _0^{\infty } \frac{ {\mathbb {E}}[ |\Lambda ^{(0)}_n ([0, \nu ])| ]}{(\nu + c)^2} \, d \nu \right) , \end{aligned}$$

where $C_3 > 0$ is a universal constant. Similarly,

$$\begin{aligned} {\mathbb {E}}[|S(z) - S_{c}(z)|] \le C_3 (1+|z|) \left( \frac{1}{c} + \int _0^{\infty } \frac{ {\mathbb {E}}[ |\Lambda ^{(0)} ([0, \nu ])| ]}{(\nu + c)^2} \, d \nu \right) . \end{aligned}$$

Now, from Proposition 15 under ${\mathbb {Q}}_{n, \beta }$ and ${\mathbb {Q}}_{\beta }$, one immediately deduces that

12 $$\begin{aligned} \int _0^{\infty } \frac{ {\mathbb {E}}[ |\Lambda ^{(0)} ([0, \nu ])| ]+ \sup _{n \ge 1} {\mathbb {E}}[ |\Lambda _n^{(0)} ([0, \nu ])| ]}{(1+\nu )^2} \, d\nu < \infty . \end{aligned}$$

Hence, by dominated convergence, there exists a function $\phi$ from $[1, \infty )$ to ${\mathbb {R}}^*_+$, tending to zero at infinity, such that

$$\begin{aligned} {\mathbb {E}}[|S_n(z) - S_{n,c}(z)|] \le (1+|z|) \, \phi (c) \end{aligned}$$

and

$$\begin{aligned} {\mathbb {E}}[|S(z) - S_{c}(z)|] \le (1+|z|) \, \phi (c). \end{aligned}$$

We deduce that for all $c \in {\mathbb {Q}}^*_+$, $z \in {\mathbb {Q}}$ such that $c > 2|z| \vee 1$, $n \ge 1$ and $\epsilon > 0$,

$$\begin{aligned} {\mathbb {P}} [ |S(z) - S_{n} (z)| \ge \epsilon ]&\le {\mathbb {P}} [ |S_c(z) - S_{n,c}(z) | \ge \epsilon /3 ] + {\mathbb {P}} [ |S(z) - S_{c}(z) | \ge \epsilon /3 ] \\&\quad + \,{\mathbb {P}} [ |S_n(z) - S_{n,c}(z)| \ge \epsilon /3 ] \\&\le {\mathbb {P}} [ |S_c(z) - S_{n,c}(z) | \ge \epsilon /3 ] + \frac{6}{\epsilon } \, (1+|z|) \, \phi (c). \end{aligned}$$

By the almost sure convergence (11), which implies the corresponding convergence in probability, one deduces

$$\begin{aligned} \underset{n \rightarrow \infty }{\lim \sup } \; {\mathbb {P}} [ |S(z) - S_{n} (z)| \ge \epsilon ] \le \frac{6}{\epsilon } \, (1+|z|) \, \phi (c). \end{aligned}$$

Now, by taking $z \in {\mathbb {Q}}$ fixed, $c \in {\mathbb {Q}}$ going to infinity and then $\epsilon \rightarrow 0$, one deduces that for all $z \in {\mathbb {Q}}$,

$$\begin{aligned} S_n(z) \underset{n \rightarrow \infty }{\longrightarrow } S(z) \end{aligned}$$

in probability. By considering diagonal extraction of subsequences, one deduces that there exists a strictly increasing sequence $(n_k)_{k \ge 1}$ of integers, such that almost surely,

13 $$\begin{aligned} S_{n_k} (z) \underset{k \rightarrow \infty }{\longrightarrow } S(z) \end{aligned}$$

for all $$z \in {\mathbb {Q}}$$.

Now, for all $j \in {\mathbb {Z}}$, $n \ge 1$, let $\mu ^n_j$ (resp. $\mu _j$) be the unique point of ${\mathcal {D}} (L_n, (\gamma ^n_j)_{j \in {\mathbb {Z}}}, h)$ (resp. ${\mathcal {D}} (L, (\gamma _j)_{j \in {\mathbb {Z}}}, h)$) which lies in the interval $(\lambda ^n_j, \lambda ^n_{j+1})$ (resp. $(\lambda _j, \lambda _{j+1})$). Let us fix $j \in {\mathbb {Z}}$, $\epsilon > 0$, and let us consider two random rational numbers $q_1$ and $q_2$ such that almost surely,

$$\begin{aligned} (\mu _j - \epsilon ) \vee \lambda _j< q_1< \mu _j< q_2 < (\mu _j + \epsilon ) \wedge \lambda _{j+1}, \end{aligned}$$

which implies that

$$\begin{aligned} S(q_1)< h < S(q_2). \end{aligned}$$

By (13), one deduces that almost surely, for *k* large enough,

$$\begin{aligned} S_{n_k}(q_1)< h < S_{n_k}(q_2), \end{aligned}$$

which implies that ${\mathcal {D}} (L_{n_k}, (\gamma ^{n_k}_j)_{j \in {\mathbb {Z}}}, h)$ has at least one point in the interval $(q_1,q_2)$. On the other hand, since $\lambda ^n_j$ (resp. $\lambda ^n_{j+1}$) tends a.s. to $\lambda _j$ (resp. $\lambda _{j+1}$) when *n* goes to infinity, one has almost surely, for *k* large enough,

$$\begin{aligned} \lambda ^{n_k}_j< q_1< q_2< \lambda ^{n_k}_{j+1}. \end{aligned}$$

Hence, ${\mathcal {D}} (L_{n_k}, (\gamma ^{n_k}_j)_{j \in {\mathbb {Z}}}, h)$ has exactly one point in $(q_1, q_2)$, and this point is necessarily $\mu ^{n_k}_j$. One deduces that almost surely, $|\mu ^{n_k}_j - \mu _j| \le \epsilon$ for *k* large enough, which implies, by taking $\epsilon \rightarrow 0$, that $\mu ^{n_k}_j$ converges almost surely to $\mu _j$ when *k* goes to infinity.

Now, let *f* be a function from ${\mathbb {R}}$ to ${\mathbb {R}}_+$, $C^{\infty }$ and compactly supported. Since *L* is locally finite, there exists a.s. an integer $j_0 \ge 1$ such that the support of *f* is included in $(\lambda _{-j_0}, \lambda _{j_0})$, and then in $(\lambda ^{n_k}_{-j_0}, \lambda ^{n_k}_{j_0})$ for *k* large enough, which implies that $f(\mu ^{n_k}_j) = f(\mu _j) = 0$ for $|j| > j_0$. Hence, a.s., there exists $j_0, k_0 \ge 1$, such that for $k \ge k_0$,

$$\begin{aligned} \sum _{j \in {\mathbb {Z}}} f(\mu ^{n_k}_j) = \sum _{|j| \le j_0} f(\mu ^{n_k}_j) \end{aligned}$$

and

$$\begin{aligned} \sum _{j \in {\mathbb {Z}}} f(\mu _j) = \sum _{|j| \le j_0} f(\mu _j), \end{aligned}$$

which implies that

14 $$\begin{aligned} \sum _{j \in {\mathbb {Z}}} f(\mu ^{n_k}_j) \underset{k \rightarrow \infty }{\longrightarrow } \sum _{j \in {\mathbb {Z}}} f(\mu _j), \end{aligned}$$

since $f(\mu _j^{n_k})$ tends to $f(\mu _j)$ for each $j \in \{-j_0, -j_0+1, \dots , j_0\}$.

The almost sure convergence (14) holds a fortiori in distribution, which implies that the law of ${\mathcal {D}} (L_{n_k}, (\gamma ^{n_k}_j)_{j \in {\mathbb {Z}}}, h)$ tends to the law of ${\mathcal {D}} (L, (\gamma _j)_{j \in {\mathbb {Z}}}, h)$. On the other hand, by Theorem 12, ${\mathcal {D}} (L_{n_k}, (\gamma ^{n_k}_j)_{j \in {\mathbb {Z}}}, h)$ has distribution ${\mathbb {Q}}_{n_k, \beta }$, and then ${\mathcal {D}} (L, (\gamma _j)_{j \in {\mathbb {Z}}}, h)$ follows the limit of the distribution ${\mathbb {Q}}_{n_k, \beta }$ for *k* tending to infinity, i.e. ${\mathbb {Q}}_{\beta }$. This shows the first part of Theorem 13, and the second part for $k=2$; iterating this argument shows the general *k* case. $\square$

## Properties of continuity for the Stieltjes Markov chain

In the previous section, we have deduced the convergence of the Markov mechanism associated to ${\mathbb {Q}}_{n_k, \beta }$ towards the one corresponding to ${\mathbb {Q}}_{\beta }$ from the convergence of ${\mathbb {Q}}_{n_k, \beta }$ to ${\mathbb {Q}}_{\beta }$ itself, and the convergence of the associated weights. Later in the paper, we will prove similar results related to the Gaussian ensembles, for which the situation is more difficult to handle, in particular because of the lack of symmetry of the G$\beta$E at the macroscopic scale, when we rescale around a non-zero point of the bulk. Moreover, we will have to consider several steps of the Markov mechanism at the same time. That is why we will need a more general result, giving a property of continuity of the Markov mechanism described above, with respect to its initial data.

The main results of the present paper concern convergence in distribution of point processes. In this section, we will assume properties of strong convergence, which can be done with the help of Skorokhod’s representation theorem.

The notion of convergence of holomorphic functions usually considered is the uniform convergence on compact sets. This notion cannot be directly applied to the meromorphic functions involved here, because of the poles on the real line. That is why we will need an appropriate notion of uniform convergence of meromorphic functions.

More precisely, we say that a sequence $(f_n)_{n \ge 1}$ of meromorphic functions on an open set $U \subset {\mathbb {C}}$ converges uniformly to a function *f* from *U* to the Riemann sphere ${\mathbb {C}} \cup \{\infty \}$ if and only if this convergence holds for the distance *d* on ${\mathbb {C}} \cup \{\infty \}$, given by

$$\begin{aligned} d(z_1, z_2) = \frac{|z_2 - z_1|}{ \sqrt{(1+ |z_1|^2)(1+|z_2|^2)}} \end{aligned}$$

for $z_1, z_2 \ne \infty$, and extended by continuity at $\infty$ (*d* corresponds to the distance of the points on the Euclidean sphere, obtained via the inverse stereographic projection). It is a classical result that the limiting function *f* should be meromorphic on *U*. One deduces the following: if a sequence $(f_n)_{n \ge 1}$ of meromorphic functions on ${\mathbb {C}}$ converges to a function *f* from ${\mathbb {C}}$ to ${\mathbb {C}} \cup \{\infty \}$, uniformly on all bounded subsets of ${\mathbb {C}}$, then *f* is meromorphic on ${\mathbb {C}}$. Moreover, the following lemma will be useful:

### Lemma 18

Let $(f_n)_{n \ge 1}$ (resp. $(g_n)_{n \ge 1})$ be a sequence of meromorphic functions on an open set *U*, uniformly convergent (for the distance *d*) to a function *f* (resp. *g*), necessarily meromorphic. We assume that *f* and *g* have no common pole. Then the sequence $(f_n + g_n)_{n \ge 1}$ of meromorphic functions tends uniformly to $f+g$ on all the compact sets of *U*.

### Remark 19

The fact that *f* and *g* have no common pole is needed in general. Indeed, if *U* is a neighborhood of 0, $f_n (z) = f(z) = - z^{-1}$, $g_n(z)= (z+n^{-1})^{-1}$, $g(z) = z^{-1}$, we check that $f_n$ and $g_n$ respectively tend to *f* and *g*, uniformly on compact sets of *U*, for the distance *d*, but $f_n + g_n$ does not uniformly converge to $f+g = 0$ in any neighborhood of 0.

### Proof

Let *K* be a compact subset of *U*, let $z_1, z_2, \dots , z_p$ be the poles of *f* in *K*, and $z'_1, z'_2, \dots , z'_q$ the poles of *g* in *K*. There exists a neighborhood *V* of $\{z_1,z_2, \dots , z_p\}$ containing no pole of *g*, and a neighborhood *W* of $\{z'_1,z'_2, \dots , z'_p\}$ containing no pole of *f*. If $A > 0$ is fixed, one can assume the following (by restricting *V* and *W* if it is needed):

- The infimum of |*f*| on *V* is larger than $2A+1$ and also larger than the supremum of $2|g|+1$ on *V*.
- The infimum of |*g*| on *W* is larger than $2A+1$ and also larger than the supremum of $2|f|+1$ on *W*.

By the assumption of uniform convergence, we deduce, for *n* large enough:

- The infimum of $|f_n|$ on *V* is larger than 2*A* and also larger than the supremum of $2|g_n|$ on *V*.
- The infimum of $|g_n|$ on *W* is larger than 2*A* and also larger than the supremum of $2|f_n|$ on *W*.

Now, for all $z \in V$ and *n* large enough, one has

$$\begin{aligned} |f_n(z) + g_n(z)| \ge |f_n(z)| - |g_n(z)| \ge |f_n(z)| - \frac{ |f_n(z)|}{2} = \frac{ |f_n(z)|}{2} \ge A. \end{aligned}$$

and also

$$\begin{aligned} |f(z) + g(z)| \ge A, \end{aligned}$$

which implies

$$\begin{aligned} d(f_n(z) + g_n(z), f(z) + g(z)) \le 2/A. \end{aligned}$$

Similarly, this inequality is true for $z \in W$. Moreover, there exists a compact set $L \subset K$, containing no pole of *f* or *g*, and such that *K* is included in $L \cup V \cup W$. Since the meromorphic functions *f* and *g* have no pole on the compact set *L*, they are bounded on this set. Since $(f_n)_{n \ge 1}$ (resp. $(g_n)_{n \ge 1}$) converges to *f* (resp. *g*) on *L*, uniformly for the distance *d*, and $(f_n)_{n \ge 1}$ (resp. $(g_n)_{n \ge 1}$) is uniformly bounded, the uniform convergence holds in fact for the usual distance. Hence, $(f_n + g_n)_{n \ge 1}$ tends uniformly to $f+g$ on *L* for the usual distance, and a fortiori for *d*: by using the previous bounded obtained in *V* and *W*, one deduces, since *L*, *V* and *W* cover *K*:

$$\begin{aligned} \underset{n \rightarrow \infty }{\lim \sup } \, \underset{z \in K}{\sup } \, d(f_n(z) + g_n(z), f(z) + g(z)) \le 2/A. \end{aligned}$$

Since we can choose $A > 0$ arbitrarily, we are done. $\square$

From this lemma, we deduce the following statement

### Lemma 20

Let $p \ge 1$, and let $(\lambda _k)_{1 \le k \le p}$, $(\lambda _{n,k})_{n \ge 1, 1 \le k \le p}$, $(\gamma _k)_{1 \le k \le p}$, $(\gamma _{n,k})_{n \ge 1, 1 \le k \le p}$ be some complex numbers such that all the $\lambda _k$’s are distincts, all the $\gamma _k$’s are nonzero, and for all $k \in \{1, \dots , p\}$,

$$\begin{aligned} \lambda _{n,k} \underset{n \rightarrow \infty }{\longrightarrow } \lambda _k \end{aligned}$$

and

$$\begin{aligned} \gamma _{n,k} \underset{n \rightarrow \infty }{\longrightarrow } \gamma _k. \end{aligned}$$

Then, one has, for *n* going to infinity, the convergence of the rational function

$$\begin{aligned} z \mapsto \sum _{k=1}^p \frac{\gamma _{n,k}}{\lambda _{n,k} - z} \end{aligned}$$

towards the function

$$\begin{aligned} z \mapsto \sum _{k=1}^p \frac{\gamma _{k}}{\lambda _{k} - z}, \end{aligned}$$

uniformly on all the compact sets, for the distance *d*.

### Proof

Let us first prove the result for $p=1$, which is implied by the following convergence

$$\begin{aligned} \frac{\gamma _{n,1}}{\lambda _{n,1} - z } \underset{n \rightarrow \infty }{\longrightarrow } \frac{\gamma _{1}}{\lambda _{1} - z }, \end{aligned}$$

uniformly on ${\mathbb {C}}$ for the distance *d*. Let us fix $\epsilon > 0$. For *n* large enough, we have $|\lambda _{n,1} - \lambda _{1}| \le \epsilon$ and $|\gamma _{n,1} - \gamma _{1}| \le |\gamma _1|/2$. If these conditions are satisfied and if $|\lambda _{1} - z| \le 2 \epsilon$, then

$$\begin{aligned} \left| \frac{\gamma _{1}}{\lambda _{1} - z } \right| \ge \frac{|\gamma _1|}{2 \epsilon } \end{aligned}$$

and

$$\begin{aligned} \left| \frac{\gamma _{n,1}}{\lambda _{n,1} - z } \right| \ge \frac{|\gamma _1|}{6 \epsilon }, \end{aligned}$$

since $$|\gamma _{n,1}| \ge |\gamma _1|/2$$ and

$$\begin{aligned} |\lambda _{n,1} - z| \le |\lambda _{1} - z| + |\lambda _{n,1} - \lambda _{1}|\le 3 \epsilon . \end{aligned}$$

Hence, there exists $n_0 \ge 1$, independent of *z* satisfying $|\lambda _{1} - z| \le 2 \epsilon$, such that for $n \ge n_0$,

$$\begin{aligned} d\left( \frac{\gamma _{1}}{\lambda _{1} - z }, \frac{\gamma _{n,1}}{\lambda _{n,1} - z } \right) \le d\left( \frac{\gamma _{1}}{\lambda _{1} - z }, \infty \right) + d\left( \infty , \frac{\gamma _{n,1}}{\lambda _{n,1} - z } \right) \le \frac{2 \epsilon }{|\gamma _1|} + \frac{6 \epsilon }{|\gamma _1|} = \frac{8 \epsilon }{|\gamma _1|}. \end{aligned}$$

Similarly, there exists $n_1 \ge 1$ such that for all $n \ge n_1$ and for all *z* satisfying $|\lambda _{1} - z| \ge 2 \epsilon$, one has:

$$\begin{aligned} |\lambda _{n,1} - z| \ge |\lambda _{1} - z| - |\lambda _{n,1} - \lambda _{1}| \ge \epsilon . \end{aligned}$$

This implies:

$$\begin{aligned} \left| \frac{\gamma _{1}}{\lambda _{1} - z } - \frac{\gamma _{n,1}}{\lambda _{n,1} - z } \right|&\le \left| \frac{\gamma _{1} - \gamma _{n,1} }{\lambda _{n,1} - z } \right| + |\gamma _1| \, \left| \frac{1}{\lambda _{1} - z} - \frac{1}{\lambda _{n,1} - z} \right| \\&\le \frac{|\gamma _{1} - \gamma _{n,1} |}{\epsilon } + |\gamma _1| \, \frac{|\lambda _{1} - \lambda _{n,1}|}{(2\epsilon )(\epsilon )}. \end{aligned}$$

Since this quantity does not depend on *z* and tends to zero at infinity, we deduce

$$\begin{aligned} \sup _{z \in {\mathbb {C}}, |\lambda _{1} - z| \ge 2 \epsilon } d\left( \frac{\gamma _{1}}{\lambda _{1} - z }, \frac{\gamma _{n,1}}{\lambda _{n,1} - z } \right) \underset{n \rightarrow \infty }{\longrightarrow } 0. \end{aligned}$$

Since we know that

$$\begin{aligned} \underset{n \rightarrow \infty }{\lim \, \sup } \sup _{z \in {\mathbb {C}}, |\lambda _{1} - z| \le 2 \epsilon } d\left( \frac{\gamma _{1}}{\lambda _{1} - z }, \frac{\gamma _{n,1}}{\lambda _{n,1} - z } \right) \le \frac{8 \epsilon }{|\gamma _1|}, \end{aligned}$$

we get

$$\begin{aligned} \underset{n \rightarrow \infty }{\lim \, \sup } \, \sup _{z \in {\mathbb {C}}} \, d\left( \frac{\gamma _{1}}{\lambda _{1} - z }, \frac{\gamma _{n,1}}{\lambda _{n,1} - z } \right) \le \frac{8 \epsilon }{|\gamma _1|}. \end{aligned}$$

Now, $\epsilon > 0$ can be arbitrarily chosen, and then the lemma is proven for $p=1$. For $p \ge 2$, let us deduce the result of the lemma, assuming that it is satisfied when *p* is replaced by $p-1$. We define the meromorphic functions $(f_n)_{n \ge 1}$, *f*, $(g_n)_{n \ge 1}$, *g* by the formulas:

$$\begin{aligned} f_n(z)= & {} \sum _{k=1}^{p-1} \frac{\gamma _{n,k}}{\lambda _{n,k} - z}, \\ f(z)= & {} \sum _{k=1}^{p-1} \frac{\gamma _{k}}{\lambda _{k} - z}, \\ g_n(z)= & {} \frac{\gamma _{n,p}}{\lambda _{n,p} - z}, \\ g(z)= & {} \frac{\gamma _{p}}{\lambda _{p} - z}. \end{aligned}$$

Let $A > 0$. By the induction hypothesis, we know that $f_n$ converges to *f* when *n* goes to infinity, uniformly on the set $\{z \in {\mathbb {C}}, |z| < 2A\}$ and for the distance *d*. Similarly, by the case $p=1$ proven above, $g_n$ converges to *g*, uniformly on the same set (in fact, uniformly on ${\mathbb {C}}$) and for the same distance. Moreover, the functions *f* and *g* have no common pole, since the numbers $(\lambda _k)_{1 \le k \le p}$ are all distinct. We can then apply Lemma 18 and deduce that $f_n+ g_n$ converges to $f + g$, uniformly on any compact set of $\{z \in {\mathbb {C}}, |z| < 2A\}$, for example $\{z \in {\mathbb {C}}, |z| \le A\}$, and for the distance *d*. Since $A > 0$ can be arbitrarily chosen, we are done. $\square$

We have now the ingredients needed to state the main result of this section. In this theorem, we deal with finite and infinite sequences together. So we will think of $k\mapsto \lambda _k$ as a function from ${\mathbb {Z}}\rightarrow {\mathbb {R}}\cup \{\emptyset \}$, with the convention that summation and other operations are only considered over the values that are different from $\emptyset$. We will also assume that the value $\emptyset$ is taken exactly on the complement of an interval of ${\mathbb {Z}}$.

The statement of the following result is long and technical, but as we will see in the next section, it will be adapted to the problem we are interested in.

### Theorem 21

Let $(\Xi _n)_{n \ge 1}$ be a sequence of discrete simple point measures on ${\mathbb {R}}$ (i.e. sums of Dirac masses at a locally finite set of points), converging to a simple point measure $\Xi$, locally weakly:

15 $$\begin{aligned} \Xi _n \longrightarrow \Xi . \end{aligned}$$

Let $L_n$ denote the support of $\Xi _n$, and *L* the support of $\Xi$. We suppose that there exists $\alpha \in (0,1)$, a family $(\tau _\ell )_{\ell \ge 0}$ of elements of ${\mathbb {R}}_+^*$, with $\tau _\ell \rightarrow 0$ as $\ell \rightarrow \infty$, such that for all $n \ge 1$, $\ell \ge 1$, we have

16 $$\begin{aligned} \int _{{\mathbb {R}}}\frac{{\mathbf {1}}(|\lambda |>\ell )}{|\lambda |^{1+\alpha }} \,d\Xi _n(\lambda ) \le \tau _\ell \end{aligned}$$

Moreover, assume that the limits

17 $$\begin{aligned} h_{n,\ell } = \lim _{\ell '\rightarrow \infty } \int _{{\mathbb {R}}}\frac{{\mathbf {1}}(\ell<|\lambda |<\ell ')}{\lambda }\,d\Xi _n(\lambda ) \end{aligned}$$

exist, and so does the similar limit $h_\ell$ defined in terms of $\Xi$. Assume further that for some $h\in {\mathbb {R}}$, the following equalities are well-defined and satisfied:

18 $$\begin{aligned} \lim _{\ell \rightarrow \infty }\lim _{n\rightarrow \infty } h_{n,\ell } =h, \qquad \lim _{\ell \rightarrow \infty }h_{\ell } =0, \end{aligned}$$

when the limits are restricted to the condition: $\ell \notin L$ and $-\ell \notin L$.

Further, let $(\gamma _{n,k})_{k\in {\mathbb {Z}}}$ be a strictly positive sequence. Suppose it satisfies

19 $$\begin{aligned} \gamma _{n,k} \rightarrow \gamma _k > 0 \end{aligned}$$

for each *k*, as $n\rightarrow \infty$. Also for some ${{\bar{\gamma }}},c>0$ and all $n,m \ge 1$, we assume

20 $$\begin{aligned} \left| \sum _{k=0}^{m-1} \gamma _{n,k} - {{\bar{\gamma }}}m\right|\le & {} c m^{\alpha '}\nonumber \\ \left| \sum _{k=-m}^{-1} \gamma _{n,k} - {{\bar{\gamma }}}m\right|\le & {} c m^{\alpha '} \end{aligned}$$

with $0<(1+\alpha )\alpha '<1$. Let $\lambda ^*$ be a point outside *L*, and consider the weighted version $\Lambda$ of $\Xi$ where the *k*th point after $\lambda ^*$ (for $k \le 0$, the $(1-k)$th point before $\lambda ^*)$ has weight $\gamma _k$. For *n* large enough, one has also $\lambda ^* \notin L_n$: define $\Lambda _n$ similarly. Then the limit

$$\begin{aligned} S_n(z)=\lim _{\ell \rightarrow \infty } \int _{[-\ell ,\ell ]} \frac{1}{\lambda -z}\,d\Lambda _n(\lambda ) \end{aligned}$$

exists for all $z\notin L_n$, is meromorphic with simple poles at $L_n$, and converges, uniformly on compacts with respect to the distance *d* on the Riemann sphere ${\mathbb {C}} \cup \{\infty \}$, to $S(z)+{\bar{\gamma }}h$, where *S* is a meromorphic function with simple poles at *L*, such that for all $z \notin L$,

$$\begin{aligned} S(z)=\lim _{\ell \rightarrow \infty } \int _{[-\ell ,\ell ]} \frac{1}{\lambda -z}\,d\Lambda (\lambda ). \end{aligned}$$

Moreover, for every $h'\in {\mathbb {R}}$, the sum of delta masses $\Xi '_n$ at $S_n^{-1}(h'+{\bar{\gamma }}h)$ converges locally weakly to the sum of delta masses $\Xi '$ at $S^{-1}(h')$, and $(\Xi '_{n})_{n \ge 1}$, and $\Xi '_n$, $\Xi '$ satisfy assumptions equivalent to (15)–(18), i.e.

21 $$\begin{aligned}&\Xi '_n \longrightarrow \Xi ', \end{aligned}$$

22 $$\begin{aligned}&\int _{{\mathbb {R}}}\frac{{\mathbf {1}}(|\lambda |>\ell )}{|\lambda |^{1+\alpha }}\, d\Xi '_n(\lambda ) \le \tau '_\ell \end{aligned}$$

for a family $(\tau '_\ell )_{\ell \ge 0}$ of elements of ${\mathbb {R}}_+^*$, with $\tau '_\ell \rightarrow 0$ as $\ell \rightarrow \infty$,

23 $$\begin{aligned} \lim _{\ell \rightarrow \infty }\lim _{n\rightarrow \infty } h'_{n,\ell } =h, \qquad \lim _{\ell \rightarrow \infty }h'_{\ell } =0, \end{aligned}$$

where

24 $$\begin{aligned} h'_{n,\ell } = \lim _{\ell '\rightarrow \infty } \int _{{\mathbb {R}}}\frac{{\mathbf {1}}(\ell<|\lambda |<\ell ')}{\lambda }\,d\Xi '_n(\lambda ), \quad h'_{\ell } = \lim _{\ell '\rightarrow \infty } \int _{{\mathbb {R}}}\frac{{\mathbf {1}}(\ell<|\lambda |<\ell ')}{\lambda }\,d\Xi '(\lambda ) \nonumber \\ \end{aligned}$$

and the limits in $\ell$ and *n* are restricted to the condition: $\ell \notin L'$ and $-\ell \notin L'$, $L'$ being the support of $\Xi '$.

### Remark 22

After a suitable translation of $\Xi _n$ and $\Xi$, one can assume that $0 \notin L$ and then one can take $\lambda ^* = 0$. The choice of $\lambda ^*$ does not change the structure of the proof of the theorem: replacing $\lambda ^*$ by 0 may slightly simplify its reading. In (18), the condition $\ell \notin L$, $-\ell \notin L$ ensures that the boundary terms in the integral defining $h_{n,\ell }$ does not perturb the existence of the limit of $h_{n,\ell }$ when $n \rightarrow \infty$. It may be possible to avoid this technicality by considering upper and lower limits in *n*.

### Proof

We have to show the following:

- The existence of the limits defining $S_n$ and *S*, and the fact that they are meromorphic, with simple poles at $L_n$ and *L*, respectively.
- The convergence of $S_n$ towards $S + {\bar{\gamma }}h$, uniformly in compacts, for the distance *d*.
- The assumptions (21)–(24), which should be satisfied by $\Xi '_n$ and $\Xi '$.

We will successively show these three statements: the most difficult one is the convergence of $S_n$ towards $S + {\bar{\gamma }}h$.

*Existence and properties of the limits defining* $S_n$ *and* *S*: Let $n \ge 1$, large enough in order to ensure that $\lambda ^* \notin L_n$, $1< \ell _0 < \ell \le \infty$, and let *z* be a complex number with modulus smaller than $\ell _0/2$. Let $k_{n, \ell _0}$ be the smallest index *k* (if it exists) such that $\lambda _{n,k} > \ell _0$, where $\lambda _{n,k}$ is (if it exists) the *k*th point of $L_n$ after $\lambda ^*$ for $k \ge 1$, and the $(1-k)$th point of $L_n$ before $\lambda ^*$ for $k \le 0$. Similarly, let $K_{n, \ell }-1$ be the largest index *k* (if it exists) such that $\lambda _{n,k} \le \ell$. For $\ell = \infty$ and $L_n$ bounded from above, we get that $K_{n, \infty }-1$ is the largest index *k* (if it exists) such that $\lambda _{n,k}$ exists, i.e. the index of the largest point of $L_n$. For $\ell = \infty$ and $L_n$ not bounded from above, we have $K_{n, \infty } = \infty$. If for $m \in {\mathbb {Z}}$,

$$\begin{aligned} \Delta _{n,m} := \mathbf {1}_{m \ge 0} \sum _{k=0}^{m-1} \gamma _{n,k} - \mathbf {1}_{m < 0} \sum _{k=m}^{-1} \gamma _{n,k} - {\bar{\gamma }} \,m, \end{aligned}$$

then, in the case where $k_{n, \ell _0}$ and $K_{n, \ell }$ are well-defined and $k_{n, \ell _0} < K_{n, \ell }$:

$$\begin{aligned}&\int _{(\ell _0,\ell ]} \frac{1}{\lambda -z}\,d\Lambda _n(\lambda ) \\&\quad = \sum _{k_{n,\ell _0} \le k< K_{n,\ell } } \frac{ \gamma _{n,k}}{\lambda _{n,k} - z} = {\bar{\gamma }} \sum _{k_{n,\ell _0} \le k< K_{n,\ell }} \frac{1}{\lambda _{n,k} - z} \\&\qquad + \, \sum _{k_{n,\ell _0} \le k< K_{n,\ell }} \frac{\Delta _{n,k+1} - \Delta _{n,k}}{\lambda _{n,k} - z} = {\bar{\gamma }} \left( \sum _{k_{n,\ell _0} \le k< K_{n,\ell }} \frac{1}{\lambda _{n,k} - z} \right) + \frac{\Delta _{n,K_{n,\ell }}}{ \lambda _{n, K_{n,\ell }-1} - z } \\&\qquad -\, \frac{\Delta _{n, k_{n,\ell _0}}}{ \lambda _{n, k_{n,\ell _0}} - z } + \sum _{k_{n,\ell _0} +1 \le k < K_{n,\ell } } \Delta _{n,k} \left( \frac{1}{\lambda _{n,k-1} -z} - \frac{1}{\lambda _{n,k} -z} \right) , \end{aligned}$$

which implies

$$\begin{aligned}&\int _{(\ell _0,\ell ]} \frac{1}{\lambda -z}\,d\Lambda _n(\lambda ) - {\bar{\gamma }} \int _{(\ell _0, \ell ]} \, \frac{d\Xi _n(\lambda )}{\lambda } \\&\quad = {\bar{\gamma }} z \left( \sum _{k_{n,\ell _0} \le k< K_{n,\ell }} \frac{1}{\lambda _{n,k} (\lambda _{n,k} - z)} \right) + \frac{\Delta _{n,K_{n,\ell }}}{ \lambda _{n, K_{n,\ell } - 1} - z } \\&\qquad -\,\frac{\Delta _{n,k_{n,\ell _0}}}{ \lambda _{n, k_{n,\ell _0}} - z } + \sum _{k_{n,\ell _0} +1 \le k < K_{n,\ell } } \Delta _{n,k} \left( \frac{\lambda _{n,k} - \lambda _{n,k-1}}{(\lambda _{n,k-1} -z) (\lambda _{n,k} -z)} \right) . \end{aligned}$$

Note that in case where $k_{n,\ell _0}$ or $K_{n,\ell }$ is not well-defined, and in case where $k_{n,\ell _0} \ge K_{n,\ell }$, the left-hand side is zero, since $L_n$ has no point in the interval $(\ell _0, \ell ]$. Let us now check that for $\ell$ going to infinity, this quantity converges, uniformly in $\{z \in {\mathbb {C}}, |z| < \ell _0/2\}$, to the function $T_{n, \ell _0}$, holomorphic on this open set, and given by

25 $$\begin{aligned} T_{n, \ell _0}(z)&= {\bar{\gamma }} z \left( \sum _{k \ge k_{n,\ell _0} } \frac{1}{\lambda _{n,k} (\lambda _{n,k} - z)} \right) + \frac{\Delta _{n,K_{n,\infty }}}{ \lambda _{n, K_{n,\infty }-1} - z } - \frac{\Delta _{n,k_{n,\ell _0}}}{ \lambda _{n, k_{n,\ell _0}} - z } \nonumber \\&\quad +\,\sum _{ k \ge k_{n,\ell _0}+1} \Delta _{n,k} \left( \frac{\lambda _{n,k} - \lambda _{n,k-1}}{(\lambda _{n,k-1} -z) (\lambda _{n,k} -z)} \right) , \end{aligned}$$

if $L_n$ has at least one point in $(\ell _0, \infty )$, and $T_{n, \ell _0} (z) = 0$ otherwise. In the formula above, when $L_n$ is not bounded from above, and then $K_{n,\infty } = \infty$, we let, by convention:

$$\begin{aligned} \frac{\Delta _{n,K_{n,\infty }}}{ \lambda _{n, K_{n,\infty }-1} - z } := 0. \end{aligned}$$

In order to prove this convergence, it is sufficient to check, in the case $L_n \cap (\ell _0, \infty ) \ne \emptyset$, the uniform convergence

$$\begin{aligned} \frac{\Delta _{n,K_{n,\ell }}}{ \lambda _{n, K_{n,\ell } - 1} - z } \underset{\ell \rightarrow \infty }{\longrightarrow } \frac{\Delta _{n,K_{n,\infty }}}{ \lambda _{n, K_{n,\infty } - 1} - z }, \end{aligned}$$

for $|z| \le \ell _0/2$, and the fact that

26 $$\begin{aligned} \sup _{z \in {\mathbb {C}}, |z| \le \ell _0/2} \left( \sum _{k \ge k_{n,\ell _0} } \frac{1}{|\lambda _{n,k}||\lambda _{n,k} - z|}+ \sum _{ k \ge k_{n,\ell _0}+1} |\Delta _{n,k}| \left( \frac{|\lambda _{n,k} - \lambda _{n,k-1}|}{|\lambda _{n,k-1} -z| \, |\lambda _{n,k} -z|} \right) \right) < \infty . \end{aligned}$$

The first statement is immediate if $L_n$ is bounded from above. If $L_n$ is unbounded from above, let us remark that for $k \ge k_{n, \ell _0}$, $|z| \le \ell _0/2$, one has $|\lambda _{n,k} - z| \ge \lambda _{n,k}/2$, and then it is sufficient to show:

27 $$\begin{aligned} \frac{\Delta _{n,K_{n,\ell }}}{ \lambda _{n, K_{n,\ell } - 1}} \underset{\ell \rightarrow \infty }{\longrightarrow } 0. \end{aligned}$$

Similarly, the statement (26) is implied by:

28 $$\begin{aligned} \sum _{k \ge k_{n,\ell _0} } \frac{1}{\lambda _{n,k}^2}+ \sum _{ k \ge k_{n,\ell _0}+1} \frac{ |\Delta _{n,k}| (\lambda _{n,k} - \lambda _{n,k-1})}{\lambda _{n,k-1} \, \lambda _{n,k}} < \infty . \end{aligned}$$

In order to prove (27), let us first use the majorization (16), which implies, for all $\ell > 2$,

$$\begin{aligned} \Xi _n ([2, \ell ]) \le \ell ^{1 + \alpha } \, \int _{2}^{\ell } \frac{d\Xi _n(\lambda )}{\lambda ^{1 + \alpha }} \le \ell ^{1 + \alpha } \, \int _{{\mathbb {R}}}\frac{{\mathbf {1}}(|\lambda |>1)}{|\lambda |^{1+\alpha }}\, d\Xi _n(\lambda ) \le \tau _1 \, \ell ^{1 + \alpha }, \end{aligned}$$

and then

$$\begin{aligned} \Xi _n ([\lambda ^*, \ell ]) \le \tau \, \ell ^{1+ \alpha } \end{aligned}$$

where

29 $$\begin{aligned} \tau := \tau _1 + \Xi _n ([\lambda ^* \wedge 2, 2]). \end{aligned}$$

We deduce, for $k \ge 1$ large enough in order to insure that $\lambda _{n,k} > 2$,

$$\begin{aligned} k = \Xi _n ([\lambda ^*, \lambda _{n,k}]) \le \tau \, \lambda _{n,k}^{1+\alpha } \end{aligned}$$

and then

30 $$\begin{aligned} \lambda _{n,k} \ge (k/ \tau )^{1/(1+\alpha )}. \end{aligned}$$

By using (20), this inequality implies:

$$\begin{aligned} \frac{\Delta _{n,k+1}}{ \lambda _{n,k}} \le c (k+1)^{\alpha '} (k/\tau )^{-1/(1+\alpha )}, \end{aligned}$$

which tends to zero when *k* goes to infinity, since $\alpha ' < 1/(1+\alpha )$ by assumption. Therefore, we have (27).

Moreover, the left-hand side of (28) is given by

$$\begin{aligned}&\int _{{\mathbb {R}}} \frac{{\mathbf {1}}(|\lambda |>\ell _0)}{|\lambda |^{2}}\,d\Xi _n(\lambda ) + \sum _{ k \ge k_{n,\ell _0}+1} |\Delta _{n,k}| \, \left( \frac{1}{\lambda _{n,k-1}} - \frac{1}{\lambda _{n,k}} \right) \\&\quad \le \int _{{\mathbb {R}}}\frac{{\mathbf {1}}(|\lambda |>\ell _0)}{|\lambda |^{1+\alpha }}\,d\Xi _n(\lambda ) + c \, \sum _{ k \ge k_{n,\ell _0}+1} |k|^{\alpha '} \, \left( \frac{1}{\lambda _{n,k-1}} - \frac{1}{\lambda _{n,k}} \right) \\&\quad \le \tau _{\ell _0} + c \left( \frac{|k_{n,\ell _0}+1|^{\alpha '}}{\lambda _{n,k_{n,\ell _0}}} + \sum _{ k \ge k_{n,\ell _0}+1} \frac{(|k+1|^{\alpha '} - |k|^{\alpha '})}{\lambda _{n,k}} \right) . \end{aligned}$$

If $L_n$ is bounded from above, the finiteness of this quantity is obvious. Otherwise, we know that for *k* large enough, $(|k+1|^{\alpha '} - |k|^{\alpha '})$ is bounded by a constant times $k^{\alpha ' - 1}$, and $\lambda _{k, n}$ dominates $k^{1/(1+\alpha )}$. Hence, it is sufficient to check the finiteness of the following expression:

$$\begin{aligned} \sum _{k = 1}^{\infty } k^{\alpha ' -1} k^{-1/(1+\alpha )}, \end{aligned}$$

which is satisfied since by assumption,

$$\begin{aligned} \alpha ' - 1 - \frac{1}{1 + \alpha } < -1. \end{aligned}$$

We have now proven:

31 $$\begin{aligned} \int _{(\ell _0,\ell ]} \frac{1}{\lambda -z}\,d\Lambda _n(\lambda ) - {\bar{\gamma }} \int _{(\ell _0, \ell ]} \, \frac{d\Xi _n(\lambda )}{\lambda } \underset{\ell \rightarrow \infty }{\longrightarrow } T_{n, \ell _0}(z), \end{aligned}$$

uniformly on the set $\{z \in {\mathbb {C}}, |z| < \ell _0/2\}$, where the holomorphic function $T_{n, \ell _0}$ is given by the formula (25).

Similarly, there exists an holomorphic function $U_{n, \ell _0}$ on $\{z \in {\mathbb {C}}, |z| < \ell _0/2\}$, such that uniformly on this set,

32 $$\begin{aligned} \int _{[-\ell , -\ell _0)} \frac{1}{\lambda -z}\,d\Lambda _n(\lambda ) - {\bar{\gamma }} \int _{[-\ell , -\ell _0)} \, \frac{d\Xi _n(\lambda )}{\lambda } \underset{\ell \rightarrow \infty }{\longrightarrow } U_{n, \ell _0}(z). \end{aligned}$$

The function $U_{n, \ell _0}$ can be explicitly described by a formula similar to (25) (we omit the detail of this formula). By combining (17), (31) and (32), one deduces the following uniform convergence on $\{z \in {\mathbb {C}}, |z| < \ell _0/2\}$:

$$\begin{aligned} \int _{[-\ell , \ell ] {\backslash }[-\ell _0, \ell _0]} \frac{1}{\lambda -z}\,d\Lambda _n(\lambda ) \underset{\ell \rightarrow \infty }{\longrightarrow } T_{n, \ell _0}(z) + U_{n, \ell _0}(z) + {\bar{\gamma }} h_{n, \ell _0}. \end{aligned}$$

One deduces, by using Lemma 18, that

$$\begin{aligned}&\int _{[-\ell , \ell ]} \frac{1}{\lambda -z}\,d\Lambda _n(\lambda ) \underset{\ell \rightarrow \infty }{\longrightarrow } T_{n, \ell _0}(z) + U_{n, \ell _0}(z) + {\bar{\gamma }} h_{n, \ell _0} \\&\quad + \int _{[-\ell _0, \ell _0]} \frac{1}{\lambda -z}\,d\Lambda _n(\lambda ) =: S_{n, \ell _0} (z), \end{aligned}$$

uniformly on any compact subset of $\{z \in {\mathbb {C}}, |z| < \ell _0/2\}$, for the distance *d* on the Riemann sphere. One checks immediately that the poles of $S_{n, \ell _0}$ with modulus smaller than or equal to $\ell _0/2$ are exactly the points of $L_n$ satisfying the same condition. Moreover, the convergence just above implies that for $\ell _1> \ell _0 > 1$, the meromorphic functions $S_{n, \ell _0}$ and $S_{n, \ell _1}$ coincide on $\{z \in {\mathbb {C}}, |z| < \ell _0/2\}$: hence, there exists a meromorphic function $S_n$ on ${\mathbb {C}}$, such that for all $\ell _0 > 1$, the restriction of $S_n$ to $\{z \in {\mathbb {C}}, |z| < \ell _0/2\}$ is equal to $S_{n, \ell _0}$. The poles of $S_n$ are exactly the points of $L_n$, and one has, uniformly on all compact sets of ${\mathbb {C}}$ and for the distance *d*,

$$\begin{aligned} \int _{[-\ell , \ell ]} \frac{1}{\lambda -z}\,d\Lambda _n(\lambda ) \underset{\ell \rightarrow \infty }{\longrightarrow } S_n(z). \end{aligned}$$

In particular, the convergence holds pointwise for all $z \notin L_n$.

In an exactly similar way, one can prove that uniformly on compact sets of ${\mathbb {C}}$, for the distance *d*,

$$\begin{aligned} \int _{[-\ell , \ell ]} \frac{1}{\lambda -z}\,d\Lambda (\lambda ) \underset{\ell \rightarrow \infty }{\longrightarrow } S(z) \end{aligned}$$

where for all $\ell _0 > 1$,

$$\begin{aligned} S(z) := T_{\ell _0}(z) + U_{\ell _0}(z) + {\bar{\gamma }} h_{\ell _0} + \int _{[-\ell _0, \ell _0]} \frac{1}{\lambda -z}\,d\Lambda (\lambda ), \end{aligned}$$

on the set $\{z \in {\mathbb {C}}, |z|< \ell _0/2\}$, $T_{\ell _0}$ and $U_{\ell _0}$ being defined by the same formulas as $T_{n,\ell _0}$ and $U_{n,\ell _0}$, except than one removes all the indices *n*. In order to show this convergence, it is sufficient to check that the assumptions (16) and (20) are satisfied if the indices *n* are removed. For (20), it is an immediate consequence of the convergence (19), since the constant *c* does not depend on *n*. For (16), let us first observe that for all $\ell > 1$, and for any continuous function $\Phi$ with compact support, such that for all $\lambda \in {\mathbb {R}}$,

$$\begin{aligned} \Phi (\lambda ) \le \frac{{\mathbf {1}}(|\lambda | > \ell )}{|\lambda |^{1+\alpha }}, \end{aligned}$$

one has for all $n \ge 1$,

$$\begin{aligned} \int _{{\mathbb {R}}} \Phi (\lambda ) d\Xi _n(\lambda ) \le \int _{{\mathbb {R}}}\frac{{\mathbf {1}}(|\lambda |>\ell )}{|\lambda |^{1+\alpha }} \,d\Xi _n(\lambda ) \le \tau _\ell . \end{aligned}$$

Since $\Xi _n$ converges weakly to $\Xi$ when *n* goes to infinity, one deduces:

$$\begin{aligned} \int _{{\mathbb {R}}} \Phi (\lambda ) d\Xi (\lambda ) \le \tau _{\ell }. \end{aligned}$$

By taking $\Phi$ increasing to

$$\begin{aligned} \lambda \mapsto {\mathbf {1}}(|\lambda | > \ell )/ |\lambda |^{1+\alpha }, \end{aligned}$$

one obtains

$$\begin{aligned} \int _{{\mathbb {R}}}\frac{{\mathbf {1}}(|\lambda |>\ell )}{|\lambda |^{1+\alpha }} \,d\Xi (\lambda ) \le \tau _\ell , \end{aligned}$$

i.e. the equivalent of (16) for the measure $\Xi$.

*Convergence of* $S_n$ *towards* $S + {\bar{\gamma }} h$: Once the existence of the functions $S_n$ and *S* is ensured, it remains to prove the convergence of $S_n$ towards $S+ {\bar{\gamma }} h$, uniformly on compact sets for the distance *d*. In order to check this convergence, it is sufficient to prove that for all $\ell > 1$, there exists $\ell _0 > \ell$ such that uniformly on any compact set of $\{z \in {\mathbb {C}}, |z| < \ell _0/2\}$,

$$\begin{aligned}&T_{n, \ell _0}(z) + U_{n, \ell _0}(z) + {\bar{\gamma }} h_{n, \ell _0} + \int _{[-\ell _0, \ell _0]} \frac{1}{\lambda -z}\,d\Lambda _n(\lambda ) \\&\quad \underset{n \rightarrow \infty }{\longrightarrow } T_{\ell _0}(z) + U_{\ell _0}(z) + {\bar{\gamma }} (h_{ \ell _0} + h ) + \int _{[-\ell _0, \ell _0]} \frac{1}{\lambda -z}\,d\Lambda (\lambda ). \end{aligned}$$

In fact, we will prove this convergence for any $\ell _0 >2$ such that $\ell _0$ and $-\ell _0$ are not in *L*, and then not in $L_n$ for *n* large enough. By Lemma 18, it is sufficient to check for such an $\ell _0$:

33 $$\begin{aligned}&h_{n, \ell _0} \underset{n \rightarrow \infty }{\longrightarrow } h_{\ell _0} + h, \end{aligned}$$

34 $$\begin{aligned}&\int _{[-\ell _0, \ell _0]} \frac{1}{\lambda -z}\,d\Lambda _n(\lambda ) \underset{n \rightarrow \infty }{\longrightarrow } \int _{[-\ell _0, \ell _0]} \frac{1}{\lambda -z}\,d\Lambda (\lambda ), \end{aligned}$$

uniformly on $\{z \in {\mathbb {C}}, |z| < \ell _0/2\}$ for the distance *d*,

35 $$\begin{aligned} T_{n, \ell _0}(z) \underset{n \rightarrow \infty }{\longrightarrow } T_{\ell _0}(z), \end{aligned}$$

uniformly on $\{z \in {\mathbb {C}}, |z| < \ell _0/2\}$, and

36 $$\begin{aligned} U_{n, \ell _0}(z) \underset{n \rightarrow \infty }{\longrightarrow } U_{\ell _0}(z), \end{aligned}$$

also uniformly on $\{z \in {\mathbb {C}}, |z| < \ell _0/2\}$. Since the proof of (36) is exactly similar to the proof of (35), we will omit it and we will then show successively (33)–(35). $\square$

### Proof of 33

For all $\ell _1 > \ell _0$ such that $-\ell _1$ and $\ell _1$ are not in *L*, one has

$$\begin{aligned} h_{n, \ell _0} - h_{n, \ell _1} = \int _{{\mathbb {R}}}\frac{{\mathbf {1}}(\ell _0<|\lambda | \le \ell _1)}{\lambda }\,d\Xi _n(\lambda ) \end{aligned}$$

and

$$\begin{aligned} h_{\ell _0} - h_{\ell _1} = \int _{{\mathbb {R}}}\frac{{\mathbf {1}}(\ell _0<|\lambda | \le \ell _1)}{\lambda }\,d\Xi (\lambda ). \end{aligned}$$

Now, $-\ell _1, -\ell _0, \ell _0, \ell _1$ are not in the support of $\Xi$, and since $\Xi$ is a discrete measure, there is a neighborhood of $\{-\ell _1, -\ell _0, \ell _0, \ell _1\}$ which does not charge $\Xi$. One deduces that there exist two functions $\Phi$ and $\Psi$ from ${\mathbb {R}}$ to ${\mathbb {R}}_+$, continuous with compact support, such that for all $\lambda \in {\mathbb {R}}$,

$$\begin{aligned} \Phi (\lambda ) \le \frac{{\mathbf {1}}(\ell _0<|\lambda | \le \ell _1)}{\lambda } \le \Psi (\lambda ) \end{aligned}$$

and

$$\begin{aligned} \int _{{\mathbb {R}}} \Phi (\lambda ) \, d\Xi (\lambda ) = h_{\ell _0} - h_{\ell _1} = \int _{{\mathbb {R}}} \Psi (\lambda ) \, d\Xi (\lambda ). \end{aligned}$$

Since $\Xi _n$ tends weakly to $\Xi$ when *n* goes to infinity, one deduces that

$$\begin{aligned} \int _{{\mathbb {R}}} \Phi (\lambda ) \, d\Xi _n(\lambda ) \underset{n \rightarrow \infty }{\longrightarrow } \int _{{\mathbb {R}}} \Phi (\lambda ) \, d\Xi (\lambda ) = h_{\ell _0} - h_{\ell _1} \end{aligned}$$

and similarly,

$$\begin{aligned} \int _{{\mathbb {R}}} \Psi (\lambda ) \, d\Xi _n(\lambda ) \underset{n \rightarrow \infty }{\longrightarrow } h_{\ell _0} - h_{\ell _1}. \end{aligned}$$

By the squeeze theorem, one deduces

$$\begin{aligned} h_{n, \ell _0} - h_{n, \ell _1} = \int _{{\mathbb {R}}}\frac{{\mathbf {1}}(\ell _0<|\lambda | \le \ell _1)}{\lambda }\,d\Xi _n(\lambda )\underset{n \rightarrow \infty }{\longrightarrow } h_{\ell _0} - h_{\ell _1}. \end{aligned}$$

Hence,

$$\begin{aligned} \underset{n \rightarrow \infty }{\lim } h_{n, \ell _0} - \underset{n \rightarrow \infty }{\lim } h_{n, \ell _1} = h_{\ell _0} - h_{\ell _1}. \end{aligned}$$

where, by assumption, the two limits in the left-hand side are well-defined. By (18), one deduces, by taking $\ell _1 \rightarrow \infty$,

$$\begin{aligned} \underset{n \rightarrow \infty }{\lim } h_{n, \ell _0} - h = h_{\ell _0}, \end{aligned}$$

which proves (33). $$\square $$

### Proof of 34

Let us now check the following properties, available for all $k \in {\mathbb {Z}}$:

- If $\lambda _k$ is well-defined, then $\lambda _{n,k}$ is well-defined for all *n* large enough and tends to $\lambda _{k}$ when *n* goes to infinity.
- If $\lambda _k$ is not well-defined, then for all $A > 0$, there are finitely many indices *n* such that $\lambda _{n,k}$ is well-defined and in the interval $[-A,A]$.

By symmetry, we can assume that $k \ge 1$. We know that $\lambda ^*$ is not in *L*, and then for $\epsilon > 0$ small enough,

37 $$\begin{aligned} L \cap [\lambda ^* - 3 \epsilon , \lambda ^* + 3 \epsilon ] = \emptyset , \end{aligned}$$

Let us fix $\epsilon > 0$ satisfying this property. Since $\Xi _n$ tends locally weakly to $\Xi$, we deduce that for *n* large enough,

38 $$\begin{aligned} L_n \cap [\lambda ^* - 2 \epsilon , \lambda ^* + 2\epsilon ] = \emptyset , \end{aligned}$$

which implies that $\lambda _k \ge \lambda _1 > \lambda ^* + 2\epsilon$. Now, let $\Phi$ and $\Psi$ be two continuous functions with compact support, such that:

- For $$\lambda \le \lambda ^* - \epsilon $$,
  $$\begin{aligned} \Phi (\lambda ) = \Psi (\lambda ) = 0. \end{aligned}$$
- For $$\lambda ^* - \epsilon \le \lambda \le \lambda ^* + \epsilon $$,
  $$\begin{aligned} 0 \le \Phi (\lambda ) = \Psi (\lambda ) \le 1. \end{aligned}$$
- For $$\lambda ^* + \epsilon \le \lambda \le \lambda _k - \epsilon $$,
  $$\begin{aligned} \Phi (\lambda ) = \Psi (\lambda ) = 1 \end{aligned}$$
  (recall that $$\lambda ^* + \epsilon < \lambda _k - \epsilon $$).
- For $$\lambda _k - \epsilon \le \lambda \le \lambda _k$$,
  $$\begin{aligned} 0 \le \Phi (\lambda ) \le \Psi (\lambda ) = 1. \end{aligned}$$
- For $$\lambda _k \le \lambda \le \lambda _k+ \epsilon $$,
  $$\begin{aligned} 0 = \Phi (\lambda ) \le \Psi (\lambda ) \le 1. \end{aligned}$$
- For $$ \lambda \ge \lambda _k+ \epsilon $$,
  $$\begin{aligned} \Phi (\lambda ) = \Psi (\lambda ) = 0. \end{aligned}$$

By using (37), we deduce

$$\begin{aligned} \int _{{\mathbb {R}}} \Phi (\lambda ) d \Xi (\lambda ) \le \Xi ([\lambda ^* - \epsilon , \lambda _k)) = \Xi ([\lambda ^*, \lambda _k)) = k-1 \end{aligned}$$

and

$$\begin{aligned} \int _{{\mathbb {R}}} \Psi (\lambda ) d \Xi (\lambda ) \ge \Xi ([\lambda ^* + \epsilon , \lambda _k]) = \Xi ([\lambda ^*, \lambda _k]) = k. \end{aligned}$$

Hence, for *n* large enough,

$$\begin{aligned} \int _{{\mathbb {R}}} \Phi (\lambda ) d \Xi _n(\lambda ) \le k - 1/2 \end{aligned}$$

and

$$\begin{aligned} \int _{{\mathbb {R}}} \Psi (\lambda ) d \Xi _n(\lambda ) \ge k - 1/2, \end{aligned}$$

which implies

$$\begin{aligned} \Xi _n ([\lambda ^*, \lambda _k - \epsilon )) = \Xi _n ([\lambda ^*+ \epsilon , \lambda _k - \epsilon )) \le \int _{{\mathbb {R}}} \Phi (\lambda ) d \Xi _n(\lambda ) \le k - 1/2 \end{aligned}$$

and

$$\begin{aligned} \Xi _n ([\lambda ^*, \lambda _k + \epsilon )) = \Xi _n ([\lambda ^*- \epsilon , \lambda _k + \epsilon ]) \ge \int _{{\mathbb {R}}} \Psi (\lambda ) d \Xi _n(\lambda ) \ge k - 1/2. \end{aligned}$$

Therefore, for *n* large enough the point $\lambda _{n,k}$ is well-defined and between $\lambda _{k} - \epsilon$ and $\lambda _{k} + \epsilon$. Since $\epsilon$ and be taken arbitrarily small, we have proven the convergence claimed above in the case where $\lambda _k$ is well-defined. If $\lambda _k$ is not well-defined, let us choose $\epsilon > 0$ satisfying (37), and $A > |\lambda ^*|$. Let $\Phi$ be a continuous function with compact support, such that:

- For all $$\lambda \in {\mathbb {R}}$$, $$\Phi (\lambda ) \in [0,1]$$.
- For all $$\lambda \in [\lambda ^*, A]$$, $$\Phi (\lambda ) = 1$$.
- For all $$\lambda \notin (\lambda ^*-\epsilon , A + \epsilon )$$, $$\Phi (\lambda ) = 0$$.

Since $\lambda _k$ is not well-defined,

$$\begin{aligned} \int _{{\mathbb {R}}} \Phi (\lambda ) d \Xi (\lambda ) \le \Xi ([\lambda ^*- \epsilon , A+ \epsilon ]) = \Xi ([\lambda ^*, A +\epsilon ]) \le \Xi ([\lambda ^*, \infty )) \le k-1, \end{aligned}$$

and then for *n* large enough,

$$\begin{aligned} \Xi _n([\lambda ^*, A]) \le \int _{{\mathbb {R}}} \Phi (\lambda ) d \Xi _n(\lambda ) \le k-1/2, \end{aligned}$$

which implies that $\lambda _{n,k}$ cannot be well-defined and smaller than or equal to *A*. This proves the second claim. Let us now go back to the proof of (34). If $L \cap [-\ell _0, \ell _0] = \emptyset$, then $L \cap [-\ell _0- \epsilon , \ell _0+ \epsilon ] = \emptyset$ for some $\epsilon > 0$. Hence, there exists a nonnegative, continuous function with compact support $\Phi$ such that $\Phi (\lambda ) = 1$ for all $\lambda \in [-\ell _0, \ell _0]$, and

$$\begin{aligned} \int _{{\mathbb {R}}} \Phi (\lambda ) d \Xi (\lambda ) = 0, \end{aligned}$$

which implies, for *n* large enough,

$$\begin{aligned} \Xi ([-\ell _0, \ell _0]) \le \int _{{\mathbb {R}}} \Phi (\lambda ) d \Xi _n(\lambda ) \le 1/2, \end{aligned}$$

i.e. $L_n \cap [-\ell _0, \ell _0] = \emptyset$. Hence, for *n* large enough, the two expressions involved in (34) are identically zero. If $L \cap [-\ell _0, \ell _0] \ne \emptyset$, let $k_1$ and $k_2$ be the smallest and the largest indices *k* such that $\lambda _k \in (-\ell _0, \ell _0)$. Since $\lambda _{n,k_1}$ and $\lambda _{n,k_2}$ converge respectively to $\lambda _{k_1}$ and $\lambda _{k_2}$ when *n* goes to infinity, one has $\lambda _{n,k_1}$ and $\lambda _{n,k_2}$ in the interval $(-\ell _0, \ell _0)$ for *n* large enough. On the other hand, $\lambda _{k_2+1}$ is either strictly larger than $\ell _0$ (strictly because by assumption, $\ell _0 \notin L$), or not well-defined. In both cases, there are only finitely many indices *n* such that $\lambda _{n,k_2+1} \le \ell _0$. Similarly, by using the fact that $-\ell _0 \notin L$, one checks that there are finitely many indices *n* such that $\lambda _{n,k_1-1} \ge -\ell _0$. Hence, for *n* large enough, the indices *k* such that $\lambda _{n,k} \in [-\ell _0, \ell _0]$ are exactly the integers between $k_1$ and $k_2$, which implies

$$\begin{aligned} \int _{[-\ell _0, \ell _0]} \frac{1}{\lambda -z}\,d\Lambda _n(\lambda ) = \sum _{k= k_1}^{k_2} \frac{\gamma _{n,k}}{\lambda _{n,k} - z}, \end{aligned}$$

whereas

$$\begin{aligned} \int _{[-\ell _0, \ell _0]} \frac{1}{\lambda -z}\,d\Lambda (\lambda ) = \sum _{k= k_1}^{k_2} \frac{\gamma _{k}}{\lambda _{k} - z}. \end{aligned}$$

We have shown that for all *k* between $k_1$ and $k_2$, $\lambda _{n,k}$ tends to $\lambda _k$ when *n* goes to infinity and by assumption, $\gamma _{n,k}$ tends to $\gamma _k$. Moreover, the numbers $\lambda _k$ are all distincts, and by assumption, $\gamma _k \ne 0$ for all *k*. Hence, one can apply Lemma 20 to deduce (34). $\square$

### Proof of 35

If $L \cap (\ell _0, \infty ) = \emptyset$, this statement can be deduced from the following convergences, uniformly on $\{z \in {\mathbb {C}}, |z| < \ell _0/2\}$:

39 $$\begin{aligned}&\mathbf {1}_{L_n \cap (\ell _0, \infty ) \ne \emptyset } \, \sum _{k \ge k_{n,\ell _0} } \frac{1}{\lambda _{n,k} (\lambda _{n,k} - z)} \underset{n \rightarrow \infty }{\longrightarrow } 0, \end{aligned}$$

40 $$\begin{aligned}&\mathbf {1}_{L_n \cap (\ell _0, \infty ) \ne \emptyset } \, \frac{\Delta _{n,K_{n,\infty }}}{ \lambda _{n, K_{n,\infty }-1} - z } \underset{n \rightarrow \infty }{\longrightarrow } 0, \end{aligned}$$

41 $$\begin{aligned}&\mathbf {1}_{L_n \cap (\ell _0, \infty ) \ne \emptyset } \, \frac{\Delta _{n,k_{n,\ell _0}}}{ \lambda _{n, k_{n,\ell _0}} - z } \underset{n \rightarrow \infty }{\longrightarrow } 0 \end{aligned}$$

and

42 $$\begin{aligned} \mathbf {1}_{L_n \cap (\ell _0, \infty ) \ne \emptyset } \, \sum _{ k \ge k_{n,\ell _0}+1} \Delta _{n,k} \left( \frac{\lambda _{n,k} - \lambda _{n,k-1}}{(\lambda _{n,k-1} -z) (\lambda _{n,k} -z)} \right) \underset{n \rightarrow \infty }{\longrightarrow } 0. \end{aligned}$$

If $L \cap (\ell _0, \infty ) \ne \emptyset$, then we have proven previously that $\lambda _{n,k_{\ell _0}}$ is well-defined for *n* large enough and converges to $\lambda _{k_{\ell _0}} > \ell _0$ when *n* goes to infinity: in particular, $\lambda _{n,k_{\ell _0}} > \ell _0$ for *n* large enough. Moreover, one of the two following cases occurs:

- If $\lambda _{k_{\ell _0} - 1}$ is well-defined, then it is strictly smaller than $\ell _0$ (strictly because $\ell _0$ is, by assumption, not in *L*), and then $\lambda _{n,k_{\ell _0} - 1}$ is, for *n* large enough, well-defined and strictly smaller than $\ell _0$.
- If $\lambda _{k_{\ell _0} - 1}$ is not well-defined, and if $A > 0$, then for *n* large enough, $\lambda _{n,k_{\ell _0} - 1}$ is not well-defined or has an absolute value strictly greater than *A*. By taking $A = \lambda _{k_{\ell _0}} + 1$, one deduces that for *n* large enough, $\lambda _{n,k_{\ell _0} - 1}$ is not-well defined, strictly smaller than $- \lambda _{k_{\ell _0}} - 1$ or strictly larger than $\lambda _{k_{\ell _0}} + 1$. This last case is impossible for *n* large enough, since $\lambda _{n,k_{\ell _0} - 1}$ is smaller than $\lambda _{n,k_{\ell _0}}$, which tends to $\lambda _{k_{\ell _0}}$. Hence, there are finitely many indices *n* such that $\lambda _{n,k_{\ell _0} - 1}$ is well-defined and larger than $- \lambda _{k_{\ell _0}} - 1$, and a fortiori, larger than or equal to $\ell _0$.

All this discussion implies easily that for *n* large enough, $k_{n,\ell _0} = k_{\ell _0}$, and then it is sufficient to prove the uniform convergences on $\{z \in {\mathbb {C}}, |z| < \ell _0/2\}$:

43 $$\begin{aligned}&\sum _{k \ge k_{\ell _0} } \frac{1}{\lambda _{n,k} (\lambda _{n,k} - z)} \underset{n \rightarrow \infty }{\longrightarrow } \sum _{k \ge k_{\ell _0} } \frac{1}{\lambda _{k} (\lambda _{k} - z)}, \end{aligned}$$

44 $$\begin{aligned}&\frac{\Delta _{n,k_{\ell _0}}}{ \lambda _{n, k_{\ell _0}} - z } \underset{n \rightarrow \infty }{\longrightarrow } \frac{\Delta _{k_{\ell _0}}}{ \lambda _{ k_{\ell _0}} - z } \end{aligned}$$

and

45 $$\begin{aligned}&\frac{\Delta _{n,K_{n,\infty }}}{ \lambda _{n, K_{n,\infty }-1} - z } + \sum _{ k \ge k_{\ell _0}+1} \Delta _{n,k} \left( \frac{\lambda _{n,k} - \lambda _{n,k-1}}{(\lambda _{n,k-1} -z) (\lambda _{n,k} -z)} \right) \nonumber \\&\quad \underset{n \rightarrow \infty }{\longrightarrow } \frac{\Delta _{K_{\infty }}}{ \lambda _{ K_{\infty }-1} - z } +\sum _{ k \ge k_{\ell _0}+1} \Delta _{k} \left( \frac{\lambda _{k} - \lambda _{k-1}}{(\lambda _{k-1} -z) (\lambda _{k} -z)} \right) , \end{aligned}$$

with obvious notation.

Let us first prove (39). If $L \cap (\ell _0, \infty ) = \emptyset$, then $L \cap (\ell _0 - \epsilon , \infty ) = \emptyset$ for some $\epsilon > 0$ (recall that $\ell _0 \notin L$). Hence, for all $A > \ell _0$, and *n* large enough depending on *A*, $L_n \cap (\ell _0,A] = \emptyset$, which implies, for $|z| \le \ell _0/2$,

$$\begin{aligned} \left| \mathbf {1}_{L_n \cap (\ell _0, \infty ) \ne \emptyset } \, \sum _{k \ge k_{n,\ell _0} } \frac{1}{\lambda _{n,k} (\lambda _{n,k} - z)} \right|&\le 2 \int _{{\mathbb {R}}} \frac{{\mathbf {1}} (\lambda> \ell _0)}{\lambda ^2} \, d \Xi _n(\lambda ) \\&= 2 \int _{{\mathbb {R}}} \frac{{\mathbf {1}} (\lambda> A)}{\lambda ^2} \, d \Xi _n(\lambda ) \\&\le 2\int _{{\mathbb {R}}} \frac{{\mathbf {1}} (\lambda > A)}{\lambda ^{1+\alpha }} \, d \Xi _n(\lambda ) \le 2 \tau _A. \end{aligned}$$

By letting $n \rightarrow \infty$ and then $A \rightarrow \infty$, one deduces (39).

Let us prove (40) and (41). By using the estimates (29) and (30) proven above, one deduces that for

$$\begin{aligned} {\tilde{\tau }} := \tau _1 + \Xi ([\lambda ^* \wedge 2, 2]) + \sup _{n \ge 1} \Xi _n([\lambda ^* \wedge 2, 2]), \end{aligned}$$

one has, for any $k \ge 1$,

46 $$\begin{aligned} \lambda _{k} \ge (k/{\tilde{\tau }})^{1/(1+\alpha )} , \end{aligned}$$

if $\lambda _{k}> 2$, and uniformly in *n*,

47 $$\begin{aligned} \lambda _{k,n} \ge (k/{\tilde{\tau }})^{1/(1+\alpha )} , \end{aligned}$$

if $\lambda _{k,n} > 2$. Now, let us assume that $L \cap (\ell _0, \infty ) = \emptyset$ and $L_n \cap (\ell _0, \infty ) \ne \emptyset$. If *n* is large enough, then for any index *k* such that $\lambda _{n,k} > \ell _0$, one has also $\lambda _{n,k} > \lambda ^* \vee 2$, since $L \cap (\ell _0-\epsilon , (\lambda ^* \vee 2) + 1) = \emptyset$ for some $\epsilon > 0$, and $\Xi _{n} \rightarrow \Xi$. Hence, $k \ge 1$ and (47) is satisfied. By using this inequality and (20), one deduces, for $|z| \le \ell _0/2$,

$$\begin{aligned}&\left| \mathbf {1}_{L_n \cap (\ell _0, \infty ) \ne \emptyset } \, \frac{\Delta _{n,K_{n,\infty }}}{ \lambda _{n, K_{n,\infty }-1} - z } \right| + \left| \mathbf {1}_{L_n \cap (\ell _0, \infty ) \ne \emptyset } \, \frac{\Delta _{n,k_{n,\ell _0}}}{ \lambda _{n, k_{n,\ell _0}} - z }\right| \\&\quad \le \sup _{k \ge 1} \frac{2 c (k+1)^{\alpha '}}{(k/{\tilde{\tau }})^{1/(1+\alpha )} \vee \lambda _{n, k_{n,\ell _0}} } +\sup _{k \ge 1} \frac{2 c k^{\alpha '}}{(k/{\tilde{\tau }})^{1/(1+\alpha )} \vee \lambda _{n, k_{n,\ell _0}} } \\&\quad \le 4c (1+2^{\alpha '}) (1+{\tilde{\tau }})^{1/(1+\alpha )} \sup _{k \ge 1} \frac{k^{\alpha '}}{k^{1/(1+\alpha )} \vee \lambda _{n, k_{n,\ell _0}} } \\&\quad = 4c (1+2^{\alpha '}) (1+{\tilde{\tau }})^{1/(1+\alpha )}\sup _{k \ge 1} \frac{(k^{1/(1+ \alpha )})^{\alpha '(1+\alpha )}}{k^{1/(1+\alpha )} \vee \lambda _{n, k_{n,\ell _0}} } \\&\quad \le 4c (1+2^{\alpha '}) (1+{\tilde{\tau }})^{1/(1+\alpha )}\sup _{k \ge 1} (k^{1/(1+\alpha )} \vee \lambda _{n, k_{n,\ell _0}})^{\alpha '(1+\alpha ) - 1} \\&\quad \le 4c (1+2^{\alpha '}) (1+{\tilde{\tau }})^{1/(1+\alpha )}\lambda _{n, k_{n,\ell _0}}^{\alpha '(1+\alpha ) - 1}, \end{aligned}$$

where $\lambda _{n, k_{n,\ell _0}}$ is taken equal to $\infty$ for $L_n \cap (\ell _0, \infty ) = \emptyset$. Note that in the previous computation, the last inequality is a consequence of the inequality $\alpha '(1+\alpha ) - 1 <0$. Now, $\lambda _{n, k_{n,\ell _0}}$ tends to infinity with *n*, since for all $A > \ell _0$, one has $L_n \cap (\ell _0, A] = \emptyset$ for *n* large enough. Hence, we get (40) and (41).

Moreover, in case where $L \cap (\ell _0, \infty ) = \emptyset$, $L_n \cap (\ell _0, \infty ) \ne \emptyset$, *n* is large enough, and $|z| < \ell _0/2$, the left-hand side of (42) is smaller than or equal to:

$$\begin{aligned}&4 c \sum _{ k \ge k_{n,\ell _0}+1} |k|^{\alpha '} \left( \frac{\lambda _{n,k} - \lambda _{n,k-1}}{\lambda _{n,k-1} \lambda _{n,k} } \right) = 4 c \sum _{ k \ge k_{n,\ell _0}+1} |k|^{\alpha '} \left( \frac{1}{\lambda _{n,k-1}} -\frac{1}{\lambda _{n,k}} \right) \\&\quad = 4c \left( \frac{|k_{n, \ell _0}+1|^{\alpha '}}{\lambda _{n,k_{n, \ell _0}}} + \sum _{ k \ge k_{n,\ell _0}+1} \frac{|k+1|^{\alpha '} - |k|^{\alpha '}}{\lambda _{n,k}} \right) \\&\quad \le 4c \left( \frac{|k_{n, \ell _0}+1|^{\alpha '}}{\lambda _{n,k_{n, \ell _0}} \vee (|k_{n, \ell _0}|/{\tilde{\tau }})^{1/(1+\alpha )} } + \sum _{ k \ge 1} \frac{(k+1)^{\alpha '} - k^{\alpha '}}{\lambda _{n,k_{n, \ell _0}} \vee (k/{\tilde{\tau }})^{1/(1+\alpha )}} \right) , \end{aligned}$$

when $k_{n, \ell _0} \ge 1$, which occurs for *n* large enough. The first term of the last quantity is dominated by

$$\begin{aligned} (\lambda _{n,k_{n, \ell _0}} \vee (k_{n, \ell _0}/{\tilde{\tau }})^{1/(1+\alpha )})^{\alpha ' (1+\alpha )-1} \le (\lambda _{n,k_{n, \ell _0}})^{\alpha '(1+\alpha )-1}, \end{aligned}$$

which tends to zero when *n* goes to infinity, since $\lambda _{n,k_{n, \ell _0}}$ goes to infinity and $\alpha '(1+\alpha )-1 < 0$. Similarly,

$$\begin{aligned} \sum _{ k \ge 1} \frac{(k+1)^{\alpha '} - k^{\alpha '}}{\lambda _{n,k_{n, \ell _0}} \vee (k/{\tilde{\tau }})^{1/(1+\alpha )}} \underset{n \rightarrow \infty }{\longrightarrow } 0, \end{aligned}$$

by dominated convergence. Hence, we get (42).

We can now assume $L \cap (\ell _0, \infty ) \ne \emptyset$ and it remains to prove (43)–(45).

For $k \ge k_{\ell _0}$, let us define $\lambda _{n,k}$ and $\lambda _{k}$ as $\infty$ if these numbers are not well-defined: this does not change the quantities involved in (43). Moreover, for all $k \ge k_{\ell _0}$:

- If $\lambda _{k}$ is well-defined as a finite quantity, then $\lambda _{n,k}$ is also well-defined for *n* large enough and tends to $\lambda _{k}$ when *n* goes to infinity.
- If $\lambda _k = \infty$, then for all $A >0$, and for *n* large enough, one has $\lambda _{n,k} \notin [-A,A]$. Since for *n* large enough,
  $$\begin{aligned} \lambda _{n,k} \ge \lambda _{n, k_{\ell _0}}> \lambda _{k_{\ell _0}} -1> \ell _0 - 1 > 0, \end{aligned}$$
  one has $\lambda _{n,k} > A$: in other words, $\lambda _{n,k}$ tends to infinity with *n*.

We have just checked that with the convention made here, one has always $\lambda _{n,k}$ converging to $\lambda _{k}$ when *n* goes to infinity, for all $k \ge k_{\ell _0}$. Hence, (43) is a consequence of the dominated convergence theorem and the majorization:

$$\begin{aligned} \sum _{k \ge k_{\ell _0}} (\lambda _k \wedge \inf _{n \ge n_0} \lambda _{n,k})^{-2} < \infty , \end{aligned}$$

for some $n_0 \ge 1$. Now, there exists $n_0 \ge 1$ such that for all $n \ge n_0$, one has $k_{n,\ell _0} = k_{\ell _0}$, and then for all $k \ge 1 \vee k_{\ell _0}$, $\lambda _{k}> \ell _0 > 2$, $\lambda _{n,k} >2$ and $k \ge 1$, which implies the minorizations (46) and (47). Hence one gets (43), since

$$\begin{aligned} \sum _{k \ge 1} (k/{\tilde{\tau }})^{-2/(1+\alpha )} < \infty . \end{aligned}$$

Since (44) is easy to check, it remains to show (45), which can be rewritten as follows:

$$\begin{aligned} \sum _{ k \ge k_{\ell _0}+1} \Delta _{n,k} \left( \frac{1}{\lambda _{n,k-1} -z} - \frac{1}{\lambda _{n,k} - z} \right) \underset{n \rightarrow \infty }{\longrightarrow } \sum _{ k \ge k_{\ell _0}+1} \Delta _{k} \left( \frac{1}{\lambda _{k-1} -z} - \frac{1}{\lambda _{k} - z} \right) , \end{aligned}$$

where for $k \ge K_{n,\infty }$ (resp. $k \ge K_{\infty }$), one defines $\lambda _{k,n} := \infty$ (resp. $\lambda _{k} := \infty$). Note that with this convention, $\lambda _{n,k}$ tends to $\lambda _k$ when *n* goes to infinity, for all $k \ge k_{\ell _0}$. Note that each term of the left-hand side of this last convergence converges uniformly on $\{z \in {\mathbb {C}}, |z| < \ell _0/2\}$ towards the corresponding term in the right-hand side. Indeed, for *n* large enough, for all $k \ge k_{\ell _0}$, for $|z| < \ell _0/2$, and for $\lambda _k$, $\lambda _{n,k}$ finite,

$$\begin{aligned} \left| \frac{1}{\lambda _{n,k} - z} - \frac{1}{\lambda _{k} - z} \right|&= \frac{|\lambda _{k} - \lambda _{n,k}|}{|\lambda _{n,k} - z||\lambda _{k} - z|} \le \frac{4 |\lambda _{k} - \lambda _{n,k}|}{\lambda _{n,k} \lambda _{k} } \\&\le 4 \left| \frac{1}{\lambda _{n,k} } - \frac{1}{\lambda _{k}}\right| \underset{n \rightarrow \infty }{\longrightarrow } 0, \end{aligned}$$

this convergence, uniform in *z*, being in fact also true if $\lambda _{n,k}$ or $\lambda _k$ is infinite. Hence, one has, for all $k' > k_{\ell _0}+1$, the uniform convergence:

$$\begin{aligned} \sum _{ k_{\ell _0}+1 \le k \le k'} \Delta _{n,k} \left( \frac{1}{\lambda _{n,k-1} -z} - \frac{1}{\lambda _{n,k} - z} \right) \underset{n \rightarrow \infty }{\longrightarrow } \sum _{k_{\ell _0}+1 \le k \le k'} \Delta _{k} \left( \frac{1}{\lambda _{k-1} -z} - \frac{1}{\lambda _{k} - z} \right) , \end{aligned}$$

Hence, it is sufficient to check, for $n_0 \ge 1$ such that $k_{n,\ell _0} =k_{\ell _0}$ if $n \ge n_0$, that

48 $$\begin{aligned} \sup _{n \ge n_0, |z| < \ell _0/2} \left| \sum _{ k > k'} \Delta _{n,k} \left( \frac{1}{\lambda _{n,k-1} -z} - \frac{1}{\lambda _{n,k} - z} \right) \right| \underset{k' \rightarrow \infty }{\longrightarrow } 0 \end{aligned}$$

and

49 $$\begin{aligned} \sup _{|z| < \ell _0/2} \left| \sum _{ k > k'} \Delta _{k} \left( \frac{1}{\lambda _{k-1} -z} - \frac{1}{\lambda _{k} - z} \right) \right| \underset{k' \rightarrow \infty }{\longrightarrow } 0. \end{aligned}$$

Now, for $k' \ge 1 \vee (k_{\ell _0} + 1)$, $n \ge n_0$ and $|z| < \ell _0/2$, one has

$$\begin{aligned} \left| \sum _{ k> k'} \Delta _{n,k} \left( \frac{1}{\lambda _{n,k-1} -z} - \frac{1}{\lambda _{n,k} - z} \right) \right|&\le \sum _{ k> k'} |\Delta _{n,k}| \, \left| \frac{1}{\lambda _{n,k-1} -z} - \frac{1}{\lambda _{n,k} - z} \right| \\&\le 4 c \sum _{ k> k'} k^{\alpha '} \, \left( \frac{1}{\lambda _{n,k-1}} - \frac{1}{\lambda _{n,k}} \right) \\&= 4 c \left( \frac{(k'+1)^{\alpha '}}{\lambda _{n,k'}} + \sum _{k> k'} \frac{(k+1)^{\alpha '} - k^{\alpha '}}{\lambda _{n,k}} \right) \\&\le 4 c \left( \frac{(k'+1)^{\alpha '}}{(k'/{\tilde{\tau }})^{1/(1+\alpha )}} + \sum _{k > k'} \frac{(k+1)^{\alpha '} - k^{\alpha '}}{(k/{\tilde{\tau }})^{1/(1+\alpha )}} \right) \\&\quad \underset{k' \rightarrow \infty }{\longrightarrow } 0, \end{aligned}$$

which proves (48). One shows (49) in an exactly similar way, which finishes the proof of the convergence of $S_n$ towards $S+{\bar{\gamma }}h$, uniformly on compact sets for the distance *d*. $\square$

### Proof of the formulas (21)–(24)

By observing the sign of the imaginary parts $\mathfrak {I}(S_n)$ and $\mathfrak {I}(S)$, we deduce that the sets $\Xi '_n$ and $\Xi '$ are included in ${\mathbb {R}}$. Moreover, the derivatives $S_n'$ and $S'$ are strictly positive, respectively on ${\mathbb {R}} {\backslash }L_n$ and ${\mathbb {R}} {\backslash }L$, and all the left (resp. right) limits of $S_n$ and *S* at their poles are equal to $+ \infty$ (resp. $-\infty$). We deduce that the support of $\Xi '_n$ (resp. $\Xi '$) strictly interlaces with the points in $L_n$ (resp. *L*).

The Eq. (21) is a direct consequence of the convergence of $S_n$ towards $S+{\bar{\gamma }}h$, as written in the statement of Theorem (21). More precisely, for two points *a* and *b* ($a < b$) not in the support of $\Xi '$ and such that $\Xi '((a,b)) = k$, there exist real numbers $a = q_0< q_1< q_2< \dots< q_{2k}< b = q_{2k+1}$ such that $-\infty< S(q_{2j-1})< h'< S(q_{2j}) < \infty$ for $j \in \{1, \dots , k\}$, which implies that these inequalities are also satisfied for $S_n - {\bar{\gamma }}h$ instead of *S* if *n* is large enough: one has $\Xi '_n((a,b)) \ge k$. On the other hand, since the support of $\Xi '$ has exactly one point on each interval $[q_{2j-1}, q_{2j}]$ ($1 \le j \le k$) and no point on the intervals $[q_{2j},q_{2j+1}]$ ($0 \le j \le k$), one deduces that *S* is bounded on the intervals $[q_{2j-1}, q_{2j}]$ and bounded away from $h'$ on the intervals $[q_{2j},q_{2j+1}]$. These properties remain true for $S_n - {\bar{\gamma }}h$ if *n* is large enough, and one easily deduces that $\Xi '_n((a,b)) \le k$.

The properties (22)–(24) can be deduced from the property of interlacing. More precisely, for $\ell \ge 1$,

$$\begin{aligned} \int _{{\mathbb {R}}} \frac{{\mathbf {1}}(\lambda > \ell )}{\lambda ^{1+\alpha }} \, d \Xi '_n(\lambda ) = \sum _{\lambda \in L'_n \cap (\ell , \infty )} \frac{1}{\lambda ^{1+\alpha }}, \end{aligned}$$

where $L'_n$ is the support of $\Xi '_n$. By the interlacing property, if $L'_n \cap (\ell , \infty )$ is not empty and if its smallest element is $\lambda ' > \ell$, then it is possible to define an injection between $(L'_n \cap (\ell , \infty ) ) {\backslash }\{\lambda '\}$ and $L_n \cap (\ell , \infty )$, such that the image of each point is smaller than this point. One deduces

$$\begin{aligned} \int _{{\mathbb {R}}} \frac{{\mathbf {1}}(\lambda> \ell )}{\lambda ^{1+\alpha }} \, d \Xi '_n(\lambda ) \le \frac{1}{\lambda '} + \sum _{\lambda \in L_n \cap (\ell , \infty )} \frac{1}{\lambda ^{1+\alpha }} \le \frac{1}{\ell } + \int _{{\mathbb {R}}} \frac{{\mathbf {1}}(\lambda > \ell )}{\lambda ^{1+\alpha }} \, d \Xi _n(\lambda ). \end{aligned}$$

By looking similarly at the integral for $\lambda < -\ell$, one deduces

$$\begin{aligned} \int _{{\mathbb {R}}} \frac{{\mathbf {1}}(|\lambda |> \ell )}{|\lambda |^{1+\alpha }} \, d \Xi '_n(\lambda ) \le \frac{2}{\ell } + \int _{{\mathbb {R}}} \frac{{\mathbf {1}}(|\lambda | > \ell )}{|\lambda |^{1+\alpha }} \, d \Xi _n(\lambda ) \le \frac{2}{\ell } +\tau _{\ell } \underset{\ell \rightarrow \infty }{\longrightarrow } 0, \end{aligned}$$

which proves (22) for the measure $\Xi '_n$.

By a similar argument, for $\ell ''> \ell ' > \ell \ge 1$,

$$\begin{aligned} \int _{{\mathbb {R}}} \frac{{\mathbf {1}}(\ell ' \le \lambda< \ell '')}{\lambda } \, d \Xi '_n(\lambda ) \le \int _{{\mathbb {R}}} \frac{{\mathbf {1}}(\ell ' \le \lambda < \ell '')}{\lambda } \, d \Xi _n(\lambda ) + \frac{1}{\ell '}, \end{aligned}$$

and one has the similar inequalities obtained by exchanging $\Xi _n$ and $\Xi '_n$, and by changing the sign of $\lambda$. Hence,

$$\begin{aligned} \left| \int _{{\mathbb {R}}} \frac{{\mathbf {1}}(\ell ' \le |\lambda |< \ell '')}{|\lambda |} \, d \Xi '_n(\lambda ) - \int _{{\mathbb {R}}} \frac{{\mathbf {1}}(\ell ' \le |\lambda |< \ell '')}{|\lambda |} \, d \Xi _n(\lambda ) \right| \le \frac{4}{\ell '}. \end{aligned}$$

This inequality and the existence of the limit $h_{n,\ell }$ for the measure $\Xi _n$ implies that

$$\begin{aligned} \underset{\ell ' \wedge \ell '' \rightarrow \infty }{\limsup } \left| \int _{{\mathbb {R}}} \frac{{\mathbf {1}}(\ell< |\lambda |< \ell '')}{|\lambda |} \, d \Xi '_n(\lambda ) - \int _{{\mathbb {R}}} \frac{{\mathbf {1}}(\ell< |\lambda |< \ell ')}{|\lambda |} \, d \Xi '_n(\lambda ) \right| = 0, \end{aligned}$$

and then the limit $h'_{n,\ell }$ given by (24) exists, and we get the existence of $h'_{\ell }$ in a similar way. Moreover, for all $\ell \ge 1$, one gets the majorization:

50 $$\begin{aligned} \left| \underset{\ell ' \rightarrow \infty }{\lim } \int _{{\mathbb {R}}} \frac{{\mathbf {1}}(\ell< |\lambda |< \ell ')}{|\lambda |} \, d \Xi '_n(\lambda ) - \underset{\ell ' \rightarrow \infty }{\lim } \int _{{\mathbb {R}}} \frac{{\mathbf {1}}(\ell< |\lambda |< \ell ')}{|\lambda |} \, d \Xi _n(\lambda ) \right| \le \frac{4}{\ell }, \nonumber \\ \end{aligned}$$

and a similar inequality without the index *n*. Hence, we have

$$\begin{aligned} |h'_{n,\ell } - h_{n,\ell }| = O(\ell ^{-1}), \; |h'_{\ell } - h_{\ell }| = O(\ell ^{-1}). \end{aligned}$$

We then deduce (23) from (18) (with the same value of *h*), provided that we check the existence of the limit of $h'_{n,\ell }$ when $n \rightarrow \infty$, which is involved in (23):

51 $$\begin{aligned} \lim _{n \rightarrow \infty } \underset{\ell ' \rightarrow \infty }{\lim } \int _{{\mathbb {R}}} \frac{{\mathbf {1}}(\ell< |\lambda |< \ell ')}{|\lambda |} \, d \Xi '_n(\lambda ) \end{aligned}$$

for each $\ell$ such that $\ell$ and $-\ell$ are not in the support of $\Xi '$. Let us first assume that $\ell$ and $-\ell$ are also not in the support of $\Xi$. We have, for all $\ell '' > \ell$,

52 $$\begin{aligned} \underset{\ell ' \rightarrow \infty }{\lim } \int _{{\mathbb {R}}} \frac{{\mathbf {1}}(\ell< |\lambda |< \ell ')}{|\lambda |} \, d \Xi '_n(\lambda )= & {} \int _{{\mathbb {R}}} \frac{{\mathbf {1}}(\ell< |\lambda | \le \ell '')}{|\lambda |} \, d \Xi '_n(\lambda ) \nonumber \\&\quad +\, \underset{\ell ' \rightarrow \infty }{\lim } \int _{{\mathbb {R}}} \frac{{\mathbf {1}}(\ell ''< |\lambda |< \ell ')}{|\lambda |} \, d \Xi '_n(\lambda ),\nonumber \\ \end{aligned}$$

and a similar equality with $\Xi '_n$ replaced by $\Xi _n$. Since $\ell$ and $-\ell$ are not in the support of $\Xi$ or $\Xi '$, the convergences of $\Xi _n$ towards $\Xi$ and of $\Xi '_n$ towards $\Xi '$ imply that the lower and upper limits (when *n* goes to infinity) of the first term of (52) (both with $\Xi '_n$ and with $\Xi _n$) differ by $O(1/\ell '')$. For the second term, the difference between the lower and upper limits should change only by $O(1/\ell '')$ when we replace (52) by the same equation with $\Xi _n$, thanks to (50). Hence, this observation is also true for the sum of the two terms. On the other hand, the existence of the limit of $h_{n,\ell }$ when *n* goes to infinity (for $\Xi _n$) implies that in (52) with $\Xi '_n$ replaced by $\Xi _n$, the difference between the upper and lower limit is zero. Therefore, the difference is $O(1/\ell '')$ without replacement of $\Xi '_n$ by $\Xi _n$ : letting $\ell '' \rightarrow 0$ gives the existence of the limit (51) for $-\ell , \ell$ not in the support of $\Xi$ and $\Xi '$. If $-\ell$ or $\ell$ is in the support of $\Xi$ (but not in the support of $\Xi '$), we observe that for some $\epsilon > 0$ and *n* large enough, there is no point in the supports of $\Xi '_n$ and $\Xi '$ in the intervals $\pm \ell + (-\epsilon , \epsilon )$, which implies that the integral involved in (51) does not change if we change $\ell$ by less than $\epsilon$. By suitably moving $\ell$, we can then also avoid the support of $\Xi$. $\square$

## Convergence of Hermite corners towards the bead process

In this section, we consider, for all $\beta > 0$, the Gaussian $\beta$ Ensemble, defined as a set of *n* points $(\lambda _j)_{1 \le j \le n}$ whose joint density, with respect to the Lebesgue measure is proportional to

$$\begin{aligned} e^{-\beta \sum _{k=1}^n \lambda _k/4} \prod _{j < k} |\lambda _j - \lambda _k|^{\beta }. \end{aligned}$$

We will use the following crucial estimate, proven in [15]:

### Theorem 23

For $-\infty \le \Lambda _1 < \Lambda _2 \le \infty$, let $N(\Lambda _1, \Lambda _2)$ be the number of points, between $\Lambda _1$ and $\Lambda _2$, of a Gaussian beta ensemble with *n* points, and let $N_{sc}(\Lambda _1, \Lambda _2)$ be *n* times the measure of $(\Lambda _1, \Lambda _2)$ with respect to the semi-circle distribution on the interval $[- 2\sqrt{n}, 2 \sqrt{n}]$:

$$\begin{aligned} N_{sc}(\Lambda _1, \Lambda _2) := \frac{n}{2\pi } \int _{\Lambda _1/\sqrt{n}}^{\Lambda _2/\sqrt{n}} \sqrt{(4 - x^2)_+} \, dx. \end{aligned}$$

Then,

$$\begin{aligned} {\mathbb {E}} [(N(\Lambda _1, \Lambda _2) - N_{sc}(\Lambda _1, \Lambda _2))^2] = O( \log (2 + (\sqrt{n}(\Lambda _2 - \Lambda _1) \wedge n))). \end{aligned}$$

For $\beta \in \{1,2,4\}$, the Gaussian $\beta$ Ensemble can be represented by the eigenvalues of real symmetric (for $\beta = 1$), complex Hermitian (for $\beta = 2$), or quaternionic Hermitian (for $\beta = 4$) Gaussian matrices. The law of the entries of these matrices, corresponding respectively to the Gaussian Orthogonal Ensemble, the Gaussian Unitary Ensemble and the Gaussian Symplectic Ensemble, are given as follows:

- The diagonal entries are real-valued, centered, Gaussian with variance $2/\beta$.
- The entries above the diagonal are real-valued for $\beta = 1$, complex-valued for $\beta = 2$, quaternion-valued for $\beta = 4$, with independent parts, centered, Gaussian with variance $1/\beta$.
- All the entries involved in the previous items are independent.

By considering the top-left minors $A_n$ of an infinite random matrix *A* following the law described just above, and their eigenvalues, we get a family of sets of points, the *n*th set following the G$\beta$E of order *n*. Conditionally on the matrix $A_n$, whose eigenvalues are denoted $(\lambda _1, \dots , \lambda _n)$, supposed to be distinct (this holds almost surely), the law of the eigenvalues of $A_{n+1}$ can be deduced by diagonalizing $A_n$ inside $A_{n+1}$, which gives a matrix of the form

$$\begin{aligned} \left( \begin{array}{lllll} \lambda _1 &{}\quad 0 &{}\quad \cdots &{}\quad 0 &{}\quad g_1 \\ 0 &{}\quad \lambda _2 &{}\quad \cdots &{}\quad 0 &{}\quad g_2 \\ \vdots &{}\quad \vdots &{}\quad \ddots &{}\quad \vdots &{}\quad \vdots \\ 0 &{}\quad 0 &{}\quad \cdots &{}\quad \lambda _n &{}\quad g_n \\ \overline{g_1} &{}\quad \overline{g_2} &{}\quad \cdots &{}\quad \overline{g_n} &{}\quad g \end{array} \right) , \end{aligned}$$

where $g_1, \dots g_n, g$ are independent, centered Gaussian, *g* being real-valued with variance $2/\beta$, $g_1, \dots , g_n$ being real-valued of variance 1 for $\beta = 1$, complex-valued with independent real and imaginary parts of variance 1/2 for $\beta = 2$, quaternion-valued with independent parts of variance 1/4 for $\beta = 4$. Expanding the characteristic polynomial and dividing by the product of $\lambda _j - z$ for $1 \le j \le \lambda _n$, we see that the eigenvalues of $A_{n+1}$ are the solutions of the equation:

$$\begin{aligned} g - z - \sum _{j=1}^n \frac{|g_j|^2}{ \lambda _j - z} = 0. \end{aligned}$$

Hence, if for $n \ge 1$, we consider the eigenvalues of the matrices $(A_{n+k})_{k \ge 0}$, we get an inhomogeneous Markov chain defined as follows:

- The first set corresponds to the $G \beta E$ with *n* points.
- Conditionally on the sets of points indexed by $0, 1, \dots , k$, the set indexed by *k* containing the distinct points $\lambda _1, \dots , \lambda _{n+k}$, the set indexed by $k+1$ contains the zeros of
  $$\begin{aligned} g - z - \sum _{j=1}^{n+k} \frac{(2/\beta ) \gamma _{j}}{ \lambda _j - z}, \end{aligned}$$
  *g* being centered, Gaussian of variance $2/\beta$, $\gamma _j$ being a Gamma variable of parameter $\beta /2$, all these variables being independent.

Similar expressions of eigenvalues of successive minors of random matrices in terms of zeros of meromorphic functions can be found in the literature: for more detail, we refer to articles by Gelfand and Naimark [9], Baryshnikov [2], Neretin [14], Okounkov and Reshetikhin [16].

The Markov chain above can be generalized to all $\beta > 0$: this can be viewed as the “eigenvalues of the G$\beta$E minors”. In fact, what we obtain is equivalent (with suitable scaling) to the Hermite $\beta$ corners introduced by Gorin and Shkolnikov [10]. This fact is due to the following result, proven (up to scaling) in [8, Proposition 4.3.2]:

### Proposition 24

The density of transition probability from the set $(\lambda _1, \dots , \lambda _n)$ to the set $(\mu _1, \dots , \mu _{n+1})$, subject to the interlacement property

$$\begin{aligned} \mu _1< \lambda _1< \mu _2< \cdots< \mu _{n}< \lambda _n < \mu _{n+1}, \end{aligned}$$

is proportional to

$$\begin{aligned}&\prod _{1 \le p< q \le n+1} (\mu _q - \mu _p) \prod _{1 \le p < q \le n} (\lambda _q - \lambda _p)^{1- \beta } \\&\quad \prod _{1 \le p \le n, 1 \le q \le n+1} |\mu _q - \lambda _p|^{\beta /2 - 1}e^{- \frac{\beta }{4} \left( \sum _{1 \le q \le n+1} \mu _q^2 - \sum _{1 \le p \le n} \lambda _p^2 \right) }. \end{aligned}$$

As explained in [10], the marginals of the Hermite $\beta$ corner correspond to the Gaussian $\beta$ Ensemble, which implies the following:

### Proposition 25

For all $\beta > 0$, the set of $n+k$ points corresponding to the step *k* of the Markov chain just above has the distribution of the Gaussian $\beta$ Ensemble of dimension $n+k$, if the initial distribution is the Gaussian $\beta$ Ensemble of dimension *n*. In particular, if we take $n=1$, we get a coupling of the $G \beta E$ in all dimensions.

Now, we show that a suitable scaling limit of this Markov chain is the $\beta$-bead process introduced in the paper.

We choose $E \in (-2,2)$ (this corresponds to the bulk of the spectrum), $n \ge 1$, and we center the spectrum around the level $E \sqrt{n}$. The expected density of eigenvalues around this level is approximated by $\sqrt{n} \rho _{sc}(E)$, where $\rho _{sc}$ is the density of the semi-circular distribution. In order to get an average spacing of $2\pi$, we should then scale the eigenvalues by a factor $2 \pi \sqrt{n} \rho _{sc}(E) = \sqrt{n(4 - E^2)}$. For $k \ge 0$, we then consider the simple point measure $\Xi _n^{(k)}$ given by putting Dirac masses at the points $( \lambda ^{(n,k)}_j - E\sqrt{n}) \sqrt{n (4 - E^2)}$, where $(\lambda ^{(n,k)}_j)_{1 \le j \le n+k}$ is the set of $n+k$ points obtained at the step *k* of the Markov chain above. The sequence of measures $\Xi _n^{(k)}$ can be recovered as follows:

- For $k = 0$, $\Xi _n^{(0)}$ corresponds to the point measure associated with the suitably rescaled G$\beta$E point process, with *n* points.
- Conditionally on $\Xi _n^{(k)}$, $\Xi _n^{(k+1)}$ is obtained by taking the zeros of
  $$\begin{aligned} - \frac{E}{ \sqrt{4 - E^2}} + \frac{g^{(k)}}{\sqrt{n(4 -E^2)}} - \frac{z}{n(4-E^2)} - \int \frac{1}{ \lambda - z} d \Lambda _n^{(k)} (\lambda ), \end{aligned}$$
  where $g^{(k)}$ is a centered Gaussian variable of variance $2/\beta$, and $\Lambda _n^{(k)}$ is the weighted version of $\Xi _n^{(k)}$, the weights being i.i.d. with distribution corresponding to $2/\beta$ times a Gamma variable of parameter $\beta /2$.

We are now able to prove the following result:

### Theorem 26

The Markov chain $(\Xi _n^{(k)})_{k \ge 0}$ converges in law to the Markov chain defined in Theorem 13, for the topology of locally weak convergence of locally finite measures on ${\mathbb {R}} \times {\mathbb {N}}_0$ (i.e. convergence of the integrals against continuous functions with compact support), and for the level

$$\begin{aligned} h = - \frac{E}{ \sqrt{4 - E^2}}. \end{aligned}$$

For $\beta = 2$ and $h \in {\mathbb {R}}$ fixed, the law of the Markov chain of Theorem 13 corresponds (after dividing the points by 2) to the bead process introduced by Boutillier, with parameter

$$\begin{aligned} \gamma = - \frac{h}{\sqrt{1 + h^2}}, \end{aligned}$$

if we take the notation of [6].

### Proof

By the result of Valkó and Virág, $\Xi _n^{(0)}$ converges in distribution to the ${\text {Sine}}_{\beta }$ point process.

Hence, the family, indexed by *n*, of the distributions of $(\Xi _n^{(0)})_{n \ge 1}$, is tight in the space of probability measures on ${\mathcal {M}}({\mathbb {R}})$, ${\mathcal {M}}({\mathbb {R}})$ being the space of locally finite measures on the Borel sets of ${\mathbb {R}}$, endowed with the topology of locally weak convergence. Hence, for $\epsilon > 0$, there exists $(C_K)_{K \in {\mathbb {N}}}$ such that with probability at least $1 - \epsilon$, the number of points in $[-K,K]$ of $\Xi _n^{(0)}$ is at most $C_K$ for all $K \in {\mathbb {N}}$, independently of *n*. Since the points of $\Xi ^{(k)}_n$ interlace with those of $\Xi ^{(k-1)}_n$, the condition just above is satisfied with $\Xi ^{(k)}_n$ instead of $\Xi ^{(0)}_n$. Hence, the family, indexed by *n*, of the laws of $(\Xi ^{(k)}_n)_{k \ge 0}$ is tight in the space of probability measures on ${\mathcal {M}}({\mathbb {R}} \times {\mathbb {N}}_0)$, ${\mathcal {M}}({\mathbb {R}} \times {\mathbb {N}}_0)$ being the space of locally finite measures on ${\mathbb {R}} \times {\mathbb {N}}_0$, again endowed with the topology of locally weak convergence. Recall that this property of tightness means that for all $\epsilon > 0$, there exists a compact subset ${\mathcal {K}}_{\epsilon }$ of ${\mathcal {M}}({\mathbb {R}} \times {\mathbb {N}}_0)$ such that the locally finite measure on ${\mathbb {R}} \times {\mathbb {N}}_0$ corresponding to $\Xi ^{(0)}_n$ is in ${\mathcal {K}}_{\epsilon }$ with probability at least $1-\epsilon$. For all $\epsilon > 0$, the compact set ${\mathcal {K}}_{\epsilon }$ exists because we can find $(C_{K,k})_{K \in {\mathbb {N}}, k \in {\mathbb {N}}_0}$ such that with probability at least $1-\epsilon$, $\Xi _n^{(k)}$ has at most $C_{K,k}$ points in the interval $[-K,K]$, for all *k*, *K* and independently of *n*.

From the tightness and Prokhorov’s theorem, it is enough to prove that the law of the Markov chain of Theorem 13 is the only possible limit for a subsequence of the laws of $(\Xi ^{(k)}_n)_{k \ge 1}$. Let us consider such a subsequence which converges in law. We define the following random variable

$$\begin{aligned} Y_n := \sup _{\ell \ge 0} ( 1+ \ell )^{-3/4} (|{\widetilde{\Xi }}^{(0)}_n ([0, \ell ]) | + |{\widetilde{\Xi }}^{(0)}_n ([-\ell , 0]) |) \end{aligned}$$

where

$$\begin{aligned} {\widetilde{\Xi }}^{(0)}_n ([a,b]) := \Xi ^{(0)}_n([a,b]) - N_{sc} \left( \left[ E \sqrt{n} + \frac{a}{\sqrt{n(4-E^2)}},E\sqrt{n} + \frac{b}{\sqrt{n(4-E^2)}} \right] \right) , \end{aligned}$$

for

$$\begin{aligned} N_{sc} (\Lambda _1, \Lambda _2) := \frac{n}{2 \pi } \int _{\Lambda _1/\sqrt{n}}^{\Lambda _2/\sqrt{n}} \sqrt{(4-x^2)_+} dx. \end{aligned}$$

The family $(Y_n)_{n \ge 0}$ is tight. Indeed, by Theorem 23,

$$\begin{aligned}&{\mathbb {E}} [(1+ \ell )^{-3/2} (|{\widetilde{\Xi }}^{(0)}_n ([0, \ell ]) |^2 + |{\widetilde{\Xi }}^{(0)}_n ([-\ell , 0])|^2 )]\\&\quad = O \left( (1+ \ell )^{-3/2} \log \left( 2 + \sqrt{n} \frac{\ell }{\sqrt{n(4-E^2)}} \right) \right) , \end{aligned}$$

which shows that

$$\begin{aligned} {\mathbb {E}} \left[ \sum _{\ell =0}^{\infty } (1+ \ell )^{-3/2} (|{\widetilde{\Xi }}^{(0)}_n ([0, \ell ]) | + |{\widetilde{\Xi }}^{(0)}_n ([-\ell , 0])|)^2 \right] \le C_{E,\beta }, \end{aligned}$$

where $C_{E,\beta } < \infty$ depends only on *E* and $\beta$ (in particular, not on *n*). This implies that $(Y_n)_{n \ge 1}$ is tight.

The point processes $\Xi _n^{(k)}$, $k \ge 0$, are constructed from $\Xi _n^{(0)}$, and families $\gamma _{n,k,k'}$ of weights, $\gamma _{n,k,k'}$ being the weight, involved in the construction of $\Xi _n^{(k+1)}$, of the $(k')$th nonnegative point of $\Xi _n^{(k)}$ if $k' > 0$, the $(1-k')$th negative point of $\Xi _n^{(k)}$ if $k' \le 0$. Notice that in this discussion, for all infinite families of real-valued random variables, we can consider them as single random variables on ${\mathbb {R}}^I$ for a suitable infinite set *I*: in this case, the $\sigma$-algebra taken on ${\mathbb {R}}^I$ is the Borel $\sigma$-algebra associated to the topology of the pointwise convergence of the coordinates. All the variables $\gamma _{n,k,k'}$ are i.i.d., distributed like $2/\beta$ times a Gamma variable of parameter $\beta /2$, and independent of $\Xi _n^{(0)}$. We can consider the variables

$$\begin{aligned} Z_{n,k} = \sup _{m \ge 1} m^{-0.51} \left| m - \sum _{k' = 0}^{m-1} \gamma _{n,k,k'}\right| + \sup _{m \ge 1} m^{-0.51} \left| m - \sum _{k' = -m}^{-1} \gamma _{n,k,k'} \right| . \end{aligned}$$

By classical tail estimates of the Gamma variables, or by the law of iterated logarithm, $Z_{n,k} < \infty$ almost surely, and since its law does not depend on *n* and *k*, $(Z_{n,k})_{n \ge 1, k \ge 0}$ is a tight family of random variables.

Hence, $(Z_n := (Z_{n,k})_{k \ge 0} )_{n \ge 1}$ is a tight family of random variables on ${\mathbb {R}}^{{\mathbb {N}}_0}$, endowed with the $\sigma$-algebra generated by the sets $\{(z_k)_{k \ge 0}, z_0 \in A_0, z_1 \in A_1, \dots , z_p \in A_p\}$ for $p \ge 0$ and $A_j \in {\mathcal {B}} ({\mathbb {R}})$.

Let us go back to our subsequence of $(\Xi _n^{(k)})_{k \ge 0}$ which converges in law. If we join $(\gamma _{n,k, k'})_{k \ge 0, k' \in {\mathbb {Z}}}$, $Y_n$ and $Z_n$, we still get a tight family of probability measures on a suitable probability space. Hence, we can find a sub-subsequence for which the family of random variables $((\Xi _n^{(k)})_{k \ge 0}, (\gamma _{n,k, k'})_{k \ge 0, k' \in {\mathbb {Z}}}, Y_n, Z_n)$ converges in law, and a fortiori $(\Xi _n^{(0)}, (\gamma _{n,k, k'})_{k \ge 0, k' \in {\mathbb {Z}}}, Y_n, Z_n)$ converges in law. We can now apply Skorokhod representation theorem (see [4, Theorem 6.7]). Indeed, the random variables take values in the product space ${\mathcal {M}} ({\mathbb {R}} \times {\mathbb {N}}_0) \times {\mathbb {R}}^I$ for a suitable countable set *I*. The space ${\mathbb {R}}^I$ is separable since we consider the topology of pointwise convergence. The space of probability measures on the separable metric space ${\mathbb {R}} \times {\mathbb {N}}_0$, endowed with the topology of the weak convergence, is separable. We deduce that it is also the case for the space of finite measures on ${\mathbb {R}} \times {\mathbb {N}}_0$, after multiplying the probability measures of a dense sequence by all positive rational numbers. Since the finite measures on ${\mathbb {R}} \times {\mathbb {N}}_0$ are dense in the space of locally finite measures on ${\mathbb {R}} \times {\mathbb {N}}_0$, for the topology of locally weak convergence (because the test functions have compact support), we deduce that ${\mathcal {M}} ({\mathbb {R}} \times {\mathbb {N}}_0)$ is a separable space. By Skorokhod representation theorem, the family of random variables $(\Xi _n^{(0)}, (\gamma _{n,k, k'})_{k \ge 0, k' \in {\mathbb {Z}}}, Y_n, Z_n)$ has the same law as some family $(\Xi _n^{'(0)}, (\gamma '_{n,k, k'})_{k \ge 0, k' \in {\mathbb {Z}}}, Y'_n, Z'_n)$ which converges almost surely along the same sub-subsequence as the one for which the family $((\Xi _n^{(k)})_{k \ge 0}, (\gamma _{n,k, k'})_{k \ge 0, k' \in {\mathbb {Z}}}, Y_n, Z_n)$ converges in law. Note that $Y'_n$ is function of $\Xi _n^{'(0)}$ and $Z'_n$ is function of the weights $\gamma '_{n,k, k'}$. Since we know that $\Xi _n^{'(0)}$ converges in law to a ${\text {Sine}}_{\beta }$ process, its almost sure limit is a simple point measure.

From the boundedness of $Y'_n$ along our sub-subsequence, and by Proposition 16, we deduce that the part of Theorem 21 concerning $\Xi _n^{'(0)}$ is satisfied, with

$$\begin{aligned} h = \underset{\ell \rightarrow \infty }{\lim } \, \underset{n \rightarrow \infty }{\lim } h^{sc}_{n, \ell }, \end{aligned}$$

for

$$\begin{aligned} h^{sc}_{n, \ell } = \int _{(-\infty , -\ell ] \cup [\ell , \infty )} \frac{1}{\lambda } d N_{sc} \left( E\sqrt{n} + \frac{\lambda }{\sqrt{n (4 - E^2)}} \right) . \end{aligned}$$

Indeed, the convergence of $\Xi _n^{'(0)}$ to a ${\text {Sine}}_{\beta }$ process $\Xi ^{'(0)}$ gives (15). Moreover,

$$\begin{aligned} \Xi _n^{'(0)} ([-\ell ,\ell ]) = O ( 1 + \ell + Y'_n (1+\ell )^{3/4}) =O ( Y'_n (1+\ell )) \end{aligned}$$

by the boundedness of the semi-circle density and the definition of $Y'_n$. We easily deduce (16) for any $\alpha \in (0,1)$ (with $\tau _{\ell }$ decaying like $\ell ^{-\alpha }$). From now, we choose $\alpha$ such that $0< \alpha < 49/51$.

The integral involved in the definition of $h_{n, \ell }$ can be written as the sum of an integral with respect to the semi-circle distribution and an integral with respect to $d {\widetilde{\Xi }}^{'(0)}_n$, where ${\widetilde{\Xi }}^{'(0)}_n$ is the signed measure involved in the definition of $Y'_n$:

$$\begin{aligned} {\widetilde{\Xi }}^{'(0)}_n ([a,b]) := \Xi ^{'(0)}_n([a,b]) - N_{sc} \left( \left[ E \sqrt{n} + \frac{a}{\sqrt{n(4-E^2)}},E\sqrt{n} + \frac{b}{\sqrt{n(4-E^2)}} \right] \right) . \end{aligned}$$

The integral with respect to the semi-circle distribution tends to $h^{sc}_{n, \ell }$ when $\ell ' \rightarrow \infty$, which itself tends to *h* when $n \rightarrow \infty$ and then $\ell \rightarrow \infty$. The integral with respect to $d {\widetilde{\Xi }}^{'(0)}_n$ can be transformed via an integration by part, as follows. If we restrict the integral to $\lambda > 0$, we get

$$\begin{aligned} \int _{{\mathbb {R}}} \frac{{\mathbf {1}} (\ell< \lambda < \ell ')}{\lambda } d {\widetilde{\Xi }}^{'(0)}_n = \left[ \, \frac{ {\widetilde{\Xi }}^{'(0)}_n ( [0, \lambda ])}{\lambda } \right] _{\ell }^{(\ell ')-} - \int _{\ell }^{\ell '} \frac{ {\widetilde{\Xi }}^{'(0)}_n ( [0, \lambda ]) }{\lambda ^2} \, d \lambda . \end{aligned}$$

Since ${\widetilde{\Xi }}^{'(0)}_n ( [0, \lambda ])$ is dominated by $(1+\lambda )^{3/4}$ because of the boundedness of $Y'_n$, we can let $\ell ' \rightarrow \infty$ and we get the limit

$$\begin{aligned} \left[ \, \frac{ {\widetilde{\Xi }}^{'(0)}_n ( [0, \lambda ])}{\lambda } \right] _{\ell }^{\infty } - \int _{\ell }^{\infty } \frac{ {\widetilde{\Xi }}^{'(0)}_n ( [0, \lambda ]) }{\lambda ^2} \, d \lambda . \end{aligned}$$

From (15) and dominated convergence, due to the fact that $Y'_n$ is bounded along the subsequence we consider, we get that the last expression converges to

53 $$\begin{aligned} \left[ \, \frac{ {\widetilde{\Xi }}^{'(0)} ( [0, \lambda ])}{\lambda } \right] _{\ell }^{\infty } - \int _{\ell }^{\infty } \frac{ {\widetilde{\Xi }}^{'(0)} ( [0, \lambda ]) }{\lambda ^2} \, d \lambda . \end{aligned}$$

when

$$\begin{aligned} {\widetilde{\Xi }}^{'(0)} [0, \lambda ]= & {} \Xi ^{'(0)} [0,\lambda ] - \lim _{n \rightarrow \infty } N_{sc} \left( \left[ E \sqrt{n}, E\sqrt{n} + \frac{\lambda }{\sqrt{n(4-E^2)}} \right] \right) \\= & {} \Xi ^{'(0)} [0,\lambda ] - \frac{\lambda }{2 \pi }. \end{aligned}$$

Notice that there is no problem of discontinuity for the bracket at $\ell$, because we take limits for $\ell$ outside the support of $\Xi ^{'(0)}$. Now, it is clear that (53) tends to zero when $\ell \rightarrow \infty$. Similarly

$$\begin{aligned} \int _{{\mathbb {R}}} \frac{{\mathbf {1}} (-\ell '< \lambda < -\ell )}{\lambda } d {\widetilde{\Xi }}^{'(0)}_n \end{aligned}$$

tends to zero, after taking successive limits $\ell ' \rightarrow \infty$, $n \rightarrow \infty$, $\ell \rightarrow \infty$. We then deduce (17) and (18) for $\Xi ^{'(0)}_n$, by adding the limits of the integral with respect to the semi-circle distribution and the integral with respect to $d {\widetilde{\Xi }}^{'(0)}_n$. For $\Xi ^{'(0)}$, the method is the same: we use Proposition 16 in order to estimate the point counting of the ${\text {Sine}}_{\beta }$ process, and we replace the semi-circle distribution by $1/2 \pi$ times the Lebesgue measure, which ensures the vanishing limit in (18).

We can now compute *h* explicitly. We have:

$$\begin{aligned}&d N_{sc} \left( E \sqrt{n} + \frac{\lambda }{\sqrt{n (4 - E^2)}} \right) = \frac{n}{2 \pi } d \left( \int _{-\infty }^{E+\left( \lambda /(n \sqrt{4 -E^2})\right) } \sqrt{(4 - x^2)_+} dx \right) \\&= \frac{1}{2 \pi \sqrt{4 - E^2}} \sqrt{\left[ 4 - \left( E +\left( \lambda /(n \sqrt{4 -E^2})\right) \right) ^2 \right] _+} d \lambda \end{aligned}$$

If we do a change of variable $\lambda = \mu n \sqrt{4 - E^2}$, we get

$$\begin{aligned} h_{n,\ell }^{sc} = \int _{(-\infty , - \ell / (n \sqrt{4 -E^2})] \cup [\ell / (n \sqrt{4 -E^2}), \infty )} \frac{1}{2 \pi \sqrt{4 - E^2}} \sqrt{[4 - (E+ \mu )^2]_+} \frac{d \mu }{ \mu }. \end{aligned}$$

Taking $n \rightarrow \infty$, we get a quantity independent of $\ell$, given by

$$\begin{aligned} h = \frac{1}{2 \pi \sqrt{4 - E^2}} \int _{{\mathbb {R}}} \sqrt{(4 - y^2)_+} \frac{dy}{y - E}, \end{aligned}$$

the integral in the neighborhood of *E* being understood as a principal value. From the value of the Stieltjes transform of the semi-circle law, we deduce

$$\begin{aligned} h = - \frac{E}{2 \sqrt{4 - E^2}}. \end{aligned}$$

From the boundedness of $Z'_{n,0}$, we deduce that the part of Theorem 21 concerning the weights is also satisfied for $\alpha ' = 0.51$, since by the assumption $0< \alpha < 49/51$ made before, we have $0< \alpha ' (1+\alpha ) < 1$.

Finally, in this theorem, it is almost surely possible to take $\lambda ^* = 0$, by the absolutely continuity of the densities of the ensembles which are considered.

All the assumptions of the theorem are satisfied. If we denote by $\Lambda _n^{'(0)}$ the measure constructed from $\Xi _n^{'(0)}$ and the weights $\gamma '_{n,0,k'}$ ($k' \in {\mathbb {Z}}$), and $\Lambda ^{'(0)}$ the measure constructed from the a.s. limits of these points and weights, we deduce that for an independent standard Gaussian variable $g^{(0)}$, the function

$$\begin{aligned} z \mapsto - \frac{E}{ \sqrt{4 -E^2}} - \int \frac{1}{ \lambda - z} d \Lambda _n^{'(0)} (\lambda ), \end{aligned}$$

and then also the function

$$\begin{aligned} z \mapsto - \frac{E}{ \sqrt{4 - E^2}} + \frac{g^{(0)}}{\sqrt{n(4 - E^2)}} - \frac{z}{n(4-E^2)} - \int \frac{1}{ \lambda - z} d \Lambda _n^{'(0)} (\lambda ), \end{aligned}$$

converges uniformly on compact sets, for the topology of the Riemann sphere given in Theorem 21, to the function

$$\begin{aligned} z \mapsto -\frac{E}{ \sqrt{4 -E^2}} - \int \frac{1}{ \lambda - z} d \Lambda ^{'(0)} (\lambda ) - h = -\frac{E}{2 \sqrt{4 - E^2}} - \int \frac{1}{ \lambda - z} d \Lambda ^{'(0)} (\lambda ). \end{aligned}$$

As in the proof of Theorem 21, we deduce that the point process $\Xi _n^{'(1)}$ given by

$$\begin{aligned} - \frac{E}{ \sqrt{4 - E^2}} + \frac{g^{(0)}}{\sqrt{n(4 - E^2)}} - \frac{z}{n(4-E^2)} - \int \frac{1}{ \lambda - z} d \Lambda _n^{'(0)} (\lambda ) = 0, \end{aligned}$$

locally weakly converges to the point process $\Xi ^{'(1)}$ given by

$$\begin{aligned} \underset{\ell \rightarrow \infty }{\lim } \int _{[-\ell , \ell ]} \frac{1}{ \lambda - z} d \Lambda ^{'(0)} (\lambda ) = -\frac{E}{2 \sqrt{4 -E^2}}. \end{aligned}$$

The points of $\Xi _n^{'(1)}$ and satisfy the assumptions of Theorem 21, since they interlace with those of $\Xi _n^{'(0)}$. It is also the same for the weights $\gamma '_{n,1,k'}$ ($k' \in {\mathbb {Z}}$), by the boundedness of $Z'_n$. We then deduce that for an independent Gaussian variable $g^{(1)}$, the point process $\Xi _n^{'(2)}$ given by

$$\begin{aligned} - \frac{E}{ \sqrt{4 -E^2}} + \frac{g^{(1)}}{\sqrt{n(4 -E^2)}} - \frac{z}{n(4-E^2)} - \int \frac{1}{ \lambda - z} d \Lambda _n^{'(1)} (\lambda ) = 0 \end{aligned}$$

locally weakly converges to the process $\Xi ^{'(2)}$ given by

$$\begin{aligned} \underset{\ell \rightarrow \infty }{\lim } \int _{[-\ell , \ell ]} \frac{1}{ \lambda - z} d \Lambda ^{'(1)} (\lambda ) = -\frac{E}{2 \sqrt{4 - E^2}}, \end{aligned}$$

where $\Lambda _n^{'(1)}$ is given by $\Xi _n^{'(0)}$ and the weights $\gamma '_{n,1,k'}$ and $\Lambda ^{'(1)}$ are given by their limits. We can then iterate the construction, which gives a family of point processes $\Xi _n^{'(k)}$ ($k \ge 0$), converging to $\Xi ^{'(k)}$. From the way we do this construction, we check that $(\Xi _n^{'(k)})_{k \ge 0}$ has the same law as $(\Xi _n^{(k)})_{k \ge 0}$, and that $\Xi ^{'(k)}$ has the same law as the generalized bead process introduced in the present paper (with level lines at $-E/2 \sqrt{4 - E^2}$). Hence, any subsequence of $((\Xi _n^{(k)})_{k \ge 0})_{n \ge 1}$ converging in law has a sub-subsequence tending in law to the generalized bead process. By tightness, we deduce the convergence of the whole sequence $((\Xi _n^{(k)})_{k \ge 0})_{n \ge 1}$. This gives the first part of the theorem, after doubling the weights and the value of *h*. The second part is deduced by using the convergence of the GUE minors towards the bead process introduced by Boutillier, proven in [1]. The factor 2 is due to the fact that the average density of points is $1/\pi$ in [6] and $1/2 \pi$ here. The value of the parameter $\gamma$ in [6] (*a* in [1]) corresponds to *E*/2 (the bulk corresponds to the interval $(-1,1)$ in [1] and to $(-2,2)$ in the present paper). We then have

$$\begin{aligned} h = - \frac{E}{\sqrt{4 - E^2}} = - \frac{\gamma }{\sqrt{1 - \gamma ^2}}, \end{aligned}$$

and finally

$$\begin{aligned} \gamma = - \frac{h}{\sqrt{1 + h^2}}. \end{aligned}$$

$$\square $$

## Data Availability

This manuscript has no associated data.
